# Use of canonical discriminant analysis to study signatures of selection in cattle

**DOI:** 10.1186/s12711-016-0236-7

**Published:** 2016-08-12

**Authors:** Silvia Sorbolini, Giustino Gaspa, Roberto Steri, Corrado Dimauro, Massimo Cellesi, Alessandra Stella, Gabriele Marras, Paolo Ajmone Marsan, Alessio Valentini, Nicolò Pietro Paolo Macciotta

**Affiliations:** 1 Dipartimento di Agraria, Sezione di Scienze Zootecniche, Università degli Studi di Sassari, V. le Italia, 9, 07100 Sassari, Italy; 2 Consiglio per la Ricerca e la Sperimentazione in Agricoltura, via Salaria 31, 00015 Monterotondo, Italy; 3 Consiglio Nazionale delle Ricerche - IBBA, Lodi, Italy; 4 Istituto di Zootecnica, Università Cattolica del Sacro Cuore, Piacenza, Italy; 5 Dipartimento per l’Innovazione dei Sistemi Biologici Agroalimentari e Forestali DIBAF, Università della Tuscia, Viterbo, Italy

## Abstract

**Background:**

Cattle include a large number of breeds that are characterized by marked phenotypic differences and thus constitute a valuable model to study genome evolution in response to processes such as selection and domestication. Detection of “signatures of selection” is a useful approach to study the evolutionary pressures experienced throughout history. In the present study, signatures of selection were investigated in five cattle breeds farmed in Italy using a multivariate approach.

**Methods:**

A total of 4094 bulls from five breeds with different production aptitudes (two dairy breeds: Italian Holstein and Italian Brown Swiss; two beef breeds: Piemontese and Marchigiana; and one dual purpose breed: Italian Simmental) were genotyped using the Illumina BovineSNP50 v.1 beadchip. Canonical discriminant analysis was carried out on the matrix of single nucleotide polymorphisms (SNP) genotyping data, separately for each chromosome. Scores for each canonical variable were calculated and then plotted in the canonical space to quantify the distance between breeds. SNPs for which the correlation with the canonical variable was in the 99th percentile for a specific chromosome were considered to be significantly associated with that variable. Results were compared with those obtained using an F_ST_-based approach.

**Results:**

Based on the results of the canonical discriminant analysis, a large number of signatures of selection were detected, among which several had strong signals in genomic regions that harbour genes known to have an impact on production and morphological bovine traits, including *MSTN*, *LCT*, *GHR*, *SCD*, *NCAPG*, *KIT*, and *ASIP*. Moreover, new putative candidate genes were identified, such as *GCK*, *B3GALNT1*, *MGAT1*, *GALNTL1*, *PRNP*, and *PRND*. Similar results were obtained with the F_ST_-based approach.

**Conclusions:**

The use of canonical discriminant analysis on 50 K SNP genotypes allowed the extraction of new variables that maximize the separation between breeds. This approach is quite straightforward, it can compare more than two groups simultaneously, and relative distances between breeds can be visualized. The genes that were highlighted in the canonical discriminant analysis were in concordance with those obtained using the F_ST_ index.

**Electronic supplementary material:**

The online version of this article (doi:10.1186/s12711-016-0236-7) contains supplementary material, which is available to authorized users.

## Background

During the long process of animal domestication that began approximately 8000 to 12,000 years ago, man and environment played a fundamental role in the evolution of livestock species [1]. DNA mutations, adaptation, migrations, and selection have affected the biological diversity of natural populations, leading to the prevailing variability in livestock [2]. Thus, domestic animal species represent a relevant model for genetic diversity studies. A particularly useful example is the large range of current cattle breeds that are characterized by wide phenotypic variation due to the intense artificial selection they have been subjected to in the last 50 years [3–5].

Studies on cattle are supported by the availability of whole-genome sequence data, of well-developed linkage maps, and of a pedigree structure that, for many breeds, allows for a precise dissection of the effects that underlie complex traits [6–8]. High-throughput genotyping techniques, together with developments in comparative genomics, have opened great opportunities for the study of genomic modifications due to natural and artificial selection. These selective pressures increase the frequency of the most favorable allele at a target locus. This process also affects allele frequencies at loci at nearby locations and results in a loss of heterozygosity across that chromosomal region [9–11]. This phenomenon, known as “hitchhiking”, leads to the formation of selective sweeps or “signatures of selection”, that are characterized by distributions of allele frequencies around favorable mutations that statistically differ from those expected by chance [12].

Several approaches have been proposed to study signatures of selection in cattle that differ in the metrics and statistical inferences used [13, 14]. They are all essentially based on assessment of differences in allelic or haplotype frequencies between populations (i.e. breeds, different geographic origins and distributions, levels of selection, production aptitudes). Akey [15] classified tests for detecting signatures of selection into: (1) spectrum tests that are based on the distribution of polymorphisms in specific regions; (2) tests that are based on linkage disequilibrium (LD) between adjacent loci, i.e. tests that consider haplotypes; and (3) tests that compare population structures, such as the popular fixation index (F_ST_), that was originally proposed by Wright [16]. Studies on signatures of selection in cattle and sheep have used all three classes of methods, with the majority being based on F_ST_ [17–20], although spectrum- [12, 21] and LD-based methods [14, 22] have also been used. Furthermore, integrated approaches that combine the detection of signatures of selection with genome-wide association studies have been applied in dairy cattle [23]. Recently, detection of signatures of selection based on LD has been implemented using whole-genome sequence data [24].

Genetic differences are often evaluated under the perspective of a single locus or an aggregate of a small number of loci because studies on signatures of selection are focused mainly on detection of single nucleotide polymorphisms (SNPs) that tag chromosomal positions where putative candidate genes may be located. However, since signatures of selection are the result of the interaction between selection pressure on a causative gene and LD with adjacent loci, analyzing the correlation structure between SNPs in a specific genomic region could represent an interesting starting point to quantify the existence of a signature of selection. Multivariate statistics offer a set of techniques to study the different aspects of correlation matrices, among which principal component analysis (PCA) has proven to be very efficient in extracting information from a set of multiple genetic markers and has been successfully used in several fields of genetics [14, 25, 26]. In the specific case of the detection of signatures of selection where predefined groups of individuals are compared, canonical discriminant analysis (CDA) is particularly appealing. CDA aims at quantifying the relationship between a categorical variable, i.e. the group the individual belongs to, and a set of independent variables [27]. As for PCA, CDA is based on the extraction of linear combinations of original variables. However, whereas PCA aims at explaining the maximum amount of variance, canonical variables (CVA) are generated to maximize the difference between groups. Once CVA are extracted, their structure (i.e. correlations between CVA and original variables) can be examined in order to identify SNPs that contribute most to the discrimination between breeds. CDA was recently proposed by Dimauro et al. [28] to select a reduced pool of SNPs that were able to distinguish bovine breeds.

The aim of our study was to detect the presence of signatures of selection in cattle by comparing five Italian breeds with different production aptitudes (Italian Holstein–Friesian, Italian Brown Swiss, Italian Simmental, Marchigiana and Piemontese) using CDA. In order to assess the reliability and power of the CDA-based approach for the detection of signatures of selection, a comparison with the fixation index (F_ST_) method was also performed.

## Methods

### Data

A total of 4094 bulls were genotyped using the Illumina BovineSNP50 v.1 beadchip [29]. These animals were from five bovine breeds that were characterized by different production aptitudes: two dairy breeds (Italian Holstein–Friesian n = 2092, Italian Brown Swiss n = 749), two beef breeds (Piemontese n = 364, and Marchigiana n = 410) and one dual purpose breed (Italian Simmental n = 479). DNA for genotyping was extracted from semen straws that were produced for artificial insemination (AI) and supplied by the Breed Associations of each of these five breeds. In this study, since animal manipulation was not necessary, approval by the Animal Care Committee was not requested.

Among the animals included in this study, none had more than 1000 missing genotypes. Only SNPs that mapped to autosomes were considered. Filtering of SNP genotypes was performed across all breeds based on missing data (<2.5 %) and minor allele frequency (<1 %). After filtering, 39,833 SNPs that were common among the five breeds were retained for further analyses.

### Canonical discriminant analysis (CDA)

Given the data matrix ${\mathbf{M}}_{{\left( {\text{nxp}} \right)}}$ of *p* markers measured on *n* animals from *k* breeds, the CDA derives linear combinations of SNPs that maximize the between-breed variation. The *i*-th CVA, can be written as:

1 $${\text{CVA}}_{\text{i}} = {\text{a}}_{{{\text{i}}1}} {\mathbf{m}}_{1} + {\text{a}}_{{{\text{i}}2}} {\mathbf{m}}_{2} + \cdots + {\text{a}}_{\text{ip}} {\mathbf{m}}_{\text{p}} ,$$

where ${\text{a}}_{\text{ip}}$ are the canonical coefficients, which indicate the partial contribution of each SNP to the discriminant function; m_i_ are the SNP genotypes. The vector of coefficients ${\mathbf{a}}_{\text{i}}$ for the i-th canonical variable is obtained by maximizing the ratio:

2 $$\frac{{{\mathbf{a}}_{\text{i}}^{\prime } {\mathbf{Ba}}_{\text{i}} }}{{{\mathbf{a}}^{\prime } {\mathbf{Wa}}_{\text{i}} }},$$

where **B** and **W** are the between- and within-group SNP (co)variance matrices, respectively [30]. The dimension of the canonical space is the smallest value between *k* and *p* minus 1. In the present work, five breeds were considered and therefore four CVA were extracted. The eigenvalue of each i-th CVA, i.e. the amount of variance explained by the CVA, is $\uprho_{\text{i}}^{2} /\left( {1 -\uprho_{\text{i}}^{2} } \right)$, where ρ_i_^2^ is the i-th squared canonical correlation. The eigenvalue can be interpreted as the ratio of the between-breed to the pooled within-breed variation.

In our study, CDA was carried out using the CANDISC procedure of SAS 9.2 (SAS/STAT^®^ Software version 9.2, SAS Institute, Inc., Cary), separately for each bovine chromosome (BTA for *Bos taurus* chromosome). Thus, **M** was the data matrix of SNP genotypes (coded as 0, 1, 2) with *n* = 4094 rows, i.e. the number of bulls. The number of columns *p* varied from 2610 for BTA1 to 796 for BTA29, respectively. The CDA requires a full rank correlation matrix. However, the rank of a rectangular matrix is less or equal to the minimum value of the number of rows and columns [31, 32] and genetic correlation matrices are often not full rank [33]. Thus, the genome-wide SNP correlation matrix is singular. Conducting the CDA by chromosome mitigates these problems, while considering the substantial biological orthogonality among chromosomes [34].

Scores for each CVA and for each individual were calculated and then plotted in the canonical space. Differences between breeds were measured by the Mahalanobis distance, which expresses the distance between the centroids of each group.

The meaning of the extracted canonical variables was assessed by examining correlations between SNP genotypes and CVA scores within each chromosome [35, 36]. Some authors suggest that canonical coefficients instead of correlations should be used to assess relationships between CVA and original variables [28, 30]. However, when CDA is performed on a large number of variables that are characterized by a particular variability (i.e. SNP genotypes can have only three values), it is reasonable to expect that the pattern of the canonical coefficients may not be very simple to interpret.

SNPs that were considered as “relevant”, i.e., as possible indicators of signatures of selection, were identified in two steps. First, SNPs for which correlations with CVA were in the 99th percentile for a given chromosome were selected [25]. However, provided that each canonical variable explains a different amount of the variance, the final number of SNPs that was retained for each CVA was proportional to the ratio between its eigenvalue and the eigenvalue of the first CVA for the given chromosome. For example, BTA2 comprised 2110 SNPs and the eigenvalues of the first and second CVA were equal to 814.2944 and 495.8699, respectively. Thus, the retained SNPs were the top 1 % (i.e., 21) for the first CVA and 21*(495.8699/814.2944) = 13 for the second CVA, respectively.

### Fixation index (F_ST_) analysis

In order to compare the results of the CDA with a commonly applied method for detection of signatures of selection, the fixation index (F_ST_) was calculated at each locus for all the pairwise (n = 10) between breed comparisons using the formula proposed by Nei [37]:

$${\text{F}}_{\text{ST}} = \frac{{\left( {{\text{H}}_{\text{T}} - {\text{H}}_{\text{S}} } \right)}}{{{\text{H}}_{\text{T}} }},$$

where ${\text{H}}_{\text{T}}$ is the weighted expected heterozygosity calculated considering the two breeds as a single population; ${\text{H}}_{\text{S}}$ is the same parameter calculated by considering the two breeds separately. Raw F_ST_ values were smoothed using a locally weighted scatterplot smoothing (LOWESS) regression, combined with a control chart approach [17]. A SNP was declared significant if the corresponding F_ST_ value exceeded the threshold of 3 standard deviations (σ) from the mean. Significant SNPs detected by the F_ST_ approach were compared with those identified by CDA.

Annotated genes within the genomic regions that contained the relevant SNPs were obtained from the UCSC Genome Browser Gateway (http://genome.ucsc.edu/) using the *Bos taurus* UMD 3.1 of the Tau 6 release. Intervals of 500 kb (250 kb upstream and 250 kb downstream of the significant SNP) were considered in both applied approaches.

## Results

### Detection of significant SNPs

The average amount of variance explained by the four canonical variables (Table 1) ranged from 0.56 for CVA1 on BTA23 to 0.08 for CVA4 on BTA28. An increase in the amount of variance extracted by the first CVA was observed from longer to shorter chromosomes (i.e. 0.44 for BTA1 and 0.54 for BTA29, respectively).

**Table 1:** Variance explained by the four canonical variables for each chromosome

Chromosome	CVA1^a^	CVA2	CVA3	CVA4
BTA1	0.44	0.27	0.17	0.12
BTA2	0.44	0.27	0.17	0.12
BTA3	0.48	0.24	0.18	0.10
BTA4	0.51	0.22	0.16	0.11
BTA5	0.44	0.28	0.16	0.12
BTA6	0.42	0.26	0.18	0.14
BTA7	0.47	0.25	0.17	0.11
BTA8	0.46	0.26	0.17	0.11
BTA9	0.50	0.23	0.17	0.11
BTA10	0.48	0.25	0.17	0.10
BTA11	0.48	0.25	0.17	0.10
BTA12	0.53	0.23	0.15	0.09
BTA13	0.47	0.28	0.16	0.10
BTA14	0.54	0.22	0.16	0.09
BTA15	0.52	0.21	0.17	0.10
BTA16	0.51	0.25	0.15	0.09
BTA17	0.53	0.23	0.15	0.09
BTA18	0.54	0.23	0.15	0.08
BTA19	0.52	0.24	0.15	0.09
BTA20	0.52	0.24	0.15	0.09
BTA21	0.49	0.25	0.16	0.09
BTA22	0.53	0.20	0.19	0.08
BTA23	0.56	0.21	0.15	0.08
BTA24	0.50	0.23	0.16	0.10
BTA25	0.54	0.24	0.15	0.07
BTA26	0.55	0.21	0.16	0.09
BTA27	0.52	0.22	0.17	0.09
BTA28	0.52	0.22	0.18	0.08
BTA29	0.54	0.23	0.14	0.09
Mean	0.50	0.24	0.16	0.10
Standard deviation	0.04	0.02	0.01	0.01
Maximum	0.56	0.28	0.19	0.14
Minimum	0.42	0.20	0.14	0.07

^a^CVA1, CVA2, CVA3, and CVA4 are the first, second, third and fourth extracted canonical variable, respectively

The largest values of the Mahalanobis distance were found for Marchigiana with the other breeds [see Additional file 1 Table S1], especially with the Italian Holstein–Friesian breed for BTA10 to 29. The smallest values were observed between Italian Simmental and Piemontese, again for BTA10 to 29.

The number of SNPs that were identified as significant in the F_ST_ analysis was more than 10 times larger than that in the CDA (864 versus 9108 SNPs, Table 2). However, note that the F_ST_ results were based on the sum of the significant SNPs detected in each of the 10 pairwise comparisons. Moreover, several SNPs were detected in two or more pairwise comparisons, thus increasing the number of SNPs detected with F_ST_. When only one pairwise comparison was considered for F_ST_, the number of detected SNPs was comparable to that obtained in the CDA (Table 2). A total of 332 SNPs were found in both the CDA and in at least one of the 10 F_ST_ comparisons.

**Table 2:** Number of significant SNPs detected by the canonical discriminant analysis (CDA) and the fixation index (F_ST_) approach

Pair-wise F_ST_	F_ST_ analysis	Number of common SNPs between CDA and - F_ST_				
	Number of detected significant SNPs	CVA1^a^	CVA2	CVA3	CVA4	Total
BRW–HOL	833	82	19	11	1	113
BRW–MAR	719	10	43	8	0	61
BRW–SIM	821	13	11	32	0	56
BRW–PIE	749	14	19	19	9	61
HOL–MAR	1035	82	21	8	2	113
HOL–SIM	923	82	11	17	3	113
SIM–MAR	883	20	36	24	2	82
PIE–HOL	1172	97	8	19	11	135
PIE–MAR	943	14	27	10	9	60
PIE–SIM	1030	18	12	19	21	70
Total	9108	432	207	167	58	864
Unique SNP in common		155	78	66	33	332

*BRW* Italian Brown Swiss, *HOL* Italian Holstein, *MAR* Marchigiana, *SIM* Italian Simmental, *PIE* Piemontese cattle

^a^CVA1, CVA2, CVA3, and CVA4 are the first, second, third and fourth extracted canonical variable, respectively

### Detection of signatures of selection

The CDA highlighted a large number (n = 613) of signatures of selection that were characterized by one or more SNPs (their number ranging from 1 to 8). The largest number of signatures of selection was detected on BTA1 (n = 45), whereas the smallest number was on BTA29 (n = 8). BTA4 had the largest number of relevant SNPs that were located in the same region. In particular, eight SNPs between 76.9 and 77.6 Mb were detected for CVA1 and five SNPs (between 34 and 35 Mb) for CVA2.

**Fig. 1:**
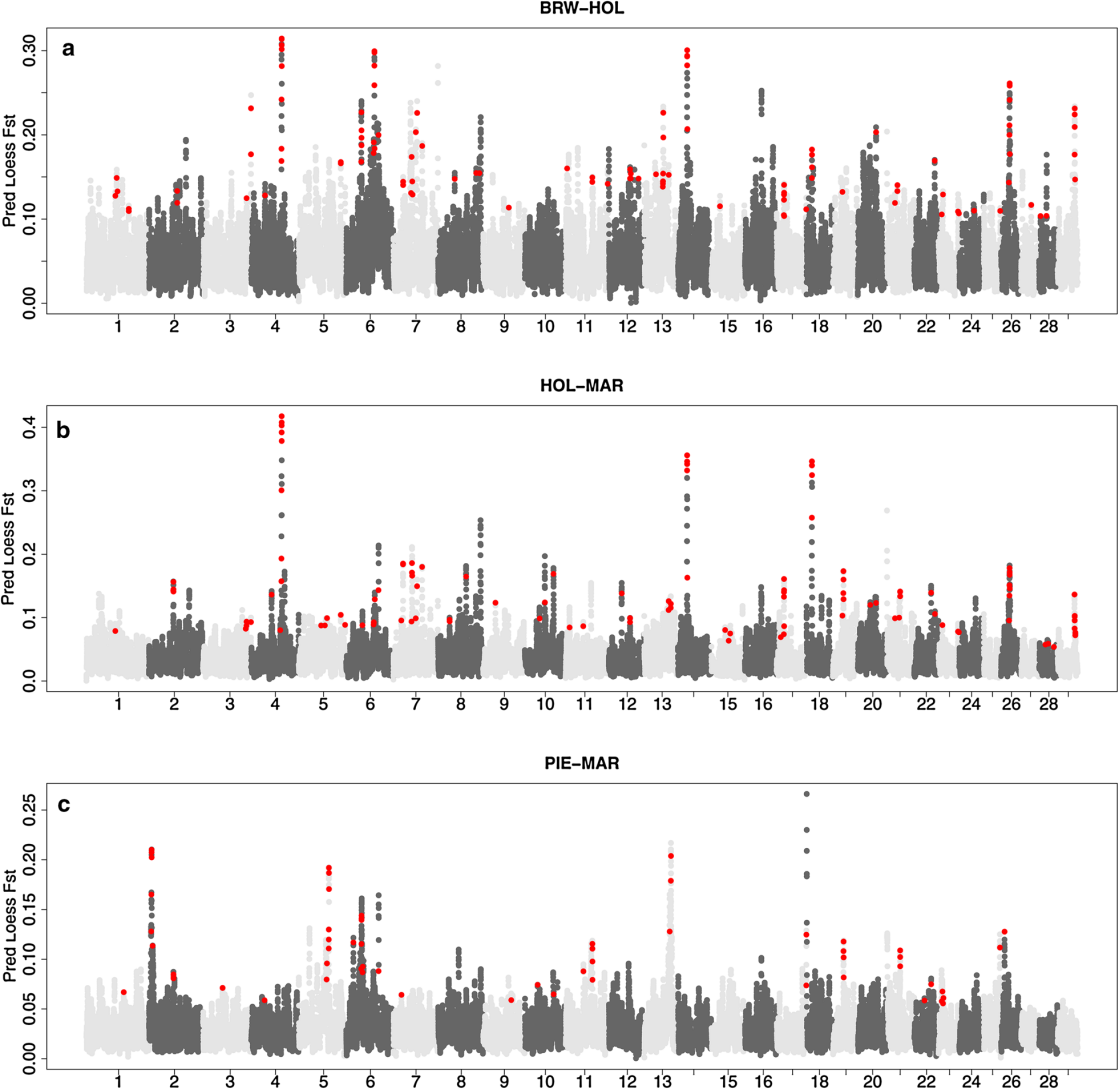
Manhattan plot of F_ST_ (*light and dark grey dots*) and canonical coefficients (*red dots*) for Italian Brown Swiss-Italian Holstein (**a**), Italian Holstein-Marchigiana (**b**), and Piemontese-Marchigiana (**c**) comparisons, respectively

Comparison between the results of the CDA and F_ST_ analyses shows good agreement between the locations of the most relevant signatures of selection [see Additional file 2 Figure S1]. Figure 1a compares the CDA and F_ST_-based results for the two dairy breeds (Italian Holstein–Friesian and Italian Brown Swiss) and shows that both methods identify signatures of selection on BTA4, 6, 7, 14, 26, and 28. Similarly, both methods detected common signatures of selection in the dairy versus beef cattle comparison (Italian Holstein–Friesian and Marchigiana; Fig. 1b) on BTA2, 4, 6, 7, 14, 18, and 26 and in the comparison between the two specialized beef breeds (Piemontese and Marchigiana; Fig. 1c) on BTA2, 5, 6, 13, 18, and 26.

### Detection of signatures of selection that include known genes

**Fig. 2:**
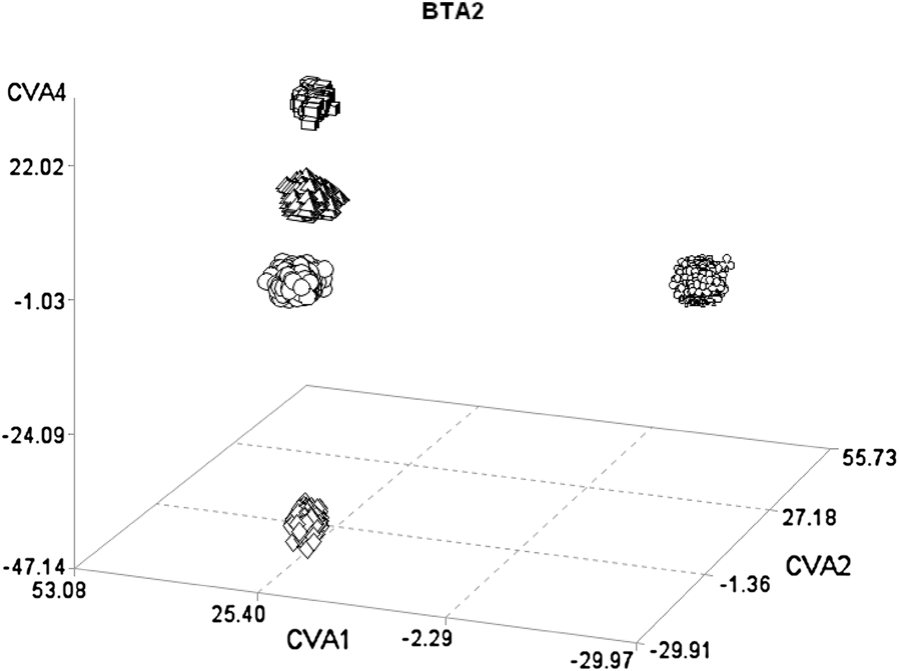
Plot of the individual scores of the first, second and fourth canonical variables (CVA1, CVA2, and CVA4) extracted from BTA2 in the five breeds. *Circles* Italian Brown Swiss; *flowers* Italian Holstein; *diamonds* Piemontese; *cubes* Marchigiana; *pyramids* Italian Simmental

More than 200 candidate genes that have a role in metabolic pathways of interest for the considered breeds were identified in the genomic regions flagged by the CDA [see Additional file 3 Table S2]. These results suggest a good reliability of this method. On BTA2 for example, the CVA4 was able to separate the Piemontese breed from the other breeds (Fig. 2). Among the SNPs that had the largest correlation with this CVA (Table 3), three were located between 6.6 and 6.8 Mb on BTA2, which is where the *myostatin* locus maps. CVA1, with two SNPs positioned between 5.8 and 6.1 Mb, distinctly separated the Italian Holstein- Friesian from the two beef breeds (Fig. 2), while the Italian Brown Swiss was found at an intermediate position between these two breeds. This region contains the *inositol polyphosphate*-*1*-*phosphatase* (*INPP1*) gene. Moreover, the CVA2 that separated the Italian Brown Swiss from the other breeds had six significant SNPs in the region around 62 Mb, which contains the *lactase* (*LCT*) gene.

**Table 3:** SNPs with the 1 % highest correlations with CVA4 for BTA2

Marker	Position in Mb	Correlation with CVA4^a^
Hapmap55212-rs29013415	9,245,063	0.46
Hapmap38411-BTA-48376	9,499,870	0.42
Hapmap32300-BTA-133908	38,980,525	0.42
Hapmap47560-BTA-30470	6,831,955	0.40
ARS-BFGL-NGS-11319	6,763,227	0.40
ARS-BFGL-NGS-112454	6,675,045	0.36
BTB-01977132	7,520,210	0.35
Hapmap51331-BTA-85692	8,272,673	0.354
UA-IFASA-5029	111,206,088	0.349
BTB-01929922	8,188,132	0.339
Hapmap57611-rs29021061	5,464,367	0.338
ARS-BFGL-NGS-28178	58,653,662	0.336
Hapmap44381-BTA-47399	5,640,288	0.327
ARS-BFGL-NGS-106761	5,601,419	0.314
ARS-BFGL-NGS-90839	7,169,804	0.297
ARS-BFGL-NGS-10357	132,764,293	0.293
BTA-47785-no-rs	4,958,110	0.292
Hapmap39337-BTA-46816	4,488,303	0.292
ARS-BFGL-NGS-18261	1,896,078	0.290
BTB-00078691	7,492,224	0.283
ARS-BFGL-NGS-5566	107,378,666	0.282
Hapmap54594-rs29019168	113,899,270	0.281
BTB-00078030	4,421,299	0.280

^a^Fourth extracted canonical variable

**Fig. 3:**
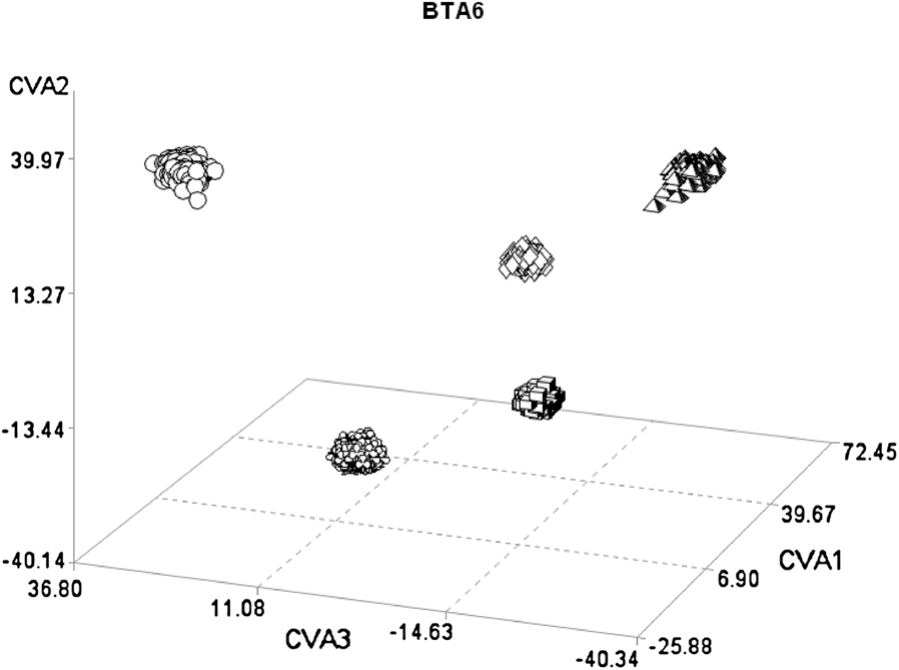
Plot of the individual scores of the first three canonical variables (CVA1, CVA2, and CVA3) extracted from BTA6 in the five breeds. *Circles* Italian Brown Swiss; *flowers* Italian Holstein; *diamonds* Piemontese; *cubes* Marchigiana; *pyramids* Italian Simmental

On BTA6, CVA2 showed that the Marchigiana breed was separated from the other breeds (Fig. 3). The correlation structure of this canonical variable had large correlations with six SNPs that identified two closely-located clusters of genes between 37 and 39 Mb, that are known to affect dairy (*PDK2*, *SPP1*, *MEPE*, and *ABCG2*) and beef (*IBSP*, *LAP3*, *NCAPG*, and *LOCRL*) traits, respectively. On the same chromosome, CVA3 revealed a gradual separation between breeds according to production aptitudes (dairy ⇒ beef ⇒ dual purpose) (Fig. 3). The structure of this CVA (Table 4) showed large correlations with six SNPs that were located between 71.4 and 71.8 Mb, a region that contains the *platelet derived growth factor receptor, alpha polypeptide* (*PDGFRA*) gene.

**Table 4:** SNPs with the 1 % highest correlations with CVA3 for BTA6

Marker	Position in Mb	Correlation with CVA3^a^
Hapmap31616-BTC-042811	71,873,004	0.672
Hapmap42715-BTA-87995	80,128,784	0.640
Hapmap44452-BTA-22099	89,399,736	0.633
Hapmap27692-BTC-042876	71,519,635	0.623
Hapmap56688-rs29025335	81,767,374	0.623
Hapmap33128-BTC-041916	71,421,017	0.622
ARS-BFGL-NGS-38827	71,476,002	0.621
Hapmap32220-BTC-042831	71,552,977	0.612
Hapmap26269-BTC-041695	71,452,210	0.609
BTA-77011-no-rs	82,773,692	0.594
BTB-00272881	97,826,840	0.588
BTA-110240-no-rs	81,652,194	0.583
Hapmap27224-BTA-161106	81,551,479	0.571
Hapmap30962-BTC-032558	33,189,478	0.559
BTA-20903-no-rs	81,467,492	0.549
ARS-BFGL-NGS-67658	105,075,435	0.536
Hapmap52018-BTA-75646	29,355,660	0.530
Hapmap48462-BTA-77136	93,080,797	0.530
BTB-01312468	64,487,002	0.530

^a^Third extracted canonical variable

**Fig. 4:**
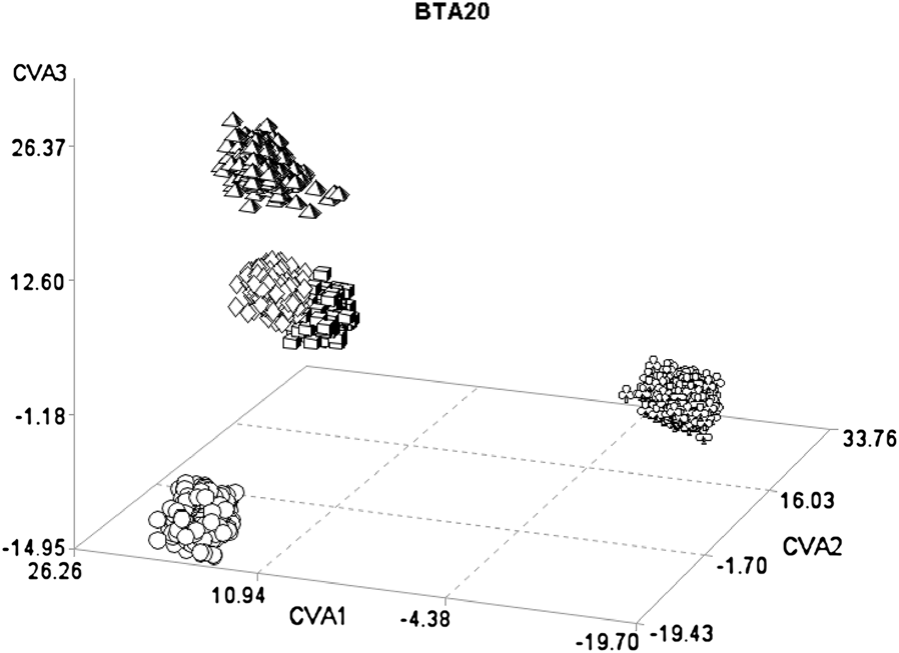
Plot of the individual scores of the first three canonical variables (CVA1, CVA2, and CVA3) extracted from BTA20 in the five breeds. *Circles* Italian Brown Swiss; *flowers* Italian Holstein; *diamonds* Piemontese; *cubes* Marchigiana; *pyramids* Italian Simmental

On BTA20, CVA1 identified a clear separation between Italian Holstein–Friesian and the other breeds (Fig. 4). This CVA1 was correlated with several SNPs that were within the genomic region that contains the *growth hormone receptor* (*GHR*) gene (Table 5).

**Table 5:** Top 1 % highest correlation coefficients between CVA1 and SNP genotypes for BTA20

Marker	Position in Mb	Correlation with CVA1
BTA-50702-no-rs	46,405,056	0.812
BTA-50697-no-rs	46,346,416	0.780
ARS-BFGL-NGS-102895	24,228,836	0.751
Hapmap54326-rs29009836	21,160,226	0.744
Hapmap42401-BTA-102906	39,538,676	0.724
ARS-BFGL-NGS-10108	31,848,979	0.719
BTB-00784875	44,452,488	0.697
Hapmap52341-rs29025776	11,971,234	0.673
ARS-BFGL-NGS-39275	70,454,164	0.665
BTA-113191-no-rs	33,256,096	0.660
Hapmap51681-BTA-110411	18,882,445	0.657
ARS-BFGL-NGS-93510	25,429,674	0.653
BTB-01583562	55,425,112	0.648

Three well-known genes involved in the determinism of coat color in mammals were correlated with CDA: (1) on BTA6, CVA1 was correlated mainly with SNPs that were located close to the *v*-*kit Hardy Zuckerman 4 feline sarcoma viral oncogene homolog* (*KIT*) locus; (2) on BTA18, CVA1 was correlated with three SNPs that were located between 14.3 and 14.5 Mb, where the *melanocortin 1 receptor* (*MC1R*) gene is positioned; and (3) on BTA13, CVA2 separates the Marchigiana breed from the other ones and was mainly correlated with SNPs that mapped close to the *agouti signalling protein* (*ASIP*) gene.

Other known genes that were identified in the discrimination between the five breeds were the *leptin receptor* (*LEPR*) on BTA3, and the *stearoyl*-*CoA deasturase* (*SCD*) on BTA26 and the family of *cathelicidins* (*CATHL*) on BTA22, respectively.

### Detection of signatures of selection that include candidate genes

The CDA analysis also identified several genomic regions that harbored genes, which have not been previously reported in studies on the detection of signatures of selection for cattle breeds. Two large signatures of selection were identified on BTA4 with (1) the *glucokinase* (*hexokinase 4*) (*GCK*), and the *insulin like growth factor binding protein 3* and *1* (*IGFBP1* and *IGFBP3*) located in the first signature of selection (between 77.7 and 77.9 Mb); and (2) the *glutamate receptor metabotropic 3* (*GRM3*) in the second signature of selection. On BTA17, seven SNPs were identified within a region between 18.3 and 19.1 Mb that includes seven annotated genes (*NDUFC1*, *RAB33B*, *CCRN4L*, *MGST2*, *ELF2*, *THOC7*, and *MGARP*). Several signatures of selection in regions that harbor genes involved in the metabolism of milk oligosaccharides (MO) were detected across the genome [see Additional file 3 Table S2] i.e.: *B3GALNT1* on BTA1 (two SNPs for CVA2), *MGAT1* on BTA7 (one SNP for CVA3), *GALNTL1* on BTA10 (one SNP for CVA3), *POMT*, *ST6GAL2* and *GALNT14* on BTA11 (four SNPs for CVA1 and one for CVA2, respectively).

Interestingly, on BTA13, CVA1 emphasized the separation between Italian Holstein–Friesian and Marchigiana [see Additional file 4 Figure S2], and revealed six significant SNPs between 47.1 and 48.3 Mb. Two genes are present in this region: the *prion protein* (*PRNP*) between 47,400,392 and 47,418,507 bp and the *prion protein 2* (dublet) (*PRND*) between 47,444,352 and 47,449,390 bp. We analyzed the polymorphisms at these significant SNPs and found four C/T SNPs (Hapmap53245-rs29026914, BTB-01997512, Hapmap31215-BTA-32775 and BTB-01718516), one G/T SNP (Hapmap39323-BTA-32823) and one A/C SNP (ARS-BFGL-NGS-3711). Allele frequencies at each SNP differed between breeds (Table 6), with frequencies in the Italian Holstein–Friesian breed differing most from those in the other breeds; of particular interest is the frequency difference at the SNP BTB-01718516, which maps within the *PRPN* locus.

**Table 6:** SNP Allele frequencies (%) for the SNP associated with CVA1 for BTA13

Marker	r_(SNP, CVA1)_^a^	Breed	A	C	G	T
BTB-01997512	0.78973	BRW		0.08		0.92
		HOL		0.84		0.16
		MAR		0.12		0.88
		SIM		0.14		0.86
		PIE		0.22		0.78
ARS-BFGL-NGS-3711	0.73262	BRW	0.05	0.95		
		HOL	0.82	0.18		
		MAR	0.15	0.85		
		SIM	0.34	0.66		
		PIE	0.43	0.57		
Hapmap39323-BTA-32823	0.67370	BRW			0.99	0.01
		HOL			0.48	0.52
		MAR			1.00	0.00
		SIM			0.97	0.03
		PIE			0.98	0.02
Hapmap31215-BTA-32775	0.67348	BRW		0.99		0.01
		HOL		0.43		0.57
		MAR		0.97		0.03
		SIM		0.90		0.10
		PIE		0.85		0.15
BTB-01718516	0.65564	BRW		0.98		0.02
		HOL		0.02		0.98
		MAR		0.90		0.10
		SIM		0.40		0.60
		PIE		0.42		0.58
Hapmap53245-rs29026914	0.65456	BRW		0.15		0.85
		HOL		0.80		0.20
		MAR		0.26		0.74
		SIM		0.30		0.70
		PIE		0.39		0.61

*BRW* Italian Brown Swiss, *HOL* Italian Holstein, *MAR* Marchigiana, *SIM* Italian Simmental, *PIE* Piemontese

^a^Correlation between the SNP and the first canonical variable extracted from BTA13 that maps close to *PRNP* and *PRND* genes

Finally, three genes regulated by epigenetic mechanisms were detected. CVA1 for BTA3 was associated with two chromosome-wide significant SNPs located at around 36.5 Mb. The closest gene to these two SNPs is *arginine methyltransferase 6* (*PRMT6*). On BTA21, CVA1 was correlated with a SNP located at 67.4 Mb, close to the *maternally expressed gene 3* (*MEG3*). Finally, CVA3 for BTA28 was significantly associated with a SNP at 24.6 Mb, close to the *sirtuin*, *type 1* (*SIRT1*) gene.

## Discussion

### Canonical discriminant analysis

The use of canonical discriminant analysis on 50 K SNP genotypes allowed the extraction of new variables that were able to maximize the separation between breeds. Interpretation of the canonical structure led to the identification of a large number of signatures of selection. The CDA approach is quite straightforward, based on simple visual inspection of individual locations in the canonical spaces and on interpretation of the canonical structure. Analyses were carried out separately for each chromosome in order to mitigate the large unbalance between the number of animals and the number of SNPs. An alternative would be to select SNPs based on their ability to discriminate among populations. However, the use of selected SNP panels for the discrimination of individuals between breeds may give different results depending on the metric used [38]. Moreover, SNP selection results in loss of information, while all SNPs are represented in each CVA.

A useful feature of CDA compared to other methods for detection of signatures of selection is that more than two groups can be compared in a single calculation step while other approaches may require repeated calculations. For example, in this study 10*39,833 F_ST_ values had to be calculated in order to carry out the comparisons among the five breeds.

**Fig. 5:**
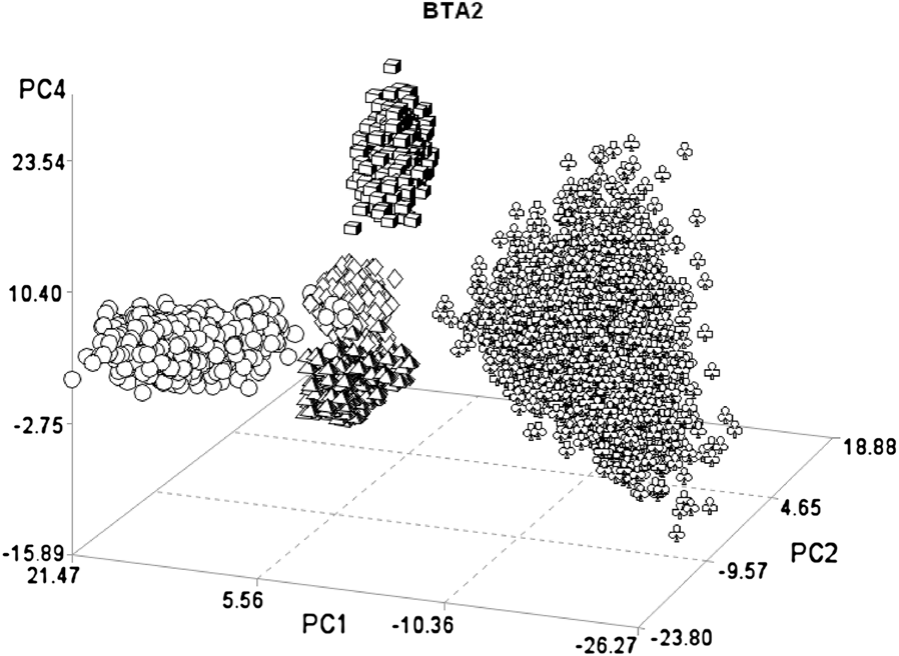
Plot of the individual scores of the first, second and fourth principal components (PC1, PC2, and PC4) extracted from BTA 2 for the five breeds. *Circles* Italian Brown Swiss; *flowers* Italian Holstein; *diamonds* Piemontese; *cubes* Marchigiana; *pyramids* Italian Simmental

In genetic diversity studies, a widely used multivariate method is principal component analysis. Figure 5 shows the plot of individual scores of the first three principal components (PC) extracted from BTA2. The comparison with Fig. 2, which shows the scores of the first three CVA extracted from the same chromosome, reveals larger within-breed variability in the PCA plot. These results are a consequence of theoretical differences between these two multivariate techniques [30]. PCA extraction is aimed at accounting for progressive descending amounts of the original variance (which includes both between- and within-group variation) without any assumption on sample stratification. In contrast, CVA extraction is aimed at maximizing variation between predefined groups. The greater ability of CVA to discriminate between groups is also enhanced by a different partition of the variance across the new variables. The number of PC is equal to the number of original variables, whereas the number of CVA is equal to the number of groups minus 1. In the example of BTA2, the first three canonical variables explained 88 % of the variance (Table 1) whereas the first three PC accounted for 13 % of the variance (8, 3, and 2 % respectively).

### Detection of signatures of selection

A large number of signatures of selection were detected and well-known or new candidate genes were identified. This result could be, at least partially, due to the structure of the sample analysed. Breeds with different production aptitudes and selection histories were considered. As a result, genes that are involved in specific biological functions or metabolic pathways of interest were detected [see Additional file 3 Table S2]. These results are consistent with those from previous studies [12, 14, 24, 39] and with our results obtained by using the popular F_ST_ approach. However, several of the identified regions did not contain genes, either because annotation of the bovine genome is incomplete or the SNP was positioned outside a coding region [14, 40]. In any case, compared to other studies that were based on SNP or whole-genome sequence data, the number of signatures of selection that we detected was relatively large. Moreover, the number of detected regions was larger than that previously obtained using the same data [41], or different methods [24, 40, 42]. Overall, the comparison of the results between CDA and F_ST_ analyses revealed 290 genes that were detected by both methods [see Additional file 5 Table S3].

Strong signatures of selection were observed on BTA13. Two adjacent genes (*PRPN* and *PRND*) are located within the peak at 47–48 Mb. The *PRPN* and *PRND* genes encode the prion protein (PrP) and the doppel protein (Dpl), respectively. PrP is a transmembrane glycoprotein whose normal function is still unknown [43]. It is widely known that the endogenous PrP is responsible for the occurrence of transmissible spongiform encephalopathies (TSE) [44]. In domestic and wild animals, several distinct TSE diseases are recognized. Among these, the best known are scrapie in sheep and goat, and the bovine spongiform encephalopathy (BSE) in cattle [45]. SNPs and indel events are responsible for the genetic polymorphism at the *PRPN* locus [46], with the SNPs being responsible for atypical forms of BSE, while indels appear to be involved in susceptibility to disease [44, 47]. However, the high degree of conservation of the *PRPN* gene and its ubiquitous expression among mammals suggest several biological roles, such as regulation of the circadian rhythm, central nervous system development, neuronal survival, and maintenance of cellular Ca^+2^ homeostasis [48]. In cattle, several studies recently revealed a large allelic variability at the *PRPN* and *PRND* loci [49–51] and also associations with susceptibility to BSE [52–54]. A recent investigation on the possible association between polymorphisms of the *PRPN* gene and milk production traits in cattle led to statistically significant results for milk fat content [55]. Using the same data as in our study but with a different approach, Mancini et al. [41] detected a signature of selection at the same position on BTA13 and identified the *CDP*-*diacylglycerol synthase* (*CDS2*) gene, which is involved in the phospholipid biosynthetic process. This signature of selection may be an effect of artificial selection but the signal that we detected here, in the region that contains the *PRPN* and *PRND* genes, could be the result of natural selection. However, more studies are necessary on e.g. the distribution and frequency of genetic variants, linkage between *PRPN* alleles, recombination rate, and haplotype diversity within and between populations, in order to improve management of the disease (especially atypical cases) and possibly production performance.

### New candidate genes

A new result of interest in our study was the detection of signatures of selection in regions that harbor genes involved in the metabolism of bovine colostrum/milk oligosaccharides (MO). The first biochemical characterization of these molecules was done in the early 1980s [56]. Recently, several studies have been conducted to clarify the biological role of MO in mammals [57–59]. In mammalian milk, a large variability in concentration, composition and timing of oligosaccharides is observed during lactation. Changes in the quality and quantity of MO do not depend on the nutritional status of the mother [60]. Variations in glycans may be genetically driven but may also result from response to environmental pressures [61]. From an evolutionary point of view, this variability is explained by greater fitness [62]. In fact, presence of free oligosaccharides in the milk must have a selective advantage (for the mammary gland and for the offspring) [63]. These molecules do not play a nutritional role (although they are carbohydrates) since they pass undigested into the large intestine [60]. However, several studies have shown that MO play a critical role in development and maintenance of the intestinal bacterial flora and protection against enteric diseases [64–66]. The role of MO in human health [61, 67] and the genes involved in their metabolism have been investigated [68]. Currently, because of their role as micronutrients and prebiotics, there is much interest in elucidating their genetic basis in mammalian species [56]. Genes that are involved in the metabolism of MO in cattle were recently reported [69] and were consistent with about one third of those identified in our study. Bovine milk was studied as a possible source of functional oligosaccharides for improving human health [70].

### Genes involved in epigenetic regulatory mechanisms

In our genome-wide survey, three SNPs defined three signatures of selection on BTA3, 21 and 28, respectively. Among the identified genes, *PRMT6*, *MEG3* and *SIRT1* are under epigenetic control and represent interesting candidate genes. Traditionally, for traits of economic interest, the relationship between genome and phenome has been investigated by considering that variability was the result of several combined genetic and environmental factors. Until a few years ago, epigenetics was neglected in livestock production. However, over the last 20 years, there have been numerous articles on this topic in humans, mouse and plants [71, 72]. Only recently, QTL that affect productive performance have been considered to be subject to epigenetic mechanisms [73]. Differences in the epigenome may explain some of the phenotypic variations observed within populations. Economically important traits such as milk composition and yield or muscle mass and fat deposition appear to be the result of a synergy between the genome and epigenome [74–76].

*MEG3* on BTA21 was previously reported to be polymorphic in cattle but the polymorphism was not associated with production traits [75]. In beef breeds, CDA (CVA3) identified a strong signal on BTA28 where the *sirtuin1* (*SIRT1*) gene is located. SIRT1 is a nicotinamide adenine dinucleotide (NAD)-dependent deacetylase that is involved in a plethora of biological processes, including metabolic regulation, aging and stress response [76, 77]. In humans, this gene has attracted much interest because polymorphisms at this gene have been associated with longevity [78] and obesity [79] and recently, a polymorphism at this locus was also associated with growth traits in the Nanyang cattle breed [80]. Based on these results, it is clear that it is important to elucidate, at the molecular level, the epigenetic mechanisms that control genes and to understand how they can influence production traits to improve animal production performance.

### The issue of ascertainment bias

In this work, five breeds of different geographical origin, selection histories, morphological appearance and production aptitudes were compared. A main issue of the between-breed comparisons that were carried out is the ascertainment bias caused by the method used to identify the SNPs. On the one hand, although SNP editing in the present work was performed across breeds, it should be recalled that the beadchip used was developed for other ascertainment groups [81]. On the other hand, studies on the effect of ascertainment bias on estimation of genetic diversity parameters (such as F_ST_ or PCA) have led to conflicting results [82, 83]. In any case, none of the studies on genetic variability have considered methods to correct for ascertainment bias and its effect is, at present, not predictable [84]. The main reason of the lack of correction for ascertainment bias is that breed-specific SNP panels are not commercially available. Finally, it should be noted that the BovineSNP50 assay was tested on a panel of 21 indicine and taurine breeds, for which nearly 95 % of the considered SNPs were polymorphic [29], although these authors pointed out that the power of the assay for genome-wide association studies differed between populations.

## Conclusions

Our results suggest that canonical discriminant analysis can be a valid tool for detection of signatures of selection based on 50 K SNP beadchip data. The approach is quite straightforward, allows the comparison of more than two groups at the same time, and relative distances between breeds can be visually appreciated. A large number of signatures of selection were detected, within which, several well known candidate genes that affect meat or milk production traits were identified e.g. *myostatin* and *GHR*. Moreover, several interesting new candidate genes were identified, such as those involved with metabolism of milk oligosaccharides or those known to be regulated by epigenetic mechanisms.

## Additional Files

10.1186/s12711-016-0236-7 Matrices of Mahalanobis distances between the five breeds on the 29 autosomes. This table reports the Mahalanobis distance between the centroids of the five cattle populations calculated with the canonical discriminant analysis. The distances are reported for all the 29 autosomes. BRW = Italian Brown Swiss; HOL = Italian Holstein; MAR = Marchigiana; PIE = Piemontese; SIM = Italian Simmental.
10.1186/s12711-016-0236-7 Manhattan plot of F_ST_ values (light and dark grey dots) and canonical coefficients (red dots) for all ten pairwise comparisons. This plot reports the F_ST_ values and the canonical coefficient along the whole genome obtained in the ten pairwise comparisons between all the five breeds considered in this study, evidencing the concordance between the two approaches in the detection of selection signatures. BRW = Italian Brown Swiss; HOL = Italian Holstein; MAR = Marchigiana; PIE = Piemontese; ISIM = Italian Simmental.
10.1186/s12711-016-0236-7 List of putative candidate genes detected using CDA derived from *Bos taurus* UMD 3.1/bosTau6 assembly. This table reports the genes that have been identified considering an interval of 0.5 Mb around SNPs that have the highest (top 1 %) correlations with the canonical variables.
10.1186/s12711-016-0236-7 Plot of the individual scores of the first three canonical variables (CVA1, CVA2, and CVA3) extracted from BTA13 in the five breeds. This plot represents the clear separation between Italian Holstein and Marchigiana obtained on BTA13; circles = Italian Brown Swiss; flowers = Italian Holstein; diamonds = Piemontese; cubes = Marchigiana; pyramids = Italian Simmental.
10.1186/s12711-016-0236-7 List of markers and genes identified by both CDA and F_ST_ approaches. This table reports the markers and the genes in common between the two considered approaches for detection of selection signatures.
