# Supplementary material for: Use of canonical discriminant analysis to study signatures of selection in cattle
Source: Genet Sel Evol. 2016 Aug 12;48:58. doi: 10.1186/s12711-016-0236-7 (PMC4983034; doi:10.1186/s12711-016-0236-7)
Supplement: Supplementary file 1 — 10.1186/s12711-016-0236-7 Matrices of Mahalanobis distances between the five breeds on the 29 autosomes. This table reports the Mahalanobis distance between the centroids of the five cattle populations calculated with the canonical discriminant analysis. The distances are reported for all the 29 autosomes. BRW = Italian Brown Swiss; HOL = Italian Holstein; MAR = Marchigiana; PIE = Piemontese; SIM = Italian Simmental. [file 12711_2016_236_MOESM1_ESM.pdf]

S1 Table. Matrices of Mahalanobis distances between the five breeds in the 29 autosomes (BRW= Italian Brown Swiss; HOL=Italian Holstein; MAR = MAR; PIE= PIE; SIM=Italian Simmental .

		BRW	HOL	MAR	SIM	PIE
BTA1	BRW	0	8079	16067	8680	8646
	HOL		0	17320	7935	10244
	MAR			0	17480	16090
	SIM				0	8989
	PIE					0
BTA2		BRW	HOL	MAR	SIM	PIE
	BRW	0	4107	7710	4328	4308
	HOL		0	8183	4844	5107
	MAR			0	8345	7291
	SIM				0	4566
	PIE					0
BTA3		BRW	HOL	MAR	SIM	PIE
	BRW	0	4255	6003	3994	3787
	HOL		0	6731	4127	4457
	MAR			0	6295	5445
	SIM				0	3530
	PIE					0
BTA4		BRW	HOL	MAR	SIM	PIE
	BRW	0	3294	4679	3015	3031
	HOL		0	6005	3656	4000
	MAR			0	5094	4682
	SIM				0	3042
	PIE					0
BTA5		BRW	HOL	MAR	SIM	PIE
	BRW	0	3714	9017	5127	5374
	HOL		0	9302	5170	5400
	MAR			0	10271	8230
	SIM				0	6033
	PIE					0
BTA6		BRW	HOL	MAR	SIM	PIE
	BRW	0	3714	9017	5127	5374
	HOL		0	9302	5170	5400
	MAR			0	10271	8230
	SIM				0	6033
	PIE					0
BTA7		BRW	HOL	MAR	SIM	PIE
	BRW	0	2921	4660	2776	2952
	HOL		0	5022	2911	3420
	MAR			0	4730	3823
	SIM				0	2889
	PIE					0

BTA8		BRW	HOL	MAR	SIM	PIE
	BRW	0	2719	5067	2731	2828
	HOL		0	5672	2775	3289
	MAR			0	5679	4669
	SIM				0	**2861**
	PIE					0
BTA9		BRW	HOL	MAR	SIM	PIE
	BRW	0	2083	2844	1908	1775
	HOL		0	3316	2243	2325
	MAR			0	3016	2535
	SIM				0	1890
	PIE					0
BTA10		BRW	HOL	MAR	SIM	PIE
	BRW	0	2277	3709	2192	2059
	HOL		0	4200	2374	2601
	MAR			0	3566	3408
	SIM				0	1990
	PIE					0
BTA11		BRW	HOL	MAR	SIM	PIE
	BRW	0	3062	4310	2774	2683
	HOL		0	4668	2782	3012
	MAR			0	4442	3838
	SIM				0	2400
	PIE					0
BTA12		BRW	HOL	MAR	SIM	PIE
	BRW	0	1531	1883	1089	1145
	HOL		0	2181	1502	1446
	MAR			0	1931	1607
	SIM				0	1047
	PIE					0
BTA13		BRW	HOL	MAR	SIM	PIE
	BRW	0	1956	3170	1731	1853
	HOL		0	3320	1508	1853
	MAR			0	2848	788
	SIM				0	1403
	PIE					0
BTA14		BRW	HOL	MAR	SIM	PIE
	BRW	0	1696	2111	1363	1299
	HOL		0	2732	1773	1712
	MAR			0	2268	1823
	SIM				0	1203
	PIE					0

BTA15		BRW	HOL	MAR	SIM	PIE
	BRW	0	1579	1794	1226	1206
	HOL		0	2126	1554	1512
	MAR			0	1903	1781
	SIM				0	1078
	PIE					0
BTA16		BRW	HOL	MAR	SIM	PIE
	BRW	0	1484	2037	1181	1113
	HOL		0	2185	1328	1478
	MAR			0	1829	1659
	SIM				0	1024
	PIE					0
BTA17		BRW	HOL	MAR	SIM	PIE
	BRW	0	1545	1862	1149	1120
	HOL		0	1934	1538	1448
	MAR			0	1800	1477
	SIM				0	1006
	PIE					0
BTA18		BRW	HOL	MAR	SIM	PIE
	BRW	0	1208	1726	977.84763	832.10716
	HOL		0	2190	1302	1258
	MAR			0	1817	1331
	SIM				0	872
	PIE					0
BTA19		BRW	HOL	MAR	SIM	PIE
	BRW	0	1254	1610	1000	977
	HOL		0	1824	1178	1200
	MAR			0	1380	1324
	SIM				0	803
	PIE					0
BTA20		BRW	HOL	MAR	SIM	PIE
	BRW	0	1609	2197	1266	1217
	HOL		0	2555	1696	1709
	MAR			0	2133	1827
	SIM				0	1205
	PIE					0
BTA21		BRW	HOL	MAR	SIM	PIE
	BRW	0	1181	1566	960	975
	HOL		0	1717	901	1006
	MAR			0	1582	1321
	SIM				0	777
	PIE					0

		BRW	HOL	MAR	SIM	PIE
BTA22		BRW	HOL	MAR	SIM	PIE
	BRW	0	1090	1111	968	747
	HOL		0	1308	1010	955
	MAR			0	1251	945
	SIM				0	691
	PIE					0
BTA23						
	BRW	0	875	866	652	598
	HOL		0	1035	855	747
	MAR			0	804	719
	SIM				0	489
	PIE					0
BTA24		BRW	HOL	MAR	SIM	PIE
	BRW	0	931	1154	716	759
	HOL		0	1274	873	922
	MAR			0	1229	1044
	SIM				0	674
	PIE					0
BTA25		BRW	HOL	MAR	SIM	PIE
	BRW	0	760	916	512	485
	HOL		0	1063	691	674
	MAR			0	935	751
	SIM				0	403
	PIE					0
BTA26		BRW	HOL	MAR	SIM	PIE
	BRW	0	912	997	702	655
	HOL		0	1225	931	760
	MAR			0	993	803
	SIM				0	612
	PIE					0
BTA27		BRW	HOL	MAR	SIM	PIE
	BRW	0	682	752	556	454
	HOL		0	821	662	600
	MAR			0	827	632
	SIM				0	513
	PIE					0
BTA28		BRW	HOL	MAR	SIM	PIE
	BRW	0	628	677	512	456
	HOL		0	696	591	514
	MAR			0	702	511
	SIM				0	382
	PIE					0
BTA29		BRW	HOL	MAR	SIM	PIE
	BRW	0	817	917	566	605
	HOL		0	1002	728	738
	MAR			0	823	662
	SIM				0	517
	PIE					0
