# Supplementary figures and images for: Use of canonical discriminant analysis to study signatures of selection in cattle
Source: Genet Sel Evol. 2016 Aug 12;48:58. doi: 10.1186/s12711-016-0236-7 (PMC4983034; doi:10.1186/s12711-016-0236-7)

# BRW-HOL

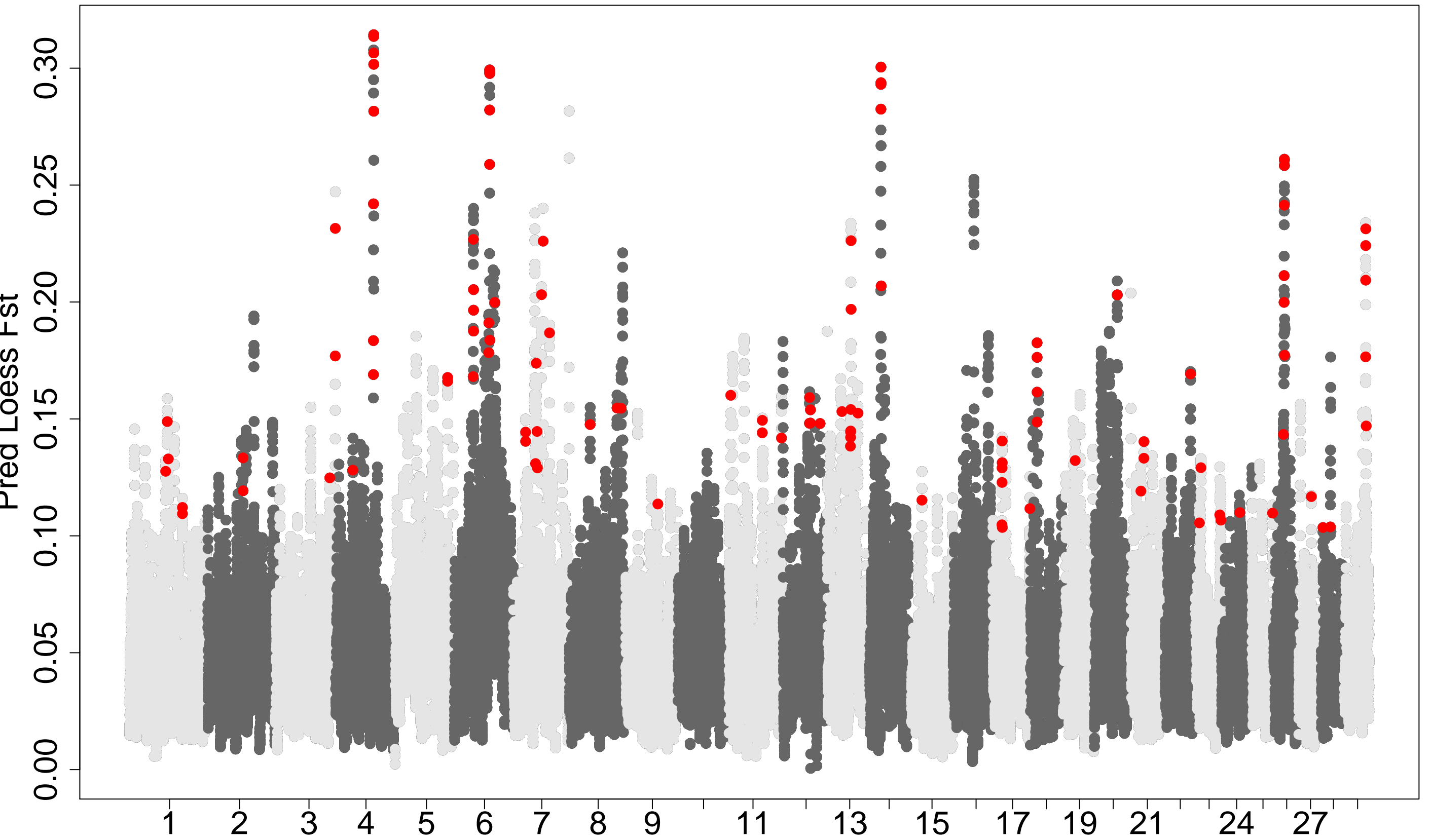

# BRW-MAR

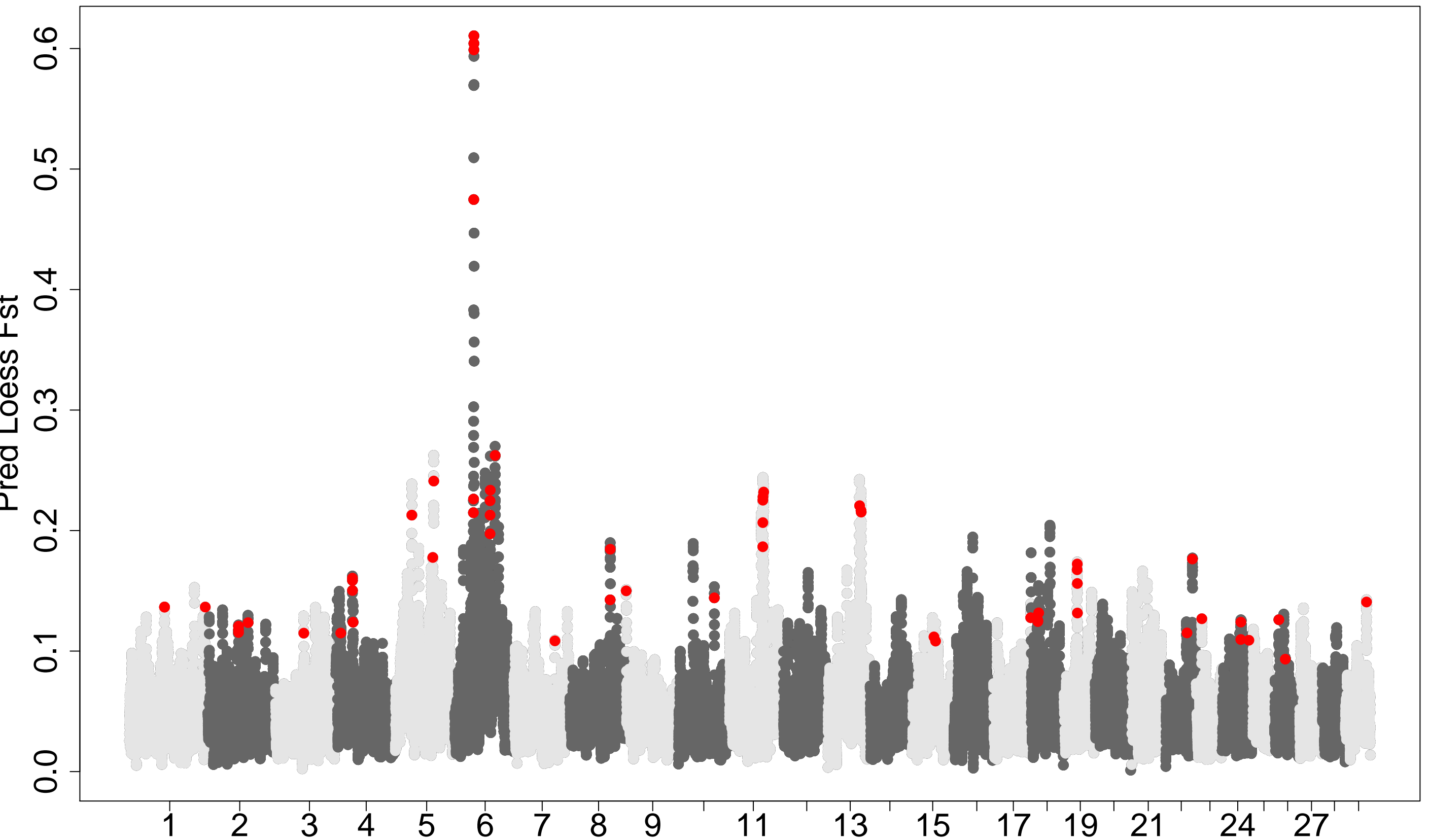

# BRW-SIM

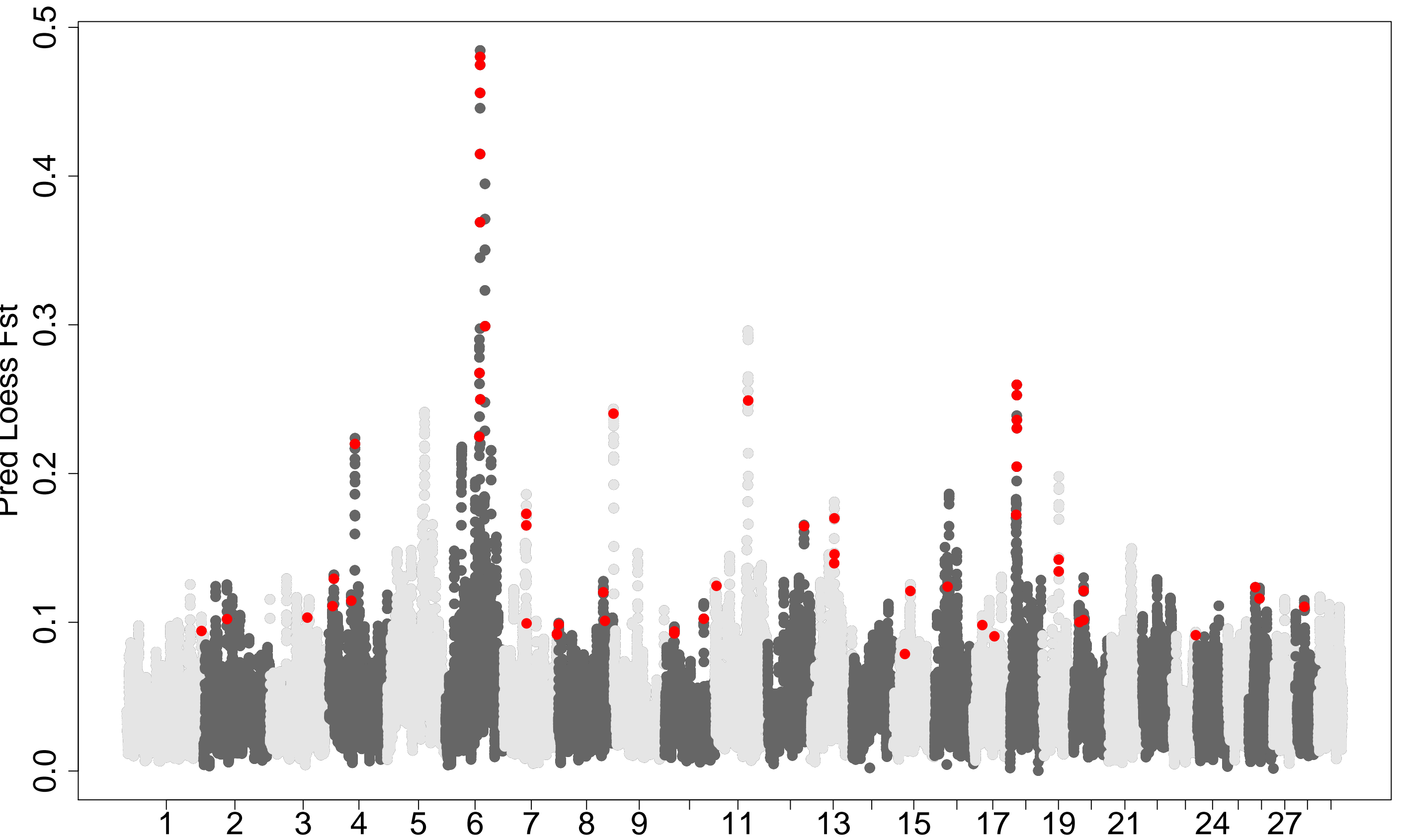

# BRW-PIE

Pred Loess Fst

0.30  
0.25  
0.20  
0.15  
0.10  
0.05  
0.00

1

2

3

4

5

6

7

8

9

11

13

15

17

19

21

24

27

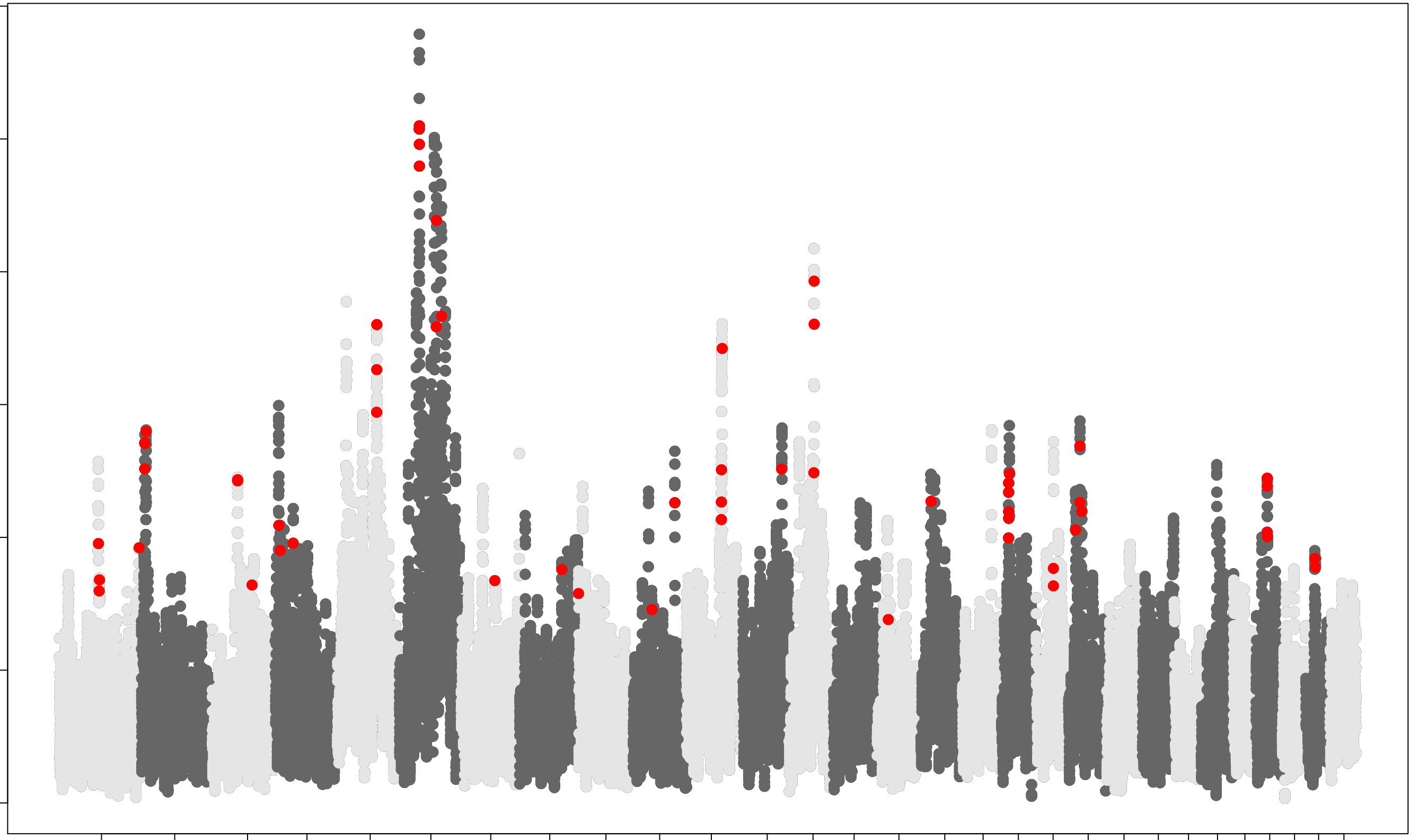

# HOL-MAR

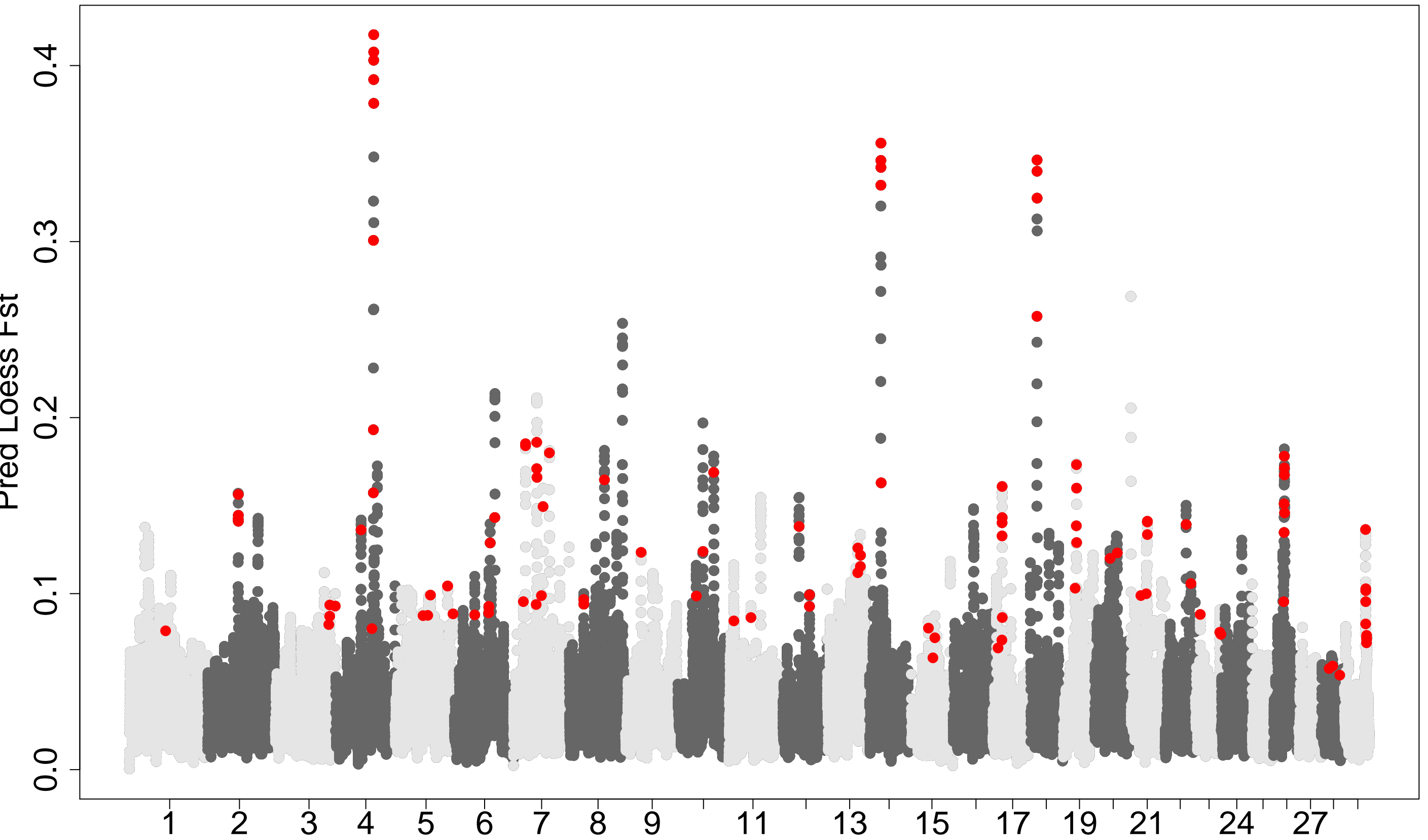

# HOL-SIM

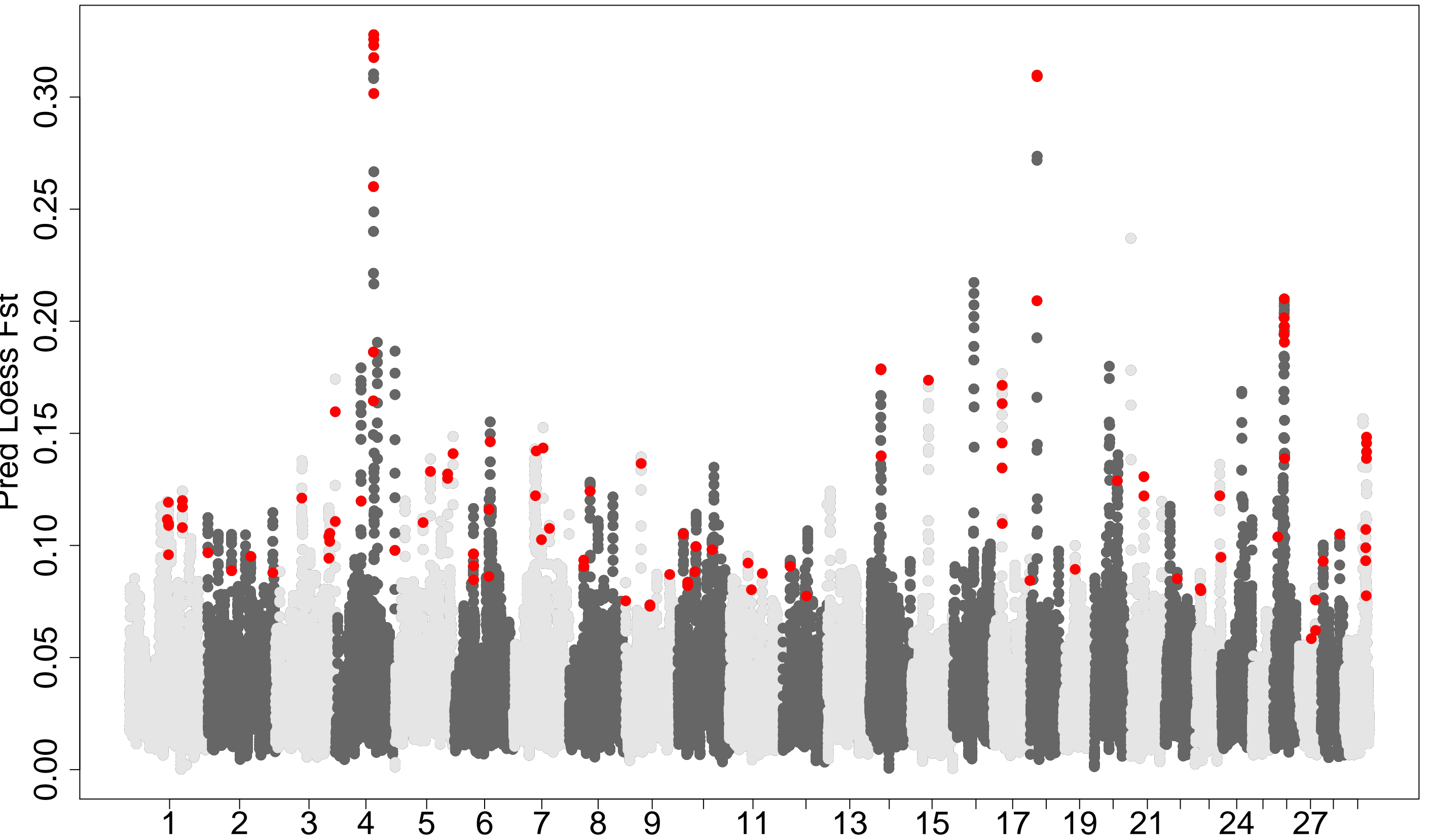

# SIM-MAR

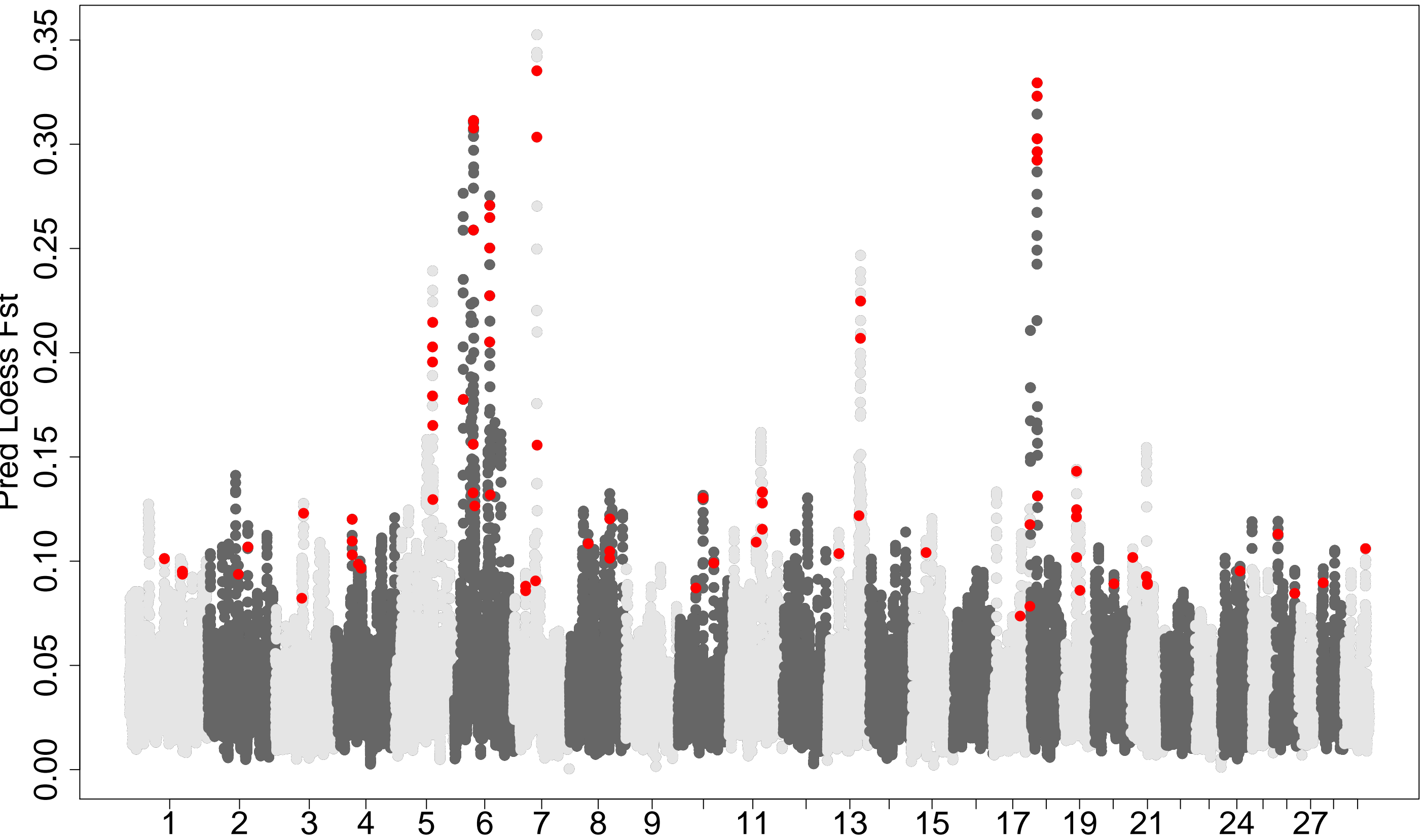

PIE-HOL

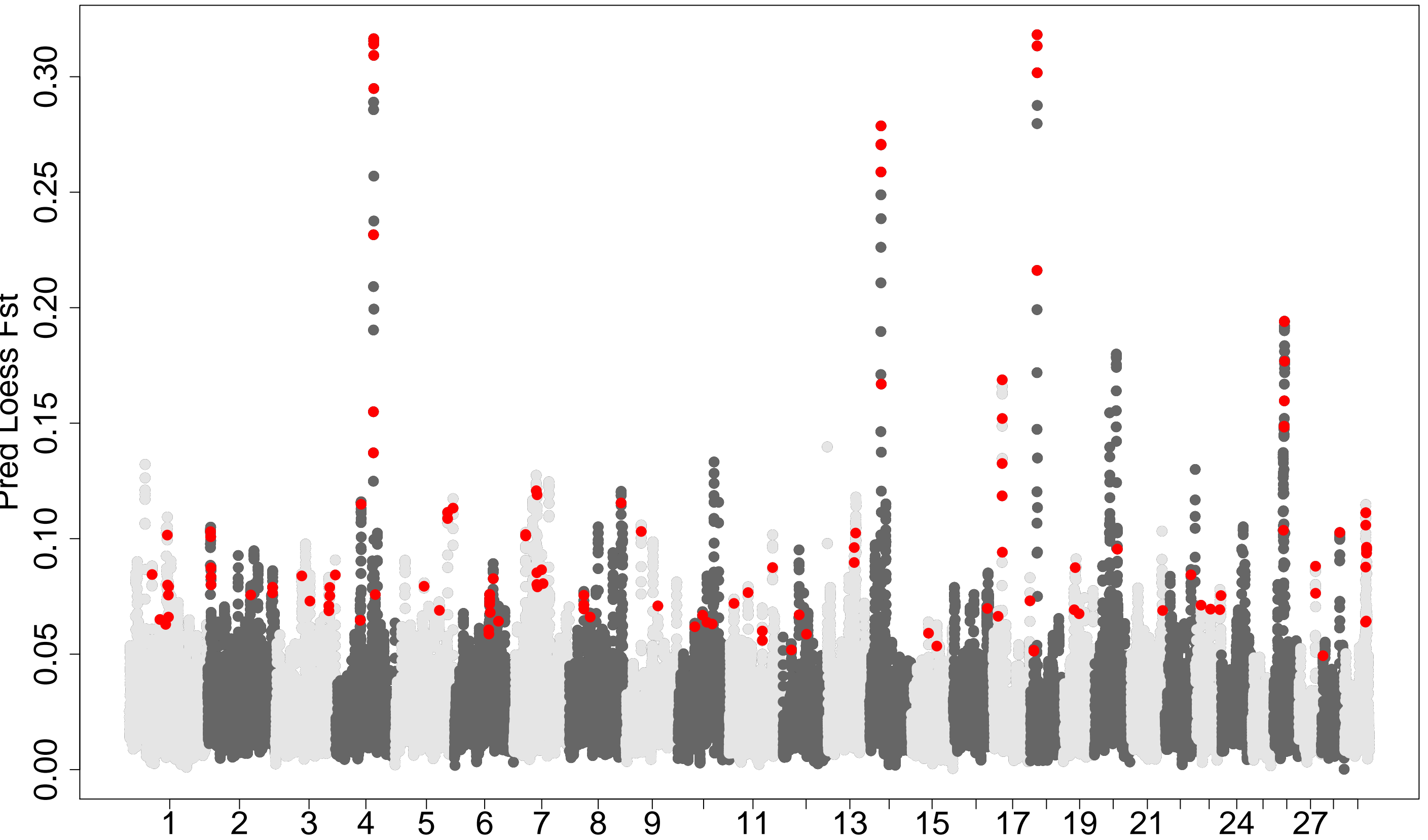

# PIE-MAR

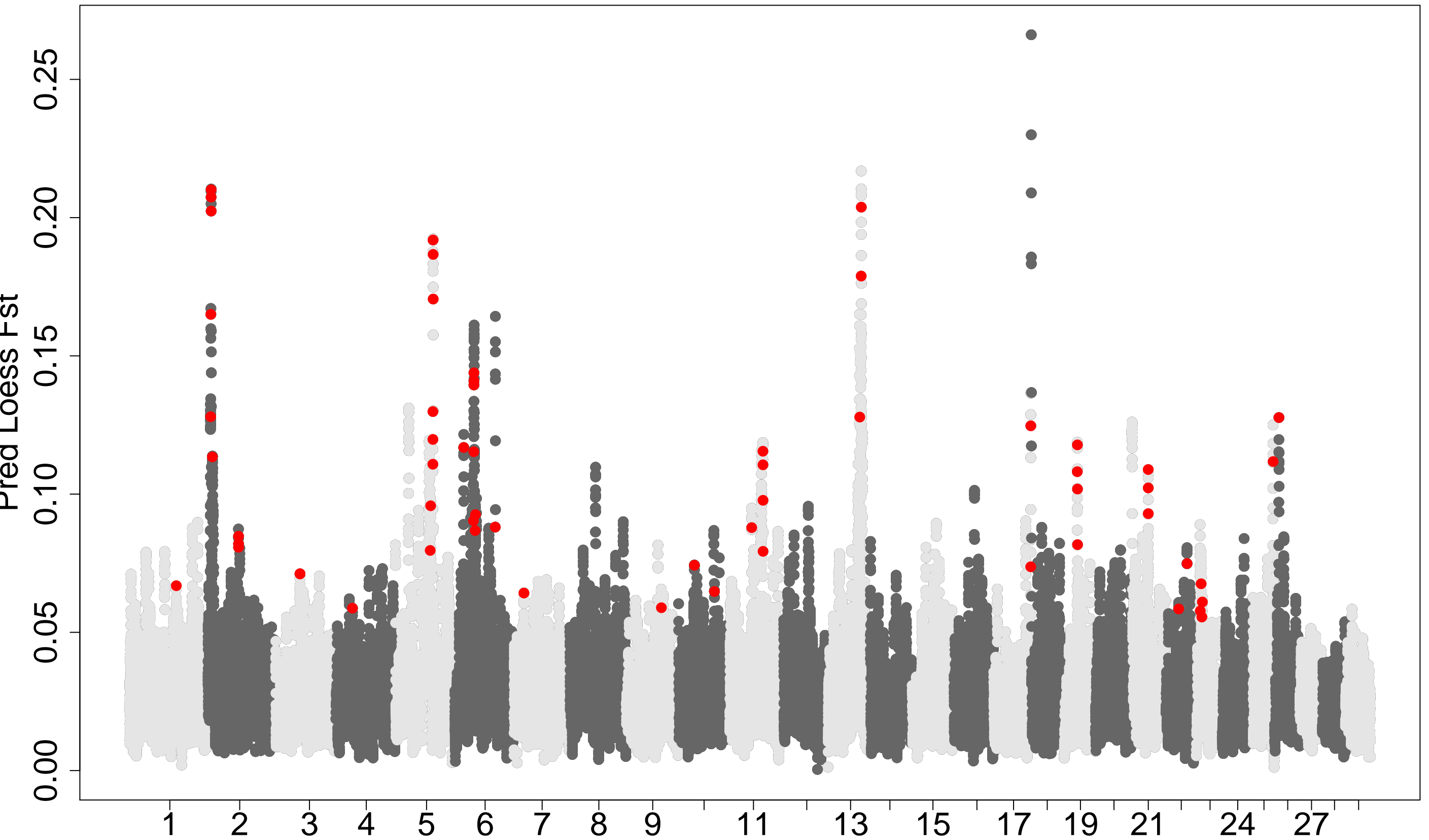

# PIE-SIM

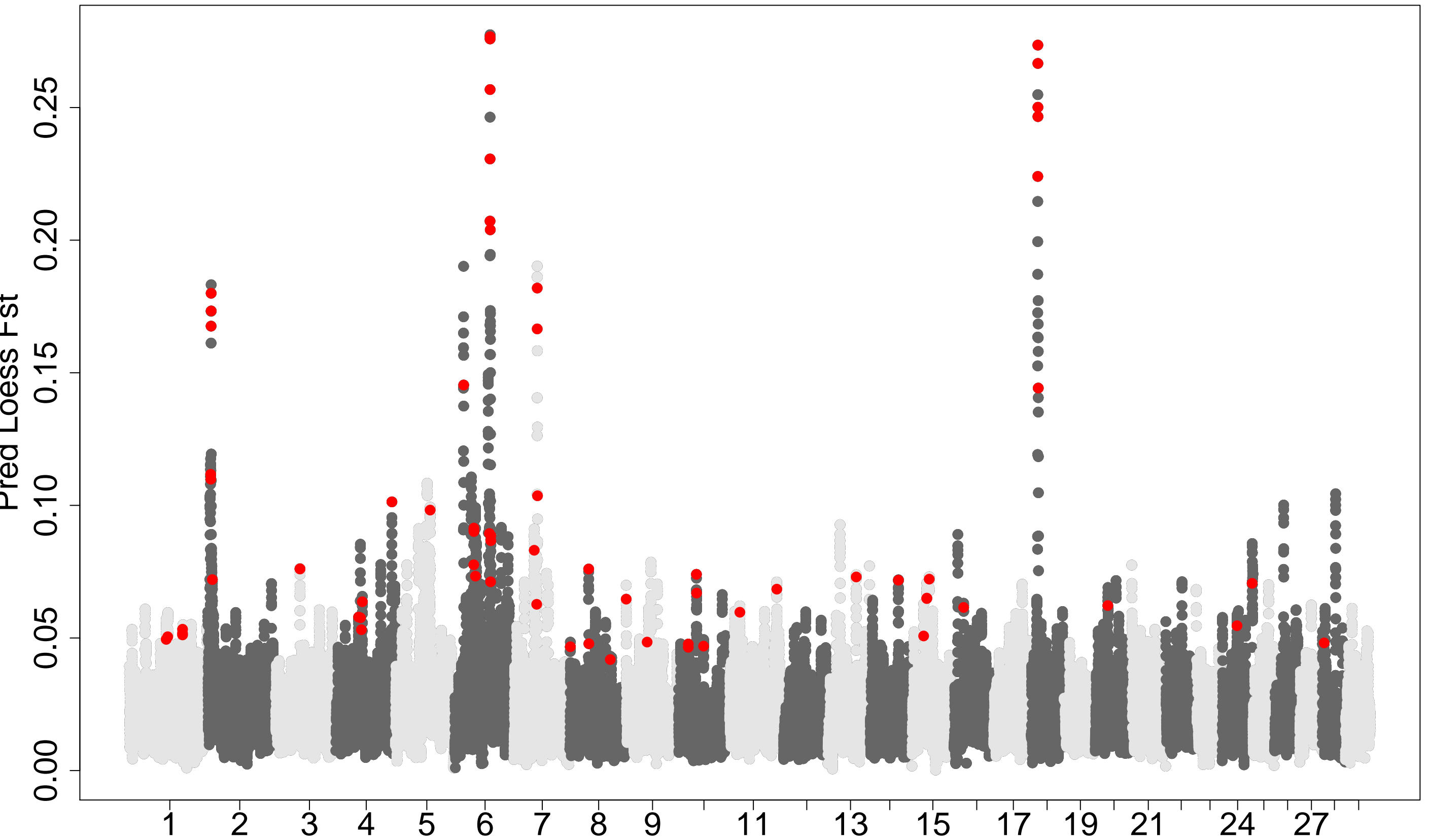

Supplement: Supplementary file 2 — 10.1186/s12711-016-0236-7 Manhattan plot of FST values (light and dark grey dots) and canonical coefficients (red dots) for all ten pairwise comparisons. This plot reports the FST values and the canonical coefficient along the whole genome obtained in the ten pairwise comparisons between all the five breeds considered in this study, evidencing the concordance between the two approaches in the detection of selection signatures. BRW = Italian Brown Swiss; HOL = Italian Holstein; MAR = Marchigiana; PIE = Piemontese; ISIM = Italian Simmental. [file 12711_2016_236_MOESM2_ESM.pdf]

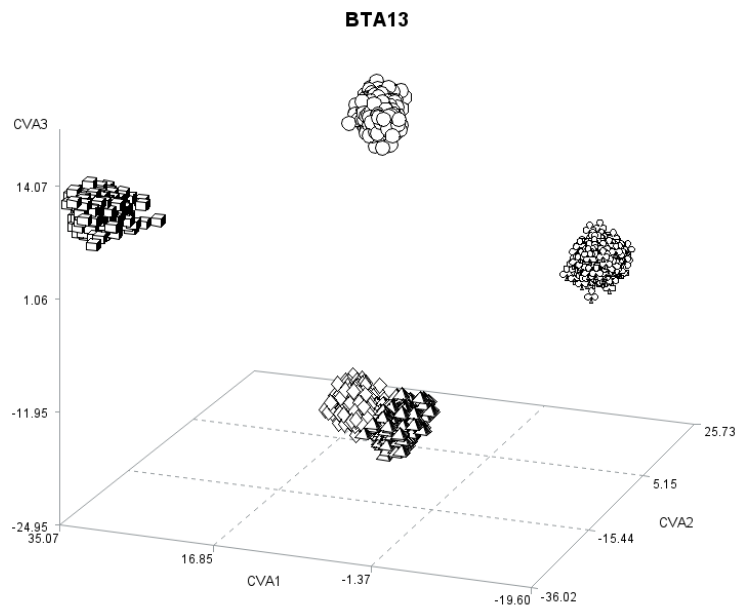

S2 Figure

Supplement: Supplementary file 4 — 10.1186/s12711-016-0236-7 Plot of the individual scores of the first three canonical variables (CVA1, CVA2, and CVA3) extracted from BTA13 in the five breeds. This plot represents the clear separation between Italian Holstein and Marchigiana obtained on BTA13; circles = Italian Brown Swiss; flowers = Italian Holstein; diamonds = Piemontese; cubes = Marchigiana; pyramids = Italian Simmental. [file 12711_2016_236_MOESM4_ESM.pdf]
