# Supplementary material for: Use of canonical discriminant analysis to study signatures of selection in cattle
Source: Genet Sel Evol. 2016 Aug 12;48:58. doi: 10.1186/s12711-016-0236-7 (PMC4983034; doi:10.1186/s12711-016-0236-7)
Supplement: Supplementary file 3 — 10.1186/s12711-016-0236-7 List of putative candidate genes detected using CDA derived from Bos taurus UMD 3.1/bosTau6 assembly. This table reports the genes that have been identified considering an interval of 0.5 Mb around SNPs that have the highest (top 1 %) correlations with the canonical variables. [file 12711_2016_236_MOESM3_ESM.pdf]

S2 Table. List of putative candidate genes derived from the UMD 3.1 assembly involved in several biological processes in mammals

Biological Function	BTA	Position Mb (UMD3.1)	Gene Name
**Immune response and inflammatory processes**	2	135.863.956-135.864.707	**PADI4:** peptidyl arginine deiminase, type IV
	5	75.641.217-75.659. 771	**NCF4:** Neutophil cytosolic factor 4, 40 kDa
	8	23.287.273-23.287.909<br>23.207.723-23.208.355<br>77.394.837-77.396.746<br>77.421.388-77.422.534	**IL6:** Interleukin 6 (interferon beta 2)<br>**IFNB1:** interferon, beta 1, fibroblast<br>**CCL19:** chemokine (c-c motif) ligand 19,<br>**CCL21:** chemokine (c-c motif) ligand 21
	14	2.814.589-2.816.686	**LYN:** V-Yes Yamaguchi Sarcoma Viral related oncogene
	15	22.800.425-22.826.666	**IL18:** Interleukin 18 (interferon gamma inducing factor)
	16	36.976.802-36.979.964	**XCL1:** Chemokine (C motif) ligand 1
	17	18.331.272-18.369.333	**MGST2:** microsomal glutathione S-transferase 2
	18	1.548.806-1.608.706	**IL34:** Interleukin 34
	20	24.048.537-24.059.238<br>24.096.931-24.107.687<br>33.328.558-33.405.557	**GZMA:** Granzyme A (granzyme 1 citotoxic T- linphocyte assiociated serine esterase 3)<br>**GZMK:** Granzyme K (granzyme 3; tryptase II)<br>**C6:** complement component 6
	22	52.148.151-52.219.751	**CATHL1**; **CATHL2**; **CATHL3**; **CATHL5**; **CATHL6**: cathelicidin antimicrobila peptides
	25	26.910.552-26.960.485<br>26.765.639-26.769.345	**ITGAL:** integrin, alpha L (antigen CD11A (P180), lymphocyte function associated antigen 1, alpha polypetide)<br>**SPN:** sialophorin

	28	30.215.525-30.732.465	**ADK:** Adenosine Kinase
**skin and hair pigmentation**	1	646.020-878.049	**HERC2:**HECT and RLD domain containing E3 ubiquitin protein ligase 2
	5	66.359.938-66.361.103	**PMCH:** pro-melanine concentrating hormone
	6	71.796.318-71.917.430	**KIT:**v-kit Hardy Zuckerman 4 feline sarcoma viral oncogene homolog
	10	46.241.889-46.311.618	**HERC1:** HECT and RLD domain containing E3 ubiquitin protein ligase1
	13	64.213.312-64.239.962	**ASIP:**agouti signalling protein
	18	14.757.332-14.759.082	**MC1R:**melanocortin 1 receptor
	26	22.623.887-22.626.506	**HPS6:**Hermansky-Pudlak syndrome 6
**sex determination and reproduction**	2	58.582.461-58.712.391<br>89.248.279-89.439.610	**NXPH2:** neurexophilin 2<br>**SPATS2L:** spermatogenesis associated, serine-rich 2 like
	3	121.098.866-121.111324	**BOK:** BCL 2- related ovarian killer 2
	6	46.064.179-46.067.119	**GDF9:** growth differentiation factor 9
	7	44.816.940-44.823.915	**BSG:** basigin
	9	89.967.417-90.256.185	**ESR1:** estrogen receptopr 1
	10	37.078.592-37.095.809<br>76.707.922-76.757.370	**TYRO3:** *TYRO3* protein tyrosine kinase<br>**ESR2:** estrogen receptor 2
	12	76.865.984-76.924.593	**DZIP1:** Daz interacting zinc finger protein 1
	13	23.728.842-23.795.499	**SPAG6:** sperm associated antigen 6
	15	61.763.306-61.767.248	**FSHB:** follicle stimulating hormone, beta polypeptide
	18	14.617.872-14.621.516	**SPATA2L:** spermatogenesis associated 2-like
	19	27.068.573-27.072.721<br>27.955.573-27.960.095	**SPAG7:** sperm associated antigen 7<br>**SHBG:** sex hormone binding globulin
	20	26.278.615-26.280.530	**PELO:** pelota homolog (Drosophila)
	24	50.786.812-50.809.920	**MRO:** maestro
	27	4.920.221-4.942.958<br>33.016.929-33.024.353	**SPAG11B:** sperm associated antigen11B<br>**STAR:** steroidogenic acute regulatory protein
**cartilage/bone formation and muscle biology**	1	81.151.205-81.166.512<br>81.202.139-81.209.052<br>76.685.699-76.721.920<br>153.971.502-154.22.489	**FETUB:** fetuin B<br>**AHSG:** alpha-2-hs-glycoprotein<br>**OSTN:** osteocrin<br>**CAPN7:** calpain 7

	2	39.516.817-39.517.123<br>6.213.566-6.220.196<br>5.350.654-5.407.857<br>8.901.432-9.030.990	**ACVR1C:** activin a receptpr, type 1C<br>**MSTN:** myostatin<br>**BIN1:** bridging integrator 1<br>**CALCRL:** calcitonin receptor like
	3	19.994.998-20.007.861<br>107.133.808-107.174.162	**CTSK:** cathepsin K<br>**BMP8A:** bone morphogenetic protein 8A
	4	63.052.081-63.305.357<br>52.589.574-52.812.810	**BMPER:** BMP binding endothelial regulator<br>**TFEC:** transcription factor EC
	5	106.157.909-106.169.922<br>77.502.140-77.528.803<br>86.571.273-87.036.289<br>10.330.469-10.342.660<br>95.456.475-95.459.983<br>75.550.903-75.568.048	**FGF6:** fibroblast growth factor 6<br>**YARS2:** tyrosyl TRNA synthetase 2, mitochondrial<br>**SOX5:** SRY (Sex determining region Y)-Box5<br>**MYF6** and **MYF5:** myogenic factors 6 and 5<br>**MGP:** matrix gla protein<br>**PVALB:** parvalbumin
	6	69.526.335-69.553.412	**SGCB:** sarcoglycan, beta (43kDa dystrophin-associated glycoprotein9
	7	44.896.887-44.903.232<br>49.62.892-49.905.978	**FSTL3:** follistatin-like3<br>**TGFBI:** transforming growth factor, beta-induced, 68 kDa
	8	71.278.582-71.390.893	**LOXL2:** lysyl oxidase-like 2
	10	66.751.294-66.755.024	**BMP4:** bone morphogenetic protein 4
	14	46.064.179-46.067.119	**GDF6:** growth differentiation factor6
	15	38.160.931-38.166.439	**CALCA:** calcitonin related polypeptide alpha
	16	37.106.632-37.143040<br>27.702.962-27.840.011	**DPT:** dermatopontin<br>**CAPN8** and **2**: calpain 8 and calpain 2, (M/II) large subunit
	18	13.186.023-13.261.250	**JPH3:** junctophilin3
	19	27.118.517-27.123.114<br>27.073.496-27.078.655	**CHRNE:** cholinergic receptor nicotinic epsilon (muscle)<br>**ENO3:** enolase 3 (beta, muscle)
	20	25.588.636-25.594.057	**FST:** follistatin
	21	45.519.651-45.522.303	**CFL2:** cofilin2 (muscle)
	25	26.815.266-26.817.728<br>41.372.194-41.390.545	**MYLPF:** myosin light chain, phosphorylatable, fast skeletal muscle<br>**CHST12:** carbohydrate (chondroitin 4) sulfotransferase
	26	14.667.438-14.848.281	**MYOF:** myoferlin
	28	41.817.915-41.875.992<br>26.253.844-26.406.119	**BMPR1A:** bone morphogenetic protein receptor type 1A<br>**COL13A1:** collagen, type XIII, alpha 1
	29	43.606.017-43.617.817	**PYGM:** phosphorylase, glycogen,

			muscle
**lipid metabolism**			
	1	70.375.620-70.664.027	**OSBPL11:** oxysterol binding protein-like 1
	2	5.878.707-6.032.482<br><br>111.797.170-111.887.645	**HIBC:** 3-hydroxyiisobutyryl-CoA-hydrolase<br>**ACSL3:** Acyl-CoA synthetase long chain family member 3
	3	69.344.157-69.382.504<br><br><br>49.136.317-49.225.714<br><br>100.317.759-100.338.715<br>80.071.689-80.147.000<br>80.243.543-80.254.246	**ACADM:** Acyl-CoA dehydrogenase C-4 to C-12 straight chain, mitochondrial<br>**ABCD3:** ATP-binding cassette, subfamily D (ALD), member 3<br>**FAAH:** fatty acid amide hydrolase<br>**LEPR:** leptin receptor<br>**LEPROT:** leptin receptor overlapping transcript
	5	80.665.380-80.712.457<br>117.150.568-117.233.112	**FAR2:** fatty acyl-CoA reductase<br>**PPARA:** peroxisome proiferator activated receptor alpha
	6	16.828.642-16.843.003<br><br>119.453.975-119.457.259	**PLA2G12A:** phospolipase A2, group XIIA<br>**ACOX3:** Acyl-CoA oxidase 3, pristanoyl
	10	59.227.895-59.282.939<br><br>28.331.829-28.339.131<br><br>21.149.062-21.149.352<br><br>37.346.275-37.355.599	**CYP19A1:** cytochrome p450, family 19, subfamily A, polypeptide 1<br>**LPCAT4:l**ysophosphatidylcholine acyltransferase 4<br>**CYP11A1:** cytochrome p450, family 11, subfamily A, polypeptide 1<br>**PLA2G4B:** phospholipase A2 group IVB (cytosolic)
	11	2.130.834-2.140.973<br><br>104.134.195-104.146.465<br><br>2.221.056-2.222.399	**GPAT2:** Glycerol-3-phosphate acyltransferase 2, mitochondrial<br>**AGPAT2:** 1-acylglycerol 3-phosphate-O- acyltransferase 2<br>**ADRA2B:** adrenoceptor alpha 2B
	13	51.150.852-51.173.199<br><br>64.794.728-64.841.520<br><br>55.451.215-55.483.467	**HAO1:** hydroxyacid oxidase (glycolate oxidase 1)<br>**ACSS2:** acetyl-coenzyme A synthetase, cytoplasmic<br>**OSBPL2:** oxysterol-binding protein-related protein 2
	14	39.529.627-39.605.792<br>24.747.192-24.772.996	**FAR1:** fatty acyl- CoA reductase 1<br>**TGS1:**trimethylguanosine synthase 1
	17	66.101.182-66.126.616	**ACACB:** acetyl CoA carboxylate, beta
	18	14.254.106-14.292.739	**ACSF3:** acyl- CoA synthetase family member 3

	19	63.230.261-63.243.062<br>27.568.223-27.573.378<br>39.253.265-39.265.055	**APOH:** apolipoprotein H (beta-2-glycoprotein 1)<br>**ACADVL:** acyl- CoA dehydrogenase very long chain<br>**OSBPL7:** oxysterol bnding protein like-7
	20	33.030.483-33.230.696	**PLCXD3:** phosphatidylinositol-specific phospholipase C, X domain containing 3
	21	34.314.628-34.321.218<br>45.302.738-45.328.878	**CYP1A2:** cytochrome p450, family 1, subfamily A, polypeptide 2<br>**SPTSSA:** serine palmytoyl transferase,small subunit A
	22	59.573.116-59.609.269<br>43.379.504-43.410.316	**ACAD9:** acyl- CoA-dehydrogenase family, member 9<br>**ACOX2:** acyl- CoA oxidase 2, branched chain
	23	49.909.573-49.924.201<br>10.166.999-10.223.697	**ECI2:** Enoyl -CoA delta isomerase 2<br>**PNPLA1:** patatin-like phospholipase domain containing 1
	25	38.842.836-38.866.581	**DAGLB:** diacylglycerol lipase, beta
	26	22.742.695-22.745.622<br>14.460.595-14.463.796<br>43.134.421-43.184.345<br>15.931.985-15.973.326<br>16.030.292-16.065.395<br>21.137.945-21.148.318	**ELOVL3:** fatty acid elongase 3<br>**CYP26A1:** cytochrome p450, family 26, subfamily A, polypeptide 1<br>**ACADSB:** acyl- CoA dehydrogenase, short/branched chain<br>**CYP2C18:** cytochrome p450, family 2, subfamily C, polypeptide 18<br>**CYP2C87:** cytochrome p450, family 2, subfamily C, polypeptide 87<br>**SCD:** stearoyl-CoA desaturase (delta-9 desaturase)
	27	4.679.600-4.727.246	**AGPAT5:** 1-acylglycerol-3-phosphate O-acyltramsferase 5
	28	3.378.615-3.418.362	**GNPAT:** glyceronephosphate O-acyltransferase
**carbohydrate metabolism**	1	107.181.763-107.215.036<br>103.088.853-103.204.728<br>28.659.456-28.972.472	**B3GALANT1:** beta-1,3-N-acetylgalactosaminyltransferase 1<br>**SI:** sucrose isomerase (Alpha-glucosidase)<br>**GBE1:** glucan (1,4-alpha) branching enxyme 1
	2	61.867.080-62.915.841	**LCT:** lactase
	4	77.820.949-77.857.152	**GCK:** glucokinase (hexokinase 4)
	5	25.627.052-25.631.237<br>88.287.352-88.453.869	**PPP1R1A:** protein phosphatase 1, regulatory (inhibitor)<br>**ST8SIA1:** ST8 alpha-N-acetyl-neuraminide alpha-2,8-sialyltransferase 1
	6	43.730.500-43.863.281	**GBA3:** glucosidase, beta, acid 3
	7	41.337.278-41.354.610	**MGAT1:** mannosyl (alpha-1,3)-

			glycoprotein beta-1,2-n-acetylglucosaminyltransferase 1
	8	82.396.095-82.438.817<br>77.234.223-77.236.116<br><br>77.352.975-77.356.425	**FBP2:** fructose-1,6-biphosphatase 2<br>**ENHO:** energy homeostasis associated<br>**GALT:** galactose-1- phosphate urydyltransferase
	9	32.092.887-32.265.123	**MAN1A1:** mannosyl-oligosaccaride 1,3-alpha-mannosidase IA-like
	10	81.396.103-81.493.501	**GALNTL1:** N-acetylgalactosaminyltransferase-like protein 1
	11	101.650.163-101.666.790<br><br>45.414.102-45.469.591<br><br>72.525.752-72.558.457<br>68.745.032-68.963.585<br><br><br>21.040.425-21.095.035	**POMT1:** protein-O-mannosyltransferase 1<br>**ST6GAL2:** ST6 beta-galactosamide aplha- 2,6 sialyltransferase 2<br>**KHK:** ketohexokinase (fructokinase)<br>**GALNT14:** UDP-N-acetyl-alpha-D-galactosamine:polypeptide N-acetylgalactosaminyltransferase 14<br>**GALM:** galactose mutarotase (aldolase-1-epimerase)
	13	62.237.057-62.277.832	**POFUT1:** O-fucosyltransferase 1
	18	1.681.354-1.694.462<br>1.748.257-1.762.838<br><br>15.370.927-15.405.215	**FUK:** fukokinase<br>**ST3GAL2:** ST3 beta galactoside alpha -2,3-sialyltransferase 2<br>**GPT2:** glutammic pyruvate transaminase (alanine aminotransferase) 2
	19	27.490.117-27.500.094	**ASGR2:** asialoglycoprotein receptor 2
	21	34.189.055-34.196.211<br>22.166.073-22.183.354	**MPI:** mannose phosphate isomerase<br>**MAN2A2:** mannosidase, alpha, class2A, member 2
	22	7.413.801-7.517.596	**GLB1:** galactosidase, beta 1
	24	30.037.089-30.315.707	**CHST9:** carbohydrate (N-acetylgalactosamine 4-0) sulfotransferase 9
	25	41.304.499-41.313.035<br><br><br>26.470.486-26.475.202	**LFNG:** O-fucosylpeptide 3-beta-N-acetylglucosaminyltransferase<br><br>**ALDOA:** aldolase A, fructose-biphosphate
**mammary gland biology and milk production**	1	49.850.609-50.069.718	**BCAR3:** breast cancer antiestrogen resistance 3
		25.595.262-25.598.164	**GLYCAM1:** glycosilation dependent cell adesion molecule (pseudogene)

	5	82.246.832-82.258.858	**PTHLH:** parathyroid hormone-like hormone
	6	37.959.536-38.030.586<br>71.373.513-71.421.283	**ABCG2:**ATP-binding cassette, sub-family G (WHITE), member 2<br>**PDGFRA:**platelet-derived growth factor receptor, alpha polypeptide
	20	31.890.736-32.199.996	**GHR:** Growth hormone receptor
	25	41.206.701-41.216.986<br>10.576.475-10.584.324	**BRAT1:** BRCA1-associated ATM activator 1<br>**BCAR4:** breast cancer anti-estrogen resistance 4
	26	22.375.029-22.380.835	**FGF8:** fibroblast growth factor 8
**Others**	2	84.734.002-84.773.232<br>71.561.192-71.566.372	**SLC39A10:** solute carrier family 39 (zinc transporter), member 10<br>**DBI:**diazepam binding inhibitor (GABA receptor,Acyl-CoA binding protein)
	3	108.651.328-108.691.376<br>110.135.910-110.208.496	**MTF1:** metal-regulatory transcription factor 1<br>**THRAP3:** thyroid hormone receptor associated protein 3
	4	120.427.002-120.496.083	**VIPR2:** vasoactive intestinal peptide 2
	5	89.258.893-89.311.142<br>110.570.728-110.582.589	**SLCO1A2:** solute carrier organic anion transporter family 1, member A2<br>**CSNK1E:** casein kinase 1, epsilon
	7	47.837.918-47.845.674<br>19.691.000-19.692.540	**CAMLG:** calcium modulating ligand<br>**CAPS:** calcyphosine
	8	71.587.511-71.590.578<br>77.259.610-77.298.858	**SLC25A37:** solute carrier family 25 (mitochondrial iron transporter) member 37<br>**CNTFR:** ciliary neurotrophic factor receptor
	13	47.400.392-47.418.507<br>47.444.352-47.449.390	**PRNP:** prion protein<br>**PRND:** prion protein 2 (dublet)
	15	39.728.332-39.730.868<br>22.838.240-22.905.945<br>48.997.631-49.074.735<br>52.572.870-52.583.730<br>52.602.819-52.605.539	**PTH:** parathyroid hormone<br>**BCO2:** beta carotene oxigenase 2<br>**HBE1, HBE4, HBB, HBE2, HBG, LOC788610:** haemoglobin family<br>**FOLR1:** folate receptor 1 (adult)<br>**FOLR2:** folate receptor 2 (fetal)

	17	67.543.375-67.571.022	**CRYBB3:** crystalline, beta B3<br>**CRYBB2:** crystalline, beta B2
	20	31.784.034-31.793.708	**SEPP1:** selenoprotein P, plasma1
	22	51.325.106-51.326.274	**GPX1:** glutathione peroxidase 1
	24	30.362.738-30.377.272	**AQP4:** aquaporin 4
	26	13.920.373-14.012.061	**IDE:** insulin degrading enzyme
	27	25.422.502-25.432.237	**LEPROTL1:** leptin receptor overlapping transcript-like 1
	28	41.644.490-41.654.402<br>24.556.240-24.582.756	**OPN4**: opsonin 4<br>**SIRT1:** sirtuin, type 1
