# Supplementary material for: Use of canonical discriminant analysis to study signatures of selection in cattle
Source: Genet Sel Evol. 2016 Aug 12;48:58. doi: 10.1186/s12711-016-0236-7 (PMC4983034; doi:10.1186/s12711-016-0236-7)
Supplement: Supplementary file 5 — 10.1186/s12711-016-0236-7 List of markers and genes identified by both CDA and FST approaches. This table reports the markers and the genes in common between the two considered approaches for detection of selection signatures. [file 12711_2016_236_MOESM5_ESM.pdf]

Gene	start	end	chrom	Position detected by Fst
TMEM44	73516015	73559862	1	73404401
CCDC50	76545411	76624452	1	76709967
TPRG1	78604072	78758431	1	78763254
TMEM37	71607733	71613808	2	71518065
TMEM37	71607733	71613808	2	71598307
STK40	110074347	110109948	3	110272602
ATG4B	121162790	121170613	3	121275236
ATG4B	121162790	121170613	3	121374825
IGFBP3	76705104	76713510	4	76943551
CCM2	77250090	77301198	4	77009788
CCM2	77250090	77301198	4	77150031
OGDH	77458514	77512436	4	77555681
OGDH	77458514	77512436	4	77581137
OGDH	77458514	77512436	4	77614815
OGDH	77458514	77512436	4	77635835
OGDH	77458514	77512436	4	77697936
FGF6	106157908	106169922	5	106230591
FGF6	106157908	106169922	5	106269362
USP46	69952425	70027020	6	69840434
HINT1	24515177	24521786	7	24655689
HINT1	24515177	24521786	7	24677021
POLRMT	44851397	44864889	7	44720216
LEAP2	46075147	46076065	7	46190019
H2AFY	48390757	48472188	7	48470968
MIR2285AA	56494702	56522356	7	56766754
PPP2R2B	59782766	60294948	7	60055026
EBF1	72394109	72804182	7	72882903
FSD1L	96964124	97006477	8	96743576
AMBP	105006305	105018463	8	104862806
TBX18	65579317	65608088	9	65738419
INPP4A	3686195	3738521	11	3518439
TPRN	105896210	105903700	11	105985714
SPRY2	55683998	55685894	12	55674695
MIR1256	57534532	57534614	12	57433505
ZMYND11	47195550	47256127	13	47101933
RASSF2	47492958	47515677	13	47368346
RASSF2	47492958	47515677	13	47415727
PCNA	47788527	47794602	13	47879982
TRMT6	48476001	48486536	13	48318066
FERMT1	48617011	48665879	13	48429772
HCK_AA	62106256	62151619	13	62259448
TGS1	24747191	24772996	14	24524205
TGS1	24747191	24772996	14	24573257
LYN	24847259	24920715	14	24607527
LYN	24847259	24920715	14	24643266
LYN	24847259	24920715	14	25107556
TIMM8B	22720234	22722218	15	22809416
MGST2	18331271	18369333	17	18366429
MGARP	18709902	18721279	17	18687182
MGARP	18709902	18721279	17	18742163
MGARP	18709902	18721279	17	18881179
MGARP	18709902	18721279	17	18965447
CCRN4L	18892760	18910926	17	19109434
UFD1L	74717682	74730989	17	74948921
DPEP1	14578305	14584239	18	14359452

CDK10	14610727	14617871	18	14401871
CDK10	14610727	14617871	18	14503218
UBE2G1	25409132	25496726	19	25458074
CDH9	46652610	46794454	20	46405056
HDDC3	22157122	22158987	21	22315417
KLF13	28220561	28221094	21	28424029
APBA2	28512733	28626136	21	28525114
LOC509354	52126503	52133264	22	52056494
LOC100126544	8269051	8271772	23	8513266
ZFAND3	11712801	12040964	23	11631005
PRPF4B	49953452	49977845	23	49725139
IRF4	52050984	52063706	23	51938161
DLGAP1	37994545	38293883	24	38158543
CHST12	41372193	41390545	25	41497446
DPCD	22205906	22227993	26	21977581
DPCD	22205906	22227993	26	22059103
DPCD	22205906	22227993	26	22339074
KCNIP2	22422212	22425506	26	22550366
KCNIP2	22422212	22425506	26	22587068
FBXL15	22917658	22919707	26	22913016
MBOAT4	25448499	25457512	27	25392013
FAM89A	3656712	3676337	28	3504349
NRXN2	43476402	43576128	29	43525624
NRXN2	43476402	43576128	29	43652252
NRXN2	43476402	43576128	29	43709769
NRXN2	43476402	43576128	29	43777249
MUS81	44644890	44650617	29	44649908
CCDC50	76545411	76624452	1	76709967
NMD3	106973279	107009693	1	107257059
B3GALNT1	107181762	107215036	1	107284449
SEMA3D	35563537	35789896	4	35354923
PKD2	38040870	38099549	6	38133743
LAP3	38574589	38600027	6	38576012
LAP3	38574589	38600027	6	38825835
LAP3	38574589	38600027	6	38845992
LCORL	38840863	38992112	6	38869785
CWH43	69282678	69335089	6	69482838
PDGFRA	71373512	71421283	6	71374574
SMARCA2	42652769	42830159	8	42405548
ARHGAP25	67066106	67158251	11	67185874
ARHGAP25	67066106	67158251	11	67257038
SPRY2	55683998	55685894	12	55583608
DNAJC3	76958977	77024110	12	76839422
ACBD7	29940116	29946543	13	29934974
KCNIP2	22422212	22425506	26	22587068
RTKN2	18363060	18471853	28	18472885
PDGFRA	71373512	71421283	6	71421017
PDGFRA	71373512	71421283	6	71476002
PDGFRA	71373512	71421283	6	71519635
KIT	71796317	71917430	6	71552977
KIT	71796317	71917430	6	71873004
TECRL	81511553	81653990	6	81767374
C7H5orf24	47910524	47917815	7	47807367
MIR1256	57534532	57534614	12	57465057
DNAJC3	76958977	77024110	12	76839422
RASSF2	47492958	47515677	13	47583553

SLC22A31	14321860	14326425	18	14208633
CUTC	20550821	20583525	26	20668949
PKD2	38040870	38099549	6	37925393
FAM151B	82923269	82957972	7	82998908
KRT8	73627763	73629541	10	73417064
UFD1L	74717682	74730989	17	74948921
DPEP1	14578305	14584239	18	14359452
CDK10	14610727	14617871	18	14401871
ZFAND3	11712801	12040964	23	11631005
DLGAP1	37994545	38293883	24	38158543
RAB27B	54653964	54715265	24	54542635
KCNIP2	22422212	22425506	26	22587068
RPL35A	70788694	70792140	1	70840701
RPL35A	70788694	70792140	1	70852215
ETS2	152880457	152891965	1	153045653
UBXN4	61919638	61949692	2	62008179
UBXN4	61919638	61949692	2	62080553
UBXN4	61919638	61949692	2	62106463
TMEFF2	80997732	81275827	2	81368765
SEP15	57285504	57285872	3	57283356
MIR653	10653526	10653616	4	10499057
GRM3	33769687	34021297	4	34059453
GRM3	33769687	34021297	4	34104507
GRM3	33769687	34021297	4	34132924
SEMA3D	35563537	35789896	4	35354923
PCED1B	33189702	33339728	5	33246646
CSF2RB	75724619	75745819	5	75627333
YARS2	77502139	77528803	5	77563893
PKD2	38040870	38099549	6	38133743
LAP3	38574589	38600027	6	38576012
LAP3	38574589	38600027	6	38825835
LAP3	38574589	38600027	6	38845992
LCORL	38840863	38992112	6	38869785
CTSL1	82365268	82371041	8	82125433
ARHGAP25	67066106	67158251	11	67108521
ARHGAP25	67066106	67158251	11	67185874
ARHGAP25	67066106	67158251	11	67257038
GFPT1	67684389	67740412	11	67498111
CAPN14	68678748	68715047	11	68860065
ITCH	64357861	64462220	13	64399733
CTNNBL1	67257418	67431112	13	67264571
TTI1	67528733	67559052	13	67530414
OLFML1	45836410	45866755	15	45667482
HBE4	49007000	49008555	15	48936679
ITFG1	15625547	15928962	18	16070657
ASGR1	27529096	27532634	19	27763447
MPDU1	27925700	27929096	19	27992667
MPDU1	27925700	27929096	19	28155641
C19H17orf59	28443495	28445326	19	28299528
FAM107A	43354327	43359047	22	43127183
LIMD1	54259647	54337446	22	54028803
DLGAP1	37994545	38293883	24	38307677
PAPSS2	9292115	9402158	26	9550462
KCNIP2	22422212	22425506	26	22587068
NRXN2	43476402	43576128	29	43269744
PDGFRA	71373512	71421283	6	71476002

PDGFRA	71373512	71421283	6	71519635
KIT	71796317	71917430	6	71552977
KIT	71796317	71917430	6	71873004
TECRL	81511553	81653990	6	81767374
DAPK1	82096667	82309886	8	81877902
PHF3	636458	715423	9	918988
SLC22A31	14321860	14326425	18	14208633
USP46	69952425	70027020	6	69840434
RASL11B	70179363	70183823	6	70209132
FSD1L	96964124	97006477	8	96743576
INPP4A	3686195	3738521	11	3518439
PCNA	47788527	47794602	13	47879982
TRMT6	48476001	48486536	13	48318066
FERMT1	48617011	48665879	13	48429772
RPS13	35943264	35945986	15	36026517
MGST2	18331271	18369333	17	18366429
DPEP1	14578305	14584239	18	14359452
CDK10	14610727	14617871	18	14401871
CDK10	14610727	14617871	18	14503218
FBXL15	22917658	22919707	26	22913016
MIR653	10653526	10653616	4	10499057
PDGFRA	71373512	71421283	6	71374574
CAPN14	68678748	68715047	11	68860065
DNAJC3	76958977	77024110	12	76839422
FBXO28	28009797	28033719	16	27833776
GLRB	42996912	43086245	17	43325722
SGTB	13805927	13851473	20	13653662
GPBP1	22033852	22098038	20	22093001
LYRM4	49282978	49374342	23	49332018
CYP26A1	14460594	14463796	26	14457492
RTKN2	18363060	18471853	28	18472885
SH3BP5	154023225	154103515	1	153919758
ORC4	48282733	48378021	2	48440885
WLS	77396242	77511241	3	77312933
CDK14	7844073	8440697	4	7870863
MLL5	46474530	46565717	4	46514849
MDFIC	53756421	53854749	4	54028906
PDGFRA	71373512	71421283	6	71421017
PDGFRA	71373512	71421283	6	71476002
PDGFRA	71373512	71421283	6	71519635
KIT	71796317	71917430	6	71552977
KIT	71796317	71917430	6	71873004
TECRL	81511553	81653990	6	81767374
SKP1	47389341	47401412	7	47252135
SKP1	47389341	47401412	7	47274866
C7H5orf24	47910524	47917815	7	47807367
FER	110465381	110864228	7	110397885
FER	110465381	110864228	7	110422232
CLCN3	1426803	1502615	8	1569883
GRIN3A	92944702	93145524	8	93276825
PHF3	636458	715423	9	918988
TSSK4	20786858	20789307	10	20899486
TSSK4	20786858	20789307	10	20932671
SRSF5	81860043	81865204	10	81648739
DNAJC3	76958977	77024110	12	76839422
ZW10	24616729	24652072	15	24809282

MAP1LC3B	13017743	13031055	18	13109858
SLC22A31	14321860	14326425	18	14208633
CDK10	14610727	14617871	18	14526709
RNF112	34667120	34672049	19	34802725
RNF112	34667120	34672049	19	34836416
MIR2285F-2	21839466	21839555	20	21938158
GPBP1	22033852	22098038	20	22093001
CCDC50	76545411	76624452	1	76709967
IL1RAP	77223041	77343645	1	77248361
MGA	37193025	37284139	10	37290812
PCNA	47788527	47794602	13	47879982
TRMT6	48476001	48486536	13	48318066
FERMT1	48617011	48665879	13	48429772
TIMM8B	22720234	22722218	15	22809416
DPEP1	14578305	14584239	18	14359452
CDK10	14610727	14617871	18	14401871
CDK10	14610727	14617871	18	14503218
DPCD	22205906	22227993	26	22339074
KCNIP2	22422212	22425506	26	22550366
KCNIP2	22422212	22425506	26	22587068
FBXL15	22917658	22919707	26	22913016
FGF12	75288094	75895971	1	75301239
CCDC50	76545411	76624452	1	76709967
MIR653	10653526	10653616	4	10499057
SEMA3D	35563537	35789896	4	35354923
LAP3	38574589	38600027	6	38576012
LAP3	38574589	38600027	6	38825835
LAP3	38574589	38600027	6	38845992
LCORL	38840863	38992112	6	38869785
G3BP1	64976047	65004835	7	65156756
CTSL1	82365268	82371041	8	82125433
ARHGAP25	67066106	67158251	11	67257038
GFPT1	67684389	67740412	11	67498111
CAPN14	68678748	68715047	11	68860065
DNAJC3	76958977	77024110	12	76839422
ITFG1	15625547	15928962	18	16070657
SGTB	13805927	13851473	20	13653662
GPBP1	22033852	22098038	20	22093001
KCNIP2	22422212	22425506	26	22587068
RTKN2	18363060	18471853	28	18472885
SH3BP5	154023225	154103515	1	153919758
WLS	77396242	77511241	3	77312933
CDK14	7844073	8440697	4	7870863
RAC2	76033362	76050188	5	76049319
RAC2	76033362	76050188	5	76200813
MFNG	76266276	76282792	5	76317361
KIT	71796317	71917430	6	71552977
KIT	71796317	71917430	6	71873004
TECRL	81511553	81653990	6	81767374
PHF3	636458	715423	9	918988
SRSF5	81860043	81865204	10	81648739
ARHGAP25	67066106	67158251	11	67347867
DNAJC3	76958977	77024110	12	76839422
SLC22A31	14321860	14326425	18	14208633
CDK10	14610727	14617871	18	14526709
RNF112	34667120	34672049	19	34802725

RNF112	34667120	34672049	19	34836416
MIR2285F-2	21839466	21839555	20	21938158
GPBP1	22033852	22098038	20	22093001
SLC40A1	6716590	6740329	2	6675045
SLC40A1	6716590	6740329	2	6763227
SLC40A1	6716590	6740329	2	6831955
CALCRL	8901431	9030990	2	9245063
DNTTIP2	49770191	49781520	3	49544712
DNTTIP2	49770191	49781520	3	49551407
USH2A	19573855	20502175	16	20449555
FST	25588635	25594057	20	25643166
C28H10orf107	17675506	17845827	28	17485412
TMEM44	73516015	73559862	1	73404401
STK40	110074347	110109948	3	110078547
STK40	110074347	110109948	3	110272602
ATG4B	121162790	121170613	3	121374825
MET	51912650	52042198	4	52138962
ZNF804B	73326979	73897041	4	74042008
IGFBP3	76705104	76713510	4	76943551
CCM2	77250090	77301198	4	77009788
CCM2	77250090	77301198	4	77150031
OGDH	77458514	77512436	4	77555681
OGDH	77458514	77512436	4	77581137
OGDH	77458514	77512436	4	77614815
OGDH	77458514	77512436	4	77635835
OGDH	77458514	77512436	4	77697936
MYO1A	56712796	56738770	5	56618321
CCDC53	66168578	66227000	5	66157408
FGF6	106157908	106169922	5	106230591
FGF6	106157908	106169922	5	106269362
MIRLET7A-3	117119384	117119458	5	117194638
SLIT2	41236269	41642320	6	41253845
USP46	69952425	70027020	6	69840434
RASL11B	70179363	70183823	6	70209132
NMU	72732590	72760814	6	72531373
HINT1	24515177	24521786	7	24655689
HINT1	24515177	24521786	7	24677021
LEAP2	46075147	46076065	7	46190019
MIR2285AA	56494702	56522356	7	56766754
PPP2R2B	59782766	60294948	7	60055026
EBF1	72394109	72804182	7	72882903
FREM1	29353417	29504029	8	29592011
ZDHHC21	29553186	29609485	8	29767566
CHMP7	71228580	71251803	8	71463417
LOC530027	32092886	32265123	9	32171013
TTBK2	38159316	38248606	10	38452236
LIPC	51758866	51921040	10	51715026
KRT8	73627763	73629541	10	73417064
GCC2	44669019	44702360	11	44439698
TNFRSF19	34673861	34757696	12	34635671
SPRY2	55683998	55685894	12	55674695
HCK_AA	62106256	62151619	13	62259448
TGS1	24747191	24772996	14	24524205
TGS1	24747191	24772996	14	24573257
LYN	24847259	24920715	14	24607527
LYN	24847259	24920715	14	24643266

LYN	24847259	24920715	14	25107556
RPS13	35943264	35945986	15	36026517
TMEM184C	10643725	10672726	17	10594226
MGST2	18331271	18369333	17	18366429
MGARP	18709902	18721279	17	18687182
MGARP	18709902	18721279	17	18742163
MGARP	18709902	18721279	17	18881179
MGARP	18709902	18721279	17	18965447
CCRN4L	18892760	18910926	17	19109434
DPEP1	14578305	14584239	18	14359452
CDK10	14610727	14617871	18	14401871
CDK10	14610727	14617871	18	14503218
UBE2G1	25409132	25496726	19	25458074
GHR	31890735	32199996	20	31848979
CDH9	46652610	46794454	20	46405056
HDDC3	22157122	22158987	21	22315417
STXBP6	35415157	35655955	21	35415545
STXBP6	35415157	35655955	21	35494765
KLHL18	52871072	52922306	22	52783353
KCTD20	10355457	10391460	23	10167082
PRPF4B	49953452	49977845	23	49725139
IRF4	52050984	52063706	23	51938161
ERLIN1	20921805	20963567	26	21180893
DPCD	22205906	22227993	26	21977581
DPCD	22205906	22227993	26	22059103
DPCD	22205906	22227993	26	22339074
KCNIP2	22422212	22425506	26	22550366
KCNIP2	22422212	22425506	26	22587068
FBXL15	22917658	22919707	26	22913016
ANK3	15818093	15936518	28	15890631
NRG3	37100608	38351432	28	37359315
NRXN2	43476402	43576128	29	43525624
NRXN2	43476402	43576128	29	43652252
NRXN2	43476402	43576128	29	43709769
NRXN2	43476402	43576128	29	43777249
LRFN4	45517058	45519576	29	45287502
LRFN4	45517058	45519576	29	45326585
LRFN4	45517058	45519576	29	45367095
UBXN4	61919638	61949692	2	61778818
UBXN4	61919638	61949692	2	62008179
UBXN4	61919638	61949692	2	62080553
UBXN4	61919638	61949692	2	62106463
CWH43	69282678	69335089	6	69482838
ARRDC5	20473897	20486896	7	20304019
SPRY2	55683998	55685894	12	55583608
CTNNBL1	67257418	67431112	13	67264571
TTI1	67528733	67559052	13	67530414
RIC3	44962851	45024903	15	44999012
HBE4	49007000	49008555	15	48936679
ASGR1	27529096	27532634	19	27763447
MPDU1	27925700	27929096	19	27992667
MPDU1	27925700	27929096	19	28155641
C19H17orf59	28443495	28445326	19	28299528
HMG20A	33083548	33160048	21	33113989
STXBP6	35415157	35655955	21	35448143
FAM107A	43354327	43359047	22	43127183

KCNIP2	22422212	22425506	26	22587068
CTNNA3	22418863	24299242	28	22911596
NRXN2	43476402	43576128	29	43269744
UTP11L	108529493	108546298	3	108311829
TECRL	81511553	81653990	6	81767374
SKP1	47389341	47401412	7	47252135
SKP1	47389341	47401412	7	47274866
C7H5orf24	47910524	47917815	7	47807367
C11H2orf81	10190053	10195937	11	9944217
SLC22A31	14321860	14326425	18	14208633
DPP3	45167008	45198893	29	45187114
RTCB	71366682	71388046	5	71546802
COX4I2	61745577	61750870	13	61947481
CCDC50	76545411	76624452	1	76709967
TPRG1	78604072	78758431	1	78763254
LPP	79041609	79725431	1	79034999
LPP	79041609	79725431	1	79324497
NMD3	106973279	107009693	1	107149909
PLCL1	86717861	87087608	2	87133202
LRRC8C	53717829	53810701	3	53730982
MANEAL	108701539	108708400	3	108528219
STK40	110074347	110109948	3	110078547
STK40	110074347	110109948	3	110272602
ATG4B	121162790	121170613	3	121275236
ATG4B	121162790	121170613	3	121374825
MET	51912650	52042198	4	52138962
IGFBP3	76705104	76713510	4	76943551
CCM2	77250090	77301198	4	77009788
CCM2	77250090	77301198	4	77150031
OGDH	77458514	77512436	4	77555681
OGDH	77458514	77512436	4	77581137
OGDH	77458514	77512436	4	77614815
OGDH	77458514	77512436	4	77635835
OGDH	77458514	77512436	4	77697936
MYO1A	56712796	56738770	5	56618321
FGF6	106157908	106169922	5	106230591
FGF6	106157908	106169922	5	106269362
MIRLET7A-3	117119384	117119458	5	117194638
USP46	69952425	70027020	6	69840434
RASL11B	70179363	70183823	6	70209132
NMU	72732590	72760814	6	72531373
POLRMT	44851397	44864889	7	44720216
LEAP2	46075147	46076065	7	46190019
MIR2285AA	56494702	56522356	7	56766754
PPP2R2B	59782766	60294948	7	60055026
EBF1	72394109	72804182	7	72882903
ZDHHC21	29553186	29609485	8	29767566
LOC530027	32092886	32265123	9	32171013
RMND1	89612880	89648757	9	89847787
FSIP1	35332359	35538497	10	35143318
MGA	37193025	37284139	10	37290812
UXS1	45583100	45640370	11	45346657
HTR2A	16823525	16889281	12	16699315
TGS1	24747191	24772996	14	24524205
TGS1	24747191	24772996	14	24573257
LYN	24847259	24920715	14	24607527

LYN	24847259	24920715	14	24643266
LYN	24847259	24920715	14	25107556
RPS13	35943264	35945986	15	36026517
MGARP	18709902	18721279	17	18687182
MGARP	18709902	18721279	17	18742163
MGARP	18709902	18721279	17	18881179
MGARP	18709902	18721279	17	18965447
CCRN4L	18892760	18910926	17	19109434
UFD1L	74717682	74730989	17	74948921
DPEP1	14578305	14584239	18	14359452
CDK10	14610727	14617871	18	14401871
CDK10	14610727	14617871	18	14503218
UBE2G1	25409132	25496726	19	25458074
CDH9	46652610	46794454	20	46405056
KLF13	28220561	28221094	21	28424029
APBA2	28512733	28626136	21	28525114
CNTN6	25053492	25373573	22	25141851
KCTD20	10355457	10391460	23	10167082
ZFAND3	11712801	12040964	23	11631005
PRPF4B	49953452	49977845	23	49725139
IRF4	52050984	52063706	23	51938161
DPCD	22205906	22227993	26	21977581
DPCD	22205906	22227993	26	22059103
DPCD	22205906	22227993	26	22339074
KCNIP2	22422212	22425506	26	22550366
KCNIP2	22422212	22425506	26	22587068
FBXL15	22917658	22919707	26	22913016
MBOAT4	25448499	25457512	27	25392013
ADAM32	33900737	34076024	27	33788321
ADAM32	33900737	34076024	27	33894649
C28H1orf198	3833550	3865974	28	3904667
NRG3	37100608	38351432	28	37359315
NRXN2	43476402	43576128	29	43652252
NRXN2	43476402	43576128	29	43709769
NRXN2	43476402	43576128	29	43777249
MUS81	44644890	44650617	29	44649908
LRFN4	45517058	45519576	29	45287502
LRFN4	45517058	45519576	29	45326585
LRFN4	45517058	45519576	29	45367095
CCDC50	76545411	76624452	1	76709967
NMD3	106973279	107009693	1	107257059
B3GALNT1	107181762	107215036	1	107284449
HERC2	646025	878049	2	657753
LAP3	38574589	38600027	6	38576012
LAP3	38574589	38600027	6	38825835
LAP3	38574589	38600027	6	38845992
LCORL	38840863	38992112	6	38869785
SMARCA2	42652769	42830159	8	42405548
PAPSS2	9292115	9402158	26	9550462
KCNIP2	22422212	22425506	26	22587068
ORC4	48282733	48378021	2	48440885
ALPL	131791674	131857642	2	131780984
UTP11L	108529493	108546298	3	108311829
NCAPG2	120133800	120207828	4	120389218
PHF3	636458	715423	9	918988
ASCC3	49714501	50103401	9	49952332

ASCC3	49714501	50103401	9	49974322
PTPLAD1	12383330	12413253	10	12200563
TSSK4	20786858	20789307	10	20899486
TSSK4	20786858	20789307	10	20932671
NAA30	70012192	70034767	10	70129609
MIR216B	38511117	38511199	11	38445947
ARHGAP25	67066106	67158251	11	67347867
KLF12	48831951	49184354	12	49395053
SLC22A31	14321860	14326425	18	14208633
CDK10	14610727	14617871	18	14526709
DPP3	45167008	45198893	29	45187114
LPP	79041609	79725431	1	79359163
RTCB	71366682	71388046	5	71546802
ZDHHC21	29553186	29609485	8	29831209
NMD3	106973279	107009693	1	107149909
LRRC8C	53717829	53810701	3	53730982
MET	51912650	52042198	4	52138962
PKD2	38040870	38099549	6	37925393
SLIT2	41236269	41642320	6	41253845
NMU	72732590	72760814	6	72531373
HINT1	24515177	24521786	7	24655689
HINT1	24515177	24521786	7	24677021
POLRMT	44851397	44864889	7	44720216
MGA	37193025	37284139	10	37290812
LIPC	51758866	51921040	10	51715026
KRT8	73627763	73629541	10	73417064
UFD1L	74717682	74730989	17	74948921
DPEP1	14578305	14584239	18	14359452
CDK10	14610727	14617871	18	14401871
CDK10	14610727	14617871	18	14503218
C1QTNF3	39760049	39792325	20	39538676
STXBP6	35415157	35655955	21	35415545
STXBP6	35415157	35655955	21	35494765
C28H1orf198	3833550	3865974	28	3904667
RPL35A	70788694	70792140	1	70840701
RPL35A	70788694	70792140	1	70852215
NMD3	106973279	107009693	1	107257059
B3GALNT1	107181762	107215036	1	107284449
UBXN4	61919638	61949692	2	62008179
TMEFF2	80997732	81275827	2	81368765
SEP15	57285504	57285872	3	57283356
GRM3	33769687	34021297	4	34059453
GRM3	33769687	34021297	4	34104507
GRM3	33769687	34021297	4	34132924
CSF2RB	75724619	75745819	5	75627333
PKD2	38040870	38099549	6	38133743
LAP3	38574589	38600027	6	38576012
LAP3	38574589	38600027	6	38825835
LAP3	38574589	38600027	6	38845992
LCORL	38840863	38992112	6	38869785
PDGFRA	71373512	71421283	6	71374574
GLDC	38468512	38557736	8	38518604
CTSL1	82365268	82371041	8	82125433
CTNNA2	54722278	56082035	11	54855548
ARHGAP25	67066106	67158251	11	67108521
ARHGAP25	67066106	67158251	11	67185874

ARHGAP25	67066106	67158251	11	67257038
ITCH	64357861	64462220	13	64399733
CTNNBL1	67257418	67431112	13	67264571
TTI1	67528733	67559052	13	67530414
RSRC2	55171374	55187332	17	55076312
ASGR1	27529096	27532634	19	27763447
MPDU1	27925700	27929096	19	27992667
MPDU1	27925700	27929096	19	28155641
C19H17orf59	28443495	28445326	19	28299528
HMG20A	33083548	33160048	21	33113989
STXBP6	35415157	35655955	21	35448143
DLGAP1	37994545	38293883	24	38307677
PAPSS2	9292115	9402158	26	9550462
NRXN2	43476402	43576128	29	43269744
MLL5	46474530	46565717	4	46514849
CSF2RB	75724619	75745819	5	75722589
CSF2RB	75724619	75745819	5	75757551
RAC2	76033362	76050188	5	76049319
RAC2	76033362	76050188	5	76200813
MFNG	76266276	76282792	5	76317361
PDGFRA	71373512	71421283	6	71421017
PDGFRA	71373512	71421283	6	71476002
PDGFRA	71373512	71421283	6	71519635
KIT	71796317	71917430	6	71552977
SKP1	47389341	47401412	7	47252135
SKP1	47389341	47401412	7	47274866
C7H5orf24	47910524	47917815	7	47807367
DAPK1	82096667	82309886	8	81866074
DAPK1	82096667	82309886	8	81877902
ARHGAP25	67066106	67158251	11	67347867
BMI1	23709228	23713074	13	23644481
TRIM29	31213141	31240291	15	31074059
UFD1L	74717682	74730989	17	74723634
SLC22A31	14321860	14326425	18	14208633
CDK10	14610727	14617871	18	14526709
RNF112	34667120	34672049	19	34836416
MIR2362	5654804	5654871	21	5742682
BUB3	43276922	43288958	26	43392412
LEF1	18335030	18450774	6	18213934
GPT2	15370926	15405215	18	15199711
LOC507820	46162636	46317067	1	45926861
LSAMP	61026445	61780987	1	61806466
TMEM44	73516015	73559862	1	73404401
CCDC50	76545411	76624452	1	76709967
IL1RAP	77223041	77343645	1	77248361
TPRG1	78604072	78758431	1	78763254
LPP	79041609	79725431	1	79034999
LPP	79041609	79725431	1	79324497
NAB1	5567015	5603683	2	5823066
INPP1	5867811	5903148	2	6151365
PLCL1	86717861	87087608	2	87133202
MIR2284U	130903851	130903927	2	131041728
MIR2284U	130903851	130903927	2	131089397
LRRC8C	53717829	53810701	3	53730982
CRYZ	70246970	70275575	3	70077512
MANEAL	108701539	108708400	3	108528219

STK40	110074347	110109948	3	110078547
STK40	110074347	110109948	3	110272602
ATG4B	121162790	121170613	3	121374825
MIR2417	50323244	50323319	4	50300237
MIR2417	50323244	50323319	4	50322082
MET	51912650	52042198	4	52138962
IGFBP3	76705104	76713510	4	76943551
CCM2	77250090	77301198	4	77009788
CCM2	77250090	77301198	4	77150031
OGDH	77458514	77512436	4	77555681
OGDH	77458514	77512436	4	77581137
OGDH	77458514	77512436	4	77614815
OGDH	77458514	77512436	4	77635835
OGDH	77458514	77512436	4	77697936
SLCO1B3	89323085	89391585	5	89568937
FGF6	106157908	106169922	5	106230591
FGF6	106157908	106169922	5	106269362
MIRLET7A-3	117119384	117119458	5	117194638
USP46	69952425	70027020	6	69840434
NMU	72732590	72760814	6	72531373
ADAMTS3	89162541	89460195	6	89172293
HINT1	24515177	24521786	7	24655689
HINT1	24515177	24521786	7	24677021
LEAP2	46075147	46076065	7	46190019
H2AFY	48390757	48472188	7	48470968
MIR2285AA	56494702	56522356	7	56766754
FREM1	29353417	29504029	8	29592011
ZDHHC21	29553186	29609485	8	29767566
AMBP	105006305	105018463	8	104862806
LOC530027	32092886	32265123	9	32171013
TBX18	65579317	65608088	9	65738419
FSIP1	35332359	35538497	10	35143318
GCNT3	50707611	50712733	10	50866684
CYP19A1	59227894	59282939	10	59470586
TNFRSF19	34673861	34757696	12	34635671
PTK6	54599896	54606206	13	54663649
TGS1	24747191	24772996	14	24524205
TGS1	24747191	24772996	14	24573257
LYN	24847259	24920715	14	24607527
LYN	24847259	24920715	14	24643266
LYN	24847259	24920715	14	25107556
RPS13	35943264	35945986	15	36026517
KCNK2	69865015	70009106	16	69795545
TMEM184C	10643725	10672726	17	10594226
MGARP	18709902	18721279	17	18687182
MGARP	18709902	18721279	17	18742163
MGARP	18709902	18721279	17	18881179
MGARP	18709902	18721279	17	18965447
CCRN4L	18892760	18910926	17	19109434
UFD1L	74717682	74730989	17	74948921
ATMIN	7755290	7770411	18	7819498
ATMIN	7755290	7770411	18	7840467
DPEP1	14578305	14584239	18	14359452
CDK10	14610727	14617871	18	14401871
CDK10	14610727	14617871	18	14503218
MYO1C	23168432	23191280	19	23397071

UBE2G1	25409132	25496726	19	25458074
HS3ST3B1	32829906	32869783	19	33112218
CDH9	46652610	46794454	20	46405056
KLHL18	52871072	52922306	22	52783353
ZFAND3	11712801	12040964	23	11631005
PRPF4B	49953452	49977845	23	49725139
IRF4	52050984	52063706	23	51938161
DPCD	22205906	22227993	26	21977581
DPCD	22205906	22227993	26	22059103
DPCD	22205906	22227993	26	22339074
KCNIP2	22422212	22425506	26	22550366
KCNIP2	22422212	22425506	26	22587068
FBXL15	22917658	22919707	26	22913016
ADAM32	33900737	34076024	27	33788321
ADAM32	33900737	34076024	27	33894649
FAM89A	3656712	3676337	28	3504349
NRG3	37100608	38351432	28	37359315
NRXN2	43476402	43576128	29	43525624
NRXN2	43476402	43576128	29	43652252
NRXN2	43476402	43576128	29	43709769
NRXN2	43476402	43576128	29	43777249
MUS81	44644890	44650617	29	44649908
LRFN4	45517058	45519576	29	45287502
LRFN4	45517058	45519576	29	45326585
LRFN4	45517058	45519576	29	45367095
CCDC50	76545411	76624452	1	76709967
CWH43	69282678	69335089	6	69482838
PDGFRA	71373512	71421283	6	71374574
SMARCA2	42652769	42830159	8	42405548
ARHGAP25	67066106	67158251	11	67185874
ARHGAP25	67066106	67158251	11	67257038
KCNIP2	22422212	22425506	26	22587068
NRXN2	43476402	43576128	29	43269744
UTP11L	108529493	108546298	3	108311829
LOC787518	58597619	58598558	5	58676713
PDGFRA	71373512	71421283	6	71421017
PDGFRA	71373512	71421283	6	71476002
PDGFRA	71373512	71421283	6	71519635
KIT	71796317	71917430	6	71552977
SKP1	47389341	47401412	7	47252135
SKP1	47389341	47401412	7	47274866
C7H5orf24	47910524	47917815	7	47807367
NAA30	70012192	70034767	10	70129609
C11H2orf81	10190053	10195937	11	9944217
MIR216B	38511117	38511199	11	38445947
ITGB1BP1	87994385	88010373	11	87913641
KLF12	48831951	49184354	12	49395053
GID8	54936106	54942283	13	54763115
PHOX2A	52623068	52627543	15	52727193
SLC22A31	14321860	14326425	18	14208633
CYP46A1	66321421	66349422	21	66177498
DPP3	45167008	45198893	29	45187114
SLC40A1	6716590	6740329	2	6675045
SLC40A1	6716590	6740329	2	6763227
SLC40A1	6716590	6740329	2	6831955
C4H7orf10	80866408	81642062	4	80682403

LPHN3	78734710	79313831	6	78598487
POU4F3	59524544	59525876	7	59766632
ZDHHC21	29553186	29609485	8	29831209
CDADC1	18962898	18991285	12	18804912
EDN3	57571330	57596870	13	57570093
MIR2379	30788152	30788230	23	30953561
CUTC	20550821	20583525	26	20668949
NAB1	5567015	5603683	2	5823066
INPP1	5867811	5903148	2	6151365
PKD2	38040870	38099549	6	37925393
SLIT2	41236269	41642320	6	41253845
KCNIP4	41707927	41837538	6	41942484
KRT8	73627763	73629541	10	73417064
GCC2	44669019	44702360	11	44439698
UFD1L	74717682	74730989	17	74948921
STXBP6	35415157	35655955	21	35415545
STXBP6	35415157	35655955	21	35494765
LOC100126544	8269051	8271772	23	8513266
KCTD20	10355457	10391460	23	10167082
ZFAND3	11712801	12040964	23	11631005
MIR2390	40643968	40644040	25	40655616
UBXN4	61919638	61949692	2	62008179
UBXN4	61919638	61949692	2	62080553
UBXN4	61919638	61949692	2	62106463
GRM3	33769687	34021297	4	34059453
RTCB	71366682	71388046	5	71176757
CSF2RB	75724619	75745819	5	75627333
LAP3	38574589	38600027	6	38576012
LAP3	38574589	38600027	6	38825835
LAP3	38574589	38600027	6	38845992
LCORL	38840863	38992112	6	38869785
ARRDC5	20473897	20486896	7	20304019
RPS12	71974859	71978201	9	71736363
MEIS2	32643641	32868269	10	32998282
ARHGAP25	67066106	67158251	11	67108521
ARHGAP25	67066106	67158251	11	67185874
ARHGAP25	67066106	67158251	11	67257038
ITCH	64357861	64462220	13	64399733
CTNNBL1	67257418	67431112	13	67264571
TTI1	67528733	67559052	13	67530414
ASGR1	27529096	27532634	19	27763447
MPDU1	27925700	27929096	19	27992667
MPDU1	27925700	27929096	19	28155641
C19H17orf59	28443495	28445326	19	28299528
STXBP6	35415157	35655955	21	35448143
FAM107A	43354327	43359047	22	43127183
GLO1	12483467	12509232	23	12663012
PAPSS2	9292115	9402158	26	9550462
CSF2RB	75724619	75745819	5	75722589
CSF2RB	75724619	75745819	5	75757551
RAC2	76033362	76050188	5	76049319
RAC2	76033362	76050188	5	76200813
MFNG	76266276	76282792	5	76317361
TECRL	81511553	81653990	6	81767374
ARHGAP25	67066106	67158251	11	67347867
UFD1L	74717682	74730989	17	74723634

CHL1	26101270	26322519	22	26294371
GLO1	12483467	12509232	23	12663012
NLGN1	93626046	94742072	1	94585445
SLC40A1	6716590	6740329	2	6675045
SLC40A1	6716590	6740329	2	6763227
SLC40A1	6716590	6740329	2	6831955
CALCRL	8901431	9030990	2	9245063
DNTTIP2	49770191	49781520	3	49544712
DNTTIP2	49770191	49781520	3	49551407
RFX4	70323197	70395056	5	70258225
LEF1	18335030	18450774	6	18213934
IL1RAP	77223041	77343645	1	77248361
NMD3	106973279	107009693	1	107149909
NAB1	5567015	5603683	2	5823066
INPP1	5867811	5903148	2	6151365
MIR2417	50323244	50323319	4	50322082
MET	51912650	52042198	4	52138962
KCNIP4	41707927	41837538	6	41942484
RASL11B	70179363	70183823	6	70209132
NMU	72732590	72760814	6	72531373
LEAP2	46075147	46076065	7	46190019
MGA	37193025	37284139	10	37290812
LIPC	51758866	51921040	10	51715026
RPS13	35943264	35945986	15	36026517
DPEP1	14578305	14584239	18	14359452
CDK10	14610727	14617871	18	14401871
CDK10	14610727	14617871	18	14503218
PIGN	60978528	61087325	24	61140716
C28H1orf198	3833550	3865974	28	3904667
NMD3	106973279	107009693	1	107257059
B3GALNT1	107181762	107215036	1	107284449
LAP3	38574589	38600027	6	38576012
LAP3	38574589	38600027	6	38825835
LAP3	38574589	38600027	6	38845992
LCORL	38840863	38992112	6	38869785
PDGFRA	71373512	71421283	6	71374574
GLDC	38468512	38557736	8	38518604
IL33	38724601	38792047	8	38976903
CTSL1	82365268	82371041	8	82125433
RSPO2	58537058	58709246	14	58491253
AQP4	30362737	30377272	24	30572119
MLL5	46474530	46565717	4	46514849
MDFIC	53756421	53854749	4	54028906
PDGFRA	71373512	71421283	6	71421017
PDGFRA	71373512	71421283	6	71476002
PDGFRA	71373512	71421283	6	71519635
KIT	71796317	71917430	6	71552977
KIT	71796317	71917430	6	71873004
CLK4	41100795	41122562	7	41257488
SKP1	47389341	47401412	7	47252135
SKP1	47389341	47401412	7	47274866
C7H5orf24	47910524	47917815	7	47807367
CLCN3	1426803	1502615	8	1569883
PHF3	636458	715423	9	918988
TSSK4	20786858	20789307	10	20899486
TSSK4	20786858	20789307	10	20932671

ZW10	24616729	24652072	15	24809282
TRIM29	31213141	31240291	15	31074059
SLC22A31	14321860	14326425	18	14208633
CDK10	14610727	14617871	18	14526709
OPA1	74382911	74467420	1	74253662
SLC40A1	6716590	6740329	2	6675045
SLC40A1	6716590	6740329	2	6763227
SLC40A1	6716590	6740329	2	6831955
CALCRL	8901431	9030990	2	9245063
DNTTIP2	49770191	49781520	3	49544712
DNTTIP2	49770191	49781520	3	49551407
ATP6V0E2	113528364	113533518	4	113721793
RFX4	70323197	70395056	5	70258225
LEF1	18335030	18450774	6	18213934
NMU	72732590	72760814	6	72969608
CEP135	73021613	73095955	6	73032896
SOBP	42923994	43070235	9	43144718
PLA2G4B	37346274	37355599	10	37594262
CYP1B1	20493493	20499001	11	20729795
PSMB7	95401673	95458914	11	95322986
EDN3	57571330	57596870	13	57570093
TRIM29	31213141	31240291	15	31265444
USH2A	19573855	20502175	16	20449555
GPT2	15370926	15405215	18	15199711
FST	25588635	25594057	20	25643166

marker	Canonical sc	CVA	Comparison
Hapmap40716-BTA-34647	0,664938	1	bru_fri
BTA-95639-no-rs	0,568662	1	bru_fri
Hapmap26500-BTA-37512	0,6107086	1	bru_fri
Hapmap40177-BTA-47989	0,6056881	1	bru_fri
ARS-BFGL-NGS-103138	0,609059	1	bru_fri
ARS-BFGL-NGS-105427	0,7537367	1	bru_fri
ARS-BFGL-NGS-74724	0,654843	1	bru_fri
ARS-BFGL-NGS-12670	0,7030417	1	bru_fri
ARS-BFGL-NGS-110085	0,7268444	1	bru_fri
ARS-BFGL-NGS-37807	0,5951922	1	bru_fri
ARS-BFGL-NGS-16805	0,7946414	1	bru_fri
Hapmap53144-ss46525999	0,960147	1	bru_fri
ARS-BFGL-NGS-18303	0,8438817	1	bru_fri
ARS-BFGL-NGS-33546	0,6213246	1	bru_fri
ARS-BFGL-NGS-116590	0,9619391	1	bru_fri
ARS-BFGL-NGS-105631	0,6074869	1	bru_fri
BTA-74881-no-rs	0,6675708	1	bru_fri
ARS-BFGL-NGS-39379	0,8135405	1	bru_fri
Hapmap50117-BTA-81807	0,5778375	1	bru_fri
Hapmap49309-BTA-78604	0,6857792	1	bru_fri
BTB-00300220	0,7010296	1	bru_fri
ARS-BFGL-NGS-74330	0,6877488	1	bru_fri
Hapmap42327-BTA-78943	0,6650354	1	bru_fri
BTB-00310975	0,6804365	1	bru_fri
ARS-BFGL-NGS-116933	0,6581692	1	bru_fri
BTB-00313206	0,737734	1	bru_fri
Hapmap51050-BTA-79767	0,6638049	1	bru_fri
BTB-01729666	0,5893041	1	bru_fri
BTA-82649-no-rs	0,6072671	1	bru_fri
Hapmap34494-BES3_Contig286_1171	0,6079334	1	bru_fri
ARS-BFGL-NGS-115122	0,6274592	1	bru_fri
ARS-BFGL-NGS-82102	0,664875	1	bru_fri
Hapmap56826-rs29013564	0,6212727	1	bru_fri
BTB-01537438	0,677727	1	bru_fri
Hapmap53245-rs29026914	0,6545575	1	bru_fri
Hapmap39323-BTA-32823	0,6736954	1	bru_fri
BTB-01997512	0,7897329	1	bru_fri
Hapmap31215-BTA-32775	0,6734826	1	bru_fri
BTB-01718516	0,6556383	1	bru_fri
ARS-BFGL-NGS-3711	0,7326187	1	bru_fri
ARS-BFGL-NGS-83446	0,8101838	1	bru_fri
BTB-01530788	0,7214745	1	bru_fri
BTB-01530836	0,7177496	1	bru_fri
BTB-00557585	0,7442591	1	bru_fri
BTB-00557532	0,7655035	1	bru_fri
Hapmap41234-BTA-34285	0,7146203	1	bru_fri
BTB-00583707	0,5840195	1	bru_fri
ARS-BFGL-NGS-11296	0,596172	1	bru_fri
ARS-BFGL-NGS-24805	0,6253266	1	bru_fri
Hapmap41529-BTA-40591	0,6234888	1	bru_fri
Hapmap40057-BTA-25640	0,758145	1	bru_fri
ARS-BFGL-NGS-118491	0,6114943	1	bru_fri
Hapmap34713-BES10_Contig634_914	0,5980261	1	bru_fri
ARS-BFGL-NGS-24012	0,6523467	1	bru_fri
ARS-BFGL-NGS-86599	0,7457531	1	bru_fri

Hapmap44238-BTA-42338	0,8433857	1	bru_fri
ARS-BFGL-NGS-24837	0,8341293	1	bru_fri
Hapmap49615-BTA-44891	0,6958406	1	bru_fri
BTA-50702-no-rs	0,8122473	1	bru_fri
BTA-51687-no-rs	0,663332	1	bru_fri
BTB-01176930	0,654425	1	bru_fri
ARS-BFGL-NGS-64419	0,6551707	1	bru_fri
ARS-BFGL-NGS-24963	0,6932119	1	bru_fri
BTA-57141-no-rs	0,7177956	1	bru_fri
ARS-BFGL-NGS-18676	0,7260827	1	bru_fri
ARS-BFGL-NGS-73903	0,6930115	1	bru_fri
Hapmap57192-rs29027634	0,7708651	1	bru_fri
BTA-58128-no-rs	0,6828047	1	bru_fri
ARS-BFGL-NGS-36624	0,6461284	1	bru_fri
ARS-BFGL-NGS-116481	0,7152883	1	bru_fri
ARS-BFGL-NGS-6259	0,7064819	1	bru_fri
Hapmap53405-rs29014382	0,8713812	1	bru_fri
ARS-BFGL-NGS-101647	0,8877708	1	bru_fri
Hapmap24165-BTA-60897	0,7142751	1	bru_fri
ARS-BFGL-NGS-11271	0,8556078	1	bru_fri
ARS-BFGL-NGS-60861	0,5973874	1	bru_fri
ARS-BFGL-NGS-75049	0,6853566	1	bru_fri
ARS-BFGL-NGS-110358	0,6722806	1	bru_fri
ARS-BFGL-NGS-38896	0,7266489	1	bru_fri
ARS-BFGL-NGS-59421	0,7443347	1	bru_fri
ARS-BFGL-NGS-37441	0,7089464	1	bru_fri
ARS-BFGL-NGS-113797	0,6640138	1	bru_fri
BTA-95639-no-rs	0,5620555	2	bru_fri
BTB-00047642	0,4864703	2	bru_fri
BTB-00047563	0,4857291	2	bru_fri
BTB-02009505	0,4677709	2	bru_fri
Hapmap27072-BTC-033816	0,5926193	2	bru_fri
Hapmap26308-BTC-057761	0,7880093	2	bru_fri
Hapmap27083-BTC-041166	0,6402448	2	bru_fri
Hapmap23507-BTC-041133	0,5962648	2	bru_fri
Hapmap31285-BTC-041097	0,6185824	2	bru_fri
ARS-BFGL-NGS-20354	0,5883513	2	bru_fri
ARS-BFGL-NGS-109884	0,6518992	2	bru_fri
BTB-01581546	0,4742124	2	bru_fri
ARS-BFGL-NGS-64740	0,5144729	2	bru_fri
ARS-BFGL-NGS-103217	0,5130644	2	bru_fri
ARS-BFGL-BAC-14372	0,4683442	2	bru_fri
ARS-BFGL-NGS-4815	0,4473153	2	bru_fri
Hapmap25787-BTA-122982	0,5271526	2	bru_fri
Hapmap24165-BTA-60897	0,4540095	2	bru_fri
ARS-BFGL-NGS-102609	0,502253	2	bru_fri
Hapmap33128-BTC-041916	0,6216857	3	bru_fri
ARS-BFGL-NGS-38827	0,6210556	3	bru_fri
Hapmap27692-BTC-042876	0,6231059	3	bru_fri
Hapmap32220-BTC-042831	0,6117028	3	bru_fri
Hapmap31616-BTC-042811	0,6720923	3	bru_fri
Hapmap56688-rs29025335	0,6225131	3	bru_fri
BTB-00310752	0,443132	3	bru_fri
BTB-01537402	0,4658537	3	bru_fri
ARS-BFGL-NGS-4815	0,4956461	3	bru_fri
Hapmap49571-BTA-32781	0,4780062	3	bru_fri

ARS-BFGL-NGS-31386	0,55916	3	bru_fri
Hapmap35702-SCAFFOLD240765_1255	0,358391	4	bru_fri
ARS-BFGL-NGS-43280	0,6849637	1	bru_mar
ARS-BFGL-NGS-11872	0,7204157	1	bru_mar
BTB-01332940	0,7059931	1	bru_mar
ARS-BFGL-NGS-24012	0,6523467	1	bru_mar
ARS-BFGL-NGS-86599	0,7457531	1	bru_mar
Hapmap44238-BTA-42338	0,8433857	1	bru_mar
ARS-BFGL-NGS-18676	0,7260827	1	bru_mar
BTA-58128-no-rs	0,6828047	1	bru_mar
ARS-BFGL-NGS-116424	0,6475809	1	bru_mar
Hapmap24165-BTA-60897	0,7142751	1	bru_mar
Hapmap59145-ss46526952	0,503495	2	bru_mar
Hapmap48672-BTA-17044	0,4906196	2	bru_mar
ARS-BFGL-NGS-11586	0,5002801	2	bru_mar
Hapmap42558-BTA-47788	0,5663018	2	bru_mar
Hapmap59807-rs29011612	0,4340111	2	bru_mar
Hapmap60325-rs29019351	0,5639215	2	bru_mar
Hapmap33253-BTA-160512	0,5160511	2	bru_mar
ARS-BFGL-NGS-2985	0,4738744	2	bru_mar
Hapmap40298-BTA-109122	0,4494021	2	bru_mar
BTB-01934112	0,4972097	2	bru_mar
BTB-01144391	0,4994017	2	bru_mar
BTA-97585-no-rs	0,5044966	2	bru_mar
BTB-02009505	0,4677709	2	bru_mar
Hapmap46916-BTA-105154	0,5701775	2	bru_mar
ARS-BFGL-NGS-112184	0,5655324	2	bru_mar
ARS-BFGL-NGS-101198	0,6245147	2	bru_mar
Hapmap27072-BTC-033816	0,5926193	2	bru_mar
Hapmap26308-BTC-057761	0,7880093	2	bru_mar
Hapmap27083-BTC-041166	0,6402448	2	bru_mar
Hapmap23507-BTC-041133	0,5962648	2	bru_mar
Hapmap31285-BTC-041097	0,6185824	2	bru_mar
ARS-BFGL-NGS-4606	0,5282284	2	bru_mar
Hapmap30504-BTA-126653	0,5223383	2	bru_mar
ARS-BFGL-NGS-64740	0,5144729	2	bru_mar
ARS-BFGL-NGS-103217	0,5130644	2	bru_mar
ARS-BFGL-NGS-18300	0,5380863	2	bru_mar
ARS-BFGL-NGS-108785	0,5089836	2	bru_mar
Hapmap54972-rs29014977	0,5366218	2	bru_mar
BTA-33373-no-rs	0,5422195	2	bru_mar
ARS-BFGL-NGS-3823	0,5213345	2	bru_mar
Hapmap57286-ss46526482	0,4790361	2	bru_mar
Hapmap48733-BTA-36980	0,4928559	2	bru_mar
Hapmap27031-BTA-161389	0,5497927	2	bru_mar
BTB-01681968	0,5309286	2	bru_mar
ARS-BFGL-BAC-36986	0,4988945	2	bru_mar
ARS-BFGL-BAC-27962	0,5184964	2	bru_mar
BTB-00742339	0,4879395	2	bru_mar
Hapmap53021-rs29009645	0,459272	2	bru_mar
ARS-BFGL-NGS-51980	0,4530227	2	bru_mar
Hapmap31875-BTA-58099	0,5806762	2	bru_mar
ARS-BFGL-NGS-4670	0,4189025	2	bru_mar
Hapmap24165-BTA-60897	0,4540095	2	bru_mar
ARS-BFGL-NGS-5027	0,4374747	2	bru_mar
ARS-BFGL-NGS-38827	0,6210556	3	bru_mar

Hapmap27692-BTC-042876	0,6231059	3	bru_mar
Hapmap32220-BTC-042831	0,6117028	3	bru_mar
Hapmap31616-BTC-042811	0,6720923	3	bru_mar
Hapmap56688-rs29025335	0,6225131	3	bru_mar
Hapmap47422-BTA-82035	0,4518154	3	bru_mar
Hapmap38580-BTA-122605	0,5054258	3	bru_mar
ARS-BFGL-NGS-31386	0,55916	3	bru_mar
Hapmap50117-BTA-81807	0,5778375	1	bru_pez
Hapmap60836-rs29027147	0,5788707	1	bru_pez
BTB-01729666	0,5893041	1	bru_pez
ARS-BFGL-NGS-115122	0,6274592	1	bru_pez
Hapmap31215-BTA-32775	0,6734826	1	bru_pez
BTB-01718516	0,6556383	1	bru_pez
ARS-BFGL-NGS-3711	0,7326187	1	bru_pez
Hapmap36131-SCAFFOLD280927_4409	0,6797111	1	bru_pez
ARS-BFGL-NGS-11296	0,596172	1	bru_pez
ARS-BFGL-NGS-86599	0,7457531	1	bru_pez
Hapmap44238-BTA-42338	0,8433857	1	bru_pez
ARS-BFGL-NGS-24837	0,8341293	1	bru_pez
ARS-BFGL-NGS-11271	0,8556078	1	bru_pez
Hapmap40298-BTA-109122	0,4494021	2	bru_pez
ARS-BFGL-NGS-109884	0,6518992	2	bru_pez
ARS-BFGL-NGS-108785	0,5089836	2	bru_pez
ARS-BFGL-NGS-4815	0,4473153	2	bru_pez
ARS-BFGL-NGS-85727	0,5295738	2	bru_pez
BTB-01904985	0,4467614	2	bru_pez
ARS-BFGL-NGS-54907	0,4987952	2	bru_pez
ARS-BFGL-NGS-34923	0,4758882	2	bru_pez
BTB-01336729	0,4662142	2	bru_pez
Hapmap49385-BTA-94287	0,4366658	2	bru_pez
ARS-BFGL-NGS-102609	0,502253	2	bru_pez
Hapmap44395-BTA-64864	0,4139436	3	bru_pez
Hapmap43863-BTA-47554	0,5009512	3	bru_pez
BTB-01923614	0,3930325	3	bru_pez
ARS-BFGL-NGS-61702	0,490203	3	bru_pez
BTB-00182760	0,4685279	3	bru_pez
Hapmap33484-BTA-142221	0,4423976	3	bru_pez
Hapmap33128-BTC-041916	0,6216857	3	bru_pez
ARS-BFGL-NGS-38827	0,6210556	3	bru_pez
Hapmap27692-BTC-042876	0,6231059	3	bru_pez
Hapmap32220-BTC-042831	0,6117028	3	bru_pez
Hapmap31616-BTC-042811	0,6720923	3	bru_pez
Hapmap56688-rs29025335	0,6225131	3	bru_pez
ARS-BFGL-NGS-12557	0,7294468	3	bru_pez
ARS-BFGL-NGS-20141	0,4259094	3	bru_pez
BTB-00310752	0,443132	3	bru_pez
Hapmap53962-rs29017056	0,4113754	3	bru_pez
Hapmap3177-BTA-16454	0,416305	3	bru_pez
BTA-111263-no-rs	0,4330121	3	bru_pez
BTA-105718-no-rs	0,4671884	3	bru_pez
Hapmap38580-BTA-122605	0,5054258	3	bru_pez
ARS-BFGL-NGS-52654	0,4265274	3	bru_pez
ARS-BFGL-NGS-19848	0,4064174	3	bru_pez
Hapmap40806-BTA-77302	0,4306957	3	bru_pez
ARS-BFGL-NGS-4815	0,4956461	3	bru_pez
ARS-BFGL-NGS-27072	0,4308096	3	bru_pez

Hapmap58715-rs29021176	0,5036422	3	bru_pez
ARS-BFGL-NGS-31386	0,55916	3	bru_pez
ARS-BFGL-NGS-13803	0,7389009	3	bru_pez
ARS-BFGL-NGS-74800	0,4044331	3	bru_pez
ARS-BFGL-NGS-1097	0,3850229	3	bru_pez
ARS-BFGL-NGS-110975	0,4470808	3	bru_pez
ARS-BFGL-NGS-34923	0,4231008	3	bru_pez
BTA-95639-no-rs	0,568662	1	bru_pie
BTA-36624-no-rs	0,586048	1	bru_pie
ARS-BFGL-NGS-33594	0,6187352	1	bru_pie
Hapmap31215-BTA-32775	0,6734826	1	bru_pie
BTB-01718516	0,6556383	1	bru_pie
ARS-BFGL-NGS-3711	0,7326187	1	bru_pie
BTB-00583707	0,5840195	1	bru_pie
ARS-BFGL-NGS-86599	0,7457531	1	bru_pie
Hapmap44238-BTA-42338	0,8433857	1	bru_pie
ARS-BFGL-NGS-24837	0,8341293	1	bru_pie
Hapmap53405-rs29014382	0,8713812	1	bru_pie
ARS-BFGL-NGS-101647	0,8877708	1	bru_pie
Hapmap24165-BTA-60897	0,7142751	1	bru_pie
ARS-BFGL-NGS-11271	0,8556078	1	bru_pie
ARS-BFGL-NGS-37067	0,5452489	2	bru_pie
BTA-95639-no-rs	0,5620555	2	bru_pie
Hapmap40298-BTA-109122	0,4494021	2	bru_pie
BTB-02009505	0,4677709	2	bru_pie
Hapmap26308-BTC-057761	0,7880093	2	bru_pie
Hapmap27083-BTC-041166	0,6402448	2	bru_pie
Hapmap23507-BTC-041133	0,5962648	2	bru_pie
Hapmap31285-BTC-041097	0,6185824	2	bru_pie
BTA-100740-no-rs	0,4467522	2	bru_pie
ARS-BFGL-NGS-4606	0,5282284	2	bru_pie
ARS-BFGL-NGS-103217	0,5130644	2	bru_pie
ARS-BFGL-NGS-18300	0,5380863	2	bru_pie
ARS-BFGL-NGS-108785	0,5089836	2	bru_pie
ARS-BFGL-NGS-4815	0,4473153	2	bru_pie
Hapmap27031-BTA-161389	0,5497927	2	bru_pie
ARS-BFGL-NGS-54907	0,4987952	2	bru_pie
ARS-BFGL-NGS-34923	0,4758882	2	bru_pie
Hapmap24165-BTA-60897	0,4540095	2	bru_pie
ARS-BFGL-NGS-102609	0,502253	2	bru_pie
Hapmap44395-BTA-64864	0,4139436	3	bru_pie
BTB-01923614	0,3930325	3	bru_pie
ARS-BFGL-NGS-61702	0,490203	3	bru_pie
ARS-BFGL-NGS-73201	0,6470819	3	bru_pie
ARS-BFGL-NGS-11281	0,4935092	3	bru_pie
ARS-BFGL-NGS-80555	0,4705736	3	bru_pie
Hapmap32220-BTC-042831	0,6117028	3	bru_pie
Hapmap31616-BTC-042811	0,6720923	3	bru_pie
Hapmap56688-rs29025335	0,6225131	3	bru_pie
Hapmap38580-BTA-122605	0,5054258	3	bru_pie
Hapmap40806-BTA-77302	0,4306957	3	bru_pie
ARS-BFGL-NGS-107597	0,4495078	3	bru_pie
ARS-BFGL-NGS-4815	0,4956461	3	bru_pie
ARS-BFGL-NGS-31386	0,55916	3	bru_pie
ARS-BFGL-NGS-13803	0,7389009	3	bru_pie
ARS-BFGL-NGS-74800	0,4044331	3	bru_pie

ARS-BFGL-NGS-1097	0,3850229	3	bru_pie
ARS-BFGL-NGS-110975	0,4470808	3	bru_pie
ARS-BFGL-NGS-34923	0,4231008	3	bru_pie
ARS-BFGL-NGS-112454	0,3551958	4	bru_pie
ARS-BFGL-NGS-11319	0,3968593	4	bru_pie
Hapmap47560-BTA-30470	0,4008099	4	bru_pie
Hapmap55212-rs29013415	0,4615526	4	bru_pie
ARS-BFGL-NGS-119151	0,3376369	4	bru_pie
ARS-BFGL-NGS-103527	0,3384928	4	bru_pie
BTA-113850-no-rs	0,3022863	4	bru_pie
ARS-BFGL-NGS-42028	0,4054862	4	bru_pie
BTB-00979302	0,3812826	4	bru_pie
Hapmap40716-BTA-34647	0,664938	1	fri_mar
ARS-BFGL-NGS-102149	0,6107297	1	fri_mar
ARS-BFGL-NGS-105427	0,7537367	1	fri_mar
ARS-BFGL-NGS-12670	0,7030417	1	fri_mar
Hapmap58409-rs29020726	0,7255619	1	fri_mar
Hapmap42080-BTA-115228	0,6306653	1	fri_mar
ARS-BFGL-NGS-110085	0,7268444	1	fri_mar
ARS-BFGL-NGS-37807	0,5951922	1	fri_mar
ARS-BFGL-NGS-16805	0,7946414	1	fri_mar
Hapmap53144-ss46525999	0,960147	1	fri_mar
ARS-BFGL-NGS-18303	0,8438817	1	fri_mar
ARS-BFGL-NGS-33546	0,6213246	1	fri_mar
ARS-BFGL-NGS-116590	0,9619391	1	fri_mar
ARS-BFGL-NGS-105631	0,6074869	1	fri_mar
ARS-BFGL-NGS-104746	0,6209201	1	fri_mar
ARS-BFGL-NGS-15787	0,7251364	1	fri_mar
BTA-74881-no-rs	0,6675708	1	fri_mar
ARS-BFGL-NGS-39379	0,8135405	1	fri_mar
ARS-BFGL-NGS-89264	0,6252258	1	fri_mar
Hapmap57625-rs29027071	0,5993908	1	fri_mar
Hapmap50117-BTA-81807	0,5778375	1	fri_mar
Hapmap60836-rs29027147	0,5788707	1	fri_mar
ARS-BFGL-NGS-119539	0,6107756	1	fri_mar
Hapmap49309-BTA-78604	0,6857792	1	fri_mar
BTB-00300220	0,7010296	1	fri_mar
Hapmap42327-BTA-78943	0,6650354	1	fri_mar
ARS-BFGL-NGS-116933	0,6581692	1	fri_mar
BTB-00313206	0,737734	1	fri_mar
Hapmap51050-BTA-79767	0,6638049	1	fri_mar
Hapmap35664-SCAFFOLD116678_816	0,5868671	1	fri_mar
ARS-BFGL-NGS-23700	0,6450224	1	fri_mar
ARS-BFGL-NGS-110168	0,6037988	1	fri_mar
BTB-01632102	0,5925627	1	fri_mar
ARS-BFGL-NGS-102016	0,6388606	1	fri_mar
BTA-16517-no-rs	0,6269909	1	fri_mar
BTB-01332940	0,7059931	1	fri_mar
ARS-BFGL-NGS-112949	0,623895	1	fri_mar
ARS-BFGL-NGS-55763	0,720933	1	fri_mar
Hapmap56826-rs29013564	0,6212727	1	fri_mar
ARS-BFGL-NGS-83446	0,8101838	1	fri_mar
BTB-01530788	0,7214745	1	fri_mar
BTB-01530836	0,7177496	1	fri_mar
BTB-00557585	0,7442591	1	fri_mar
BTB-00557532	0,7655035	1	fri_mar

Hapmap41234-BTA-34285	0,7146203	1	fri_mar
Hapmap36131-SCAFFOLD280927_4409	0,6797111	1	fri_mar
Hapmap52387-rs29021226	0,6711966	1	fri_mar
ARS-BFGL-NGS-11296	0,596172	1	fri_mar
ARS-BFGL-NGS-24805	0,6253266	1	fri_mar
Hapmap41529-BTA-40591	0,6234888	1	fri_mar
Hapmap40057-BTA-25640	0,758145	1	fri_mar
ARS-BFGL-NGS-118491	0,6114943	1	fri_mar
Hapmap34713-BES10_Contig634_914	0,5980261	1	fri_mar
ARS-BFGL-NGS-86599	0,7457531	1	fri_mar
Hapmap44238-BTA-42338	0,8433857	1	fri_mar
ARS-BFGL-NGS-24837	0,8341293	1	fri_mar
Hapmap49615-BTA-44891	0,6958406	1	fri_mar
ARS-BFGL-NGS-10108	0,7193144	1	fri_mar
BTA-50702-no-rs	0,8122473	1	fri_mar
BTA-51687-no-rs	0,663332	1	fri_mar
Hapmap52964-rs29016827	0,6813172	1	fri_mar
ARS-BFGL-NGS-72462	0,723658	1	fri_mar
ARS-BFGL-NGS-111442	0,702208	1	fri_mar
BTA-57201-no-rs	0,6697776	1	fri_mar
ARS-BFGL-NGS-73903	0,6930115	1	fri_mar
Hapmap57192-rs29027634	0,7708651	1	fri_mar
Hapmap33073-BTA-162864	0,7352503	1	fri_mar
ARS-BFGL-NGS-116481	0,7152883	1	fri_mar
ARS-BFGL-NGS-6259	0,7064819	1	fri_mar
Hapmap53405-rs29014382	0,8713812	1	fri_mar
ARS-BFGL-NGS-101647	0,8877708	1	fri_mar
Hapmap24165-BTA-60897	0,7142751	1	fri_mar
ARS-BFGL-NGS-11271	0,8556078	1	fri_mar
BTB-00979271	0,7066857	1	fri_mar
Hapmap43004-BTA-64153	0,6162095	1	fri_mar
ARS-BFGL-NGS-110358	0,6722806	1	fri_mar
ARS-BFGL-NGS-38896	0,7266489	1	fri_mar
ARS-BFGL-NGS-59421	0,7443347	1	fri_mar
ARS-BFGL-NGS-37441	0,7089464	1	fri_mar
ARS-BFGL-NGS-29040	0,6651058	1	fri_mar
BTB-01033227	0,7682644	1	fri_mar
ARS-BFGL-NGS-14337	0,6939872	1	fri_mar
Hapmap39204-BTA-47760	0,4202566	2	fri_mar
Hapmap42558-BTA-47788	0,5663018	2	fri_mar
Hapmap59807-rs29011612	0,4340111	2	fri_mar
Hapmap60325-rs29019351	0,5639215	2	fri_mar
ARS-BFGL-NGS-20354	0,5883513	2	fri_mar
ARS-BFGL-NGS-107038	0,5016099	2	fri_mar
ARS-BFGL-BAC-14372	0,4683442	2	fri_mar
BTA-33373-no-rs	0,5422195	2	fri_mar
ARS-BFGL-NGS-3823	0,5213345	2	fri_mar
BTB-01120359	0,4610077	2	fri_mar
Hapmap48733-BTA-36980	0,4928559	2	fri_mar
BTB-01681968	0,5309286	2	fri_mar
ARS-BFGL-BAC-36986	0,4988945	2	fri_mar
ARS-BFGL-BAC-27962	0,5184964	2	fri_mar
BTB-00742339	0,4879395	2	fri_mar
BTB-01610069	0,54698	2	fri_mar
ARS-BFGL-BAC-29149	0,5661515	2	fri_mar
Hapmap53021-rs29009645	0,459272	2	fri_mar

Hapmap24165-BTA-60897	0,4540095	2	fri_mar
BTA-61748-no-rs	0,4532452	2	fri_mar
ARS-BFGL-NGS-5027	0,4374747	2	fri_mar
ARS-BFGL-NGS-30729	0,4084377	3	fri_mar
Hapmap56688-rs29025335	0,6225131	3	fri_mar
ARS-BFGL-NGS-12557	0,7294468	3	fri_mar
ARS-BFGL-NGS-20141	0,4259094	3	fri_mar
BTB-00310752	0,443132	3	fri_mar
ARS-BFGL-NGS-10500	0,4680059	3	fri_mar
ARS-BFGL-NGS-31386	0,55916	3	fri_mar
ARS-BFGL-NGS-41686	0,4691665	3	fri_mar
ARS-BFGL-NGS-112180	0,3744152	4	fri_mar
ARS-BFGL-NGS-43714	0,3138432	4	fri_mar
BTA-95639-no-rs	0,568662	1	fri_pez
Hapmap26500-BTA-37512	0,6107086	1	fri_pez
BTB-00034743	0,5926419	1	fri_pez
ARS-BFGL-NGS-25522	0,5771729	1	fri_pez
BTB-00048035	0,5774561	1	fri_pez
ARS-BFGL-NGS-100636	0,6579928	1	fri_pez
ARS-BFGL-NGS-119065	0,661461	1	fri_pez
ARS-BFGL-NGS-116391	0,7622641	1	fri_pez
ARS-BFGL-NGS-102149	0,6107297	1	fri_pez
ARS-BFGL-NGS-105427	0,7537367	1	fri_pez
ARS-BFGL-NGS-74724	0,654843	1	fri_pez
ARS-BFGL-NGS-12670	0,7030417	1	fri_pez
Hapmap58409-rs29020726	0,7255619	1	fri_pez
ARS-BFGL-NGS-110085	0,7268444	1	fri_pez
ARS-BFGL-NGS-37807	0,5951922	1	fri_pez
ARS-BFGL-NGS-16805	0,7946414	1	fri_pez
Hapmap53144-ss46525999	0,960147	1	fri_pez
ARS-BFGL-NGS-18303	0,8438817	1	fri_pez
ARS-BFGL-NGS-33546	0,6213246	1	fri_pez
ARS-BFGL-NGS-116590	0,9619391	1	fri_pez
ARS-BFGL-NGS-105631	0,6074869	1	fri_pez
ARS-BFGL-NGS-104746	0,6209201	1	fri_pez
BTA-74881-no-rs	0,6675708	1	fri_pez
ARS-BFGL-NGS-39379	0,8135405	1	fri_pez
ARS-BFGL-NGS-89264	0,6252258	1	fri_pez
Hapmap50117-BTA-81807	0,5778375	1	fri_pez
Hapmap60836-rs29027147	0,5788707	1	fri_pez
ARS-BFGL-NGS-119539	0,6107756	1	fri_pez
ARS-BFGL-NGS-74330	0,6877488	1	fri_pez
Hapmap42327-BTA-78943	0,6650354	1	fri_pez
ARS-BFGL-NGS-116933	0,6581692	1	fri_pez
BTB-00313206	0,737734	1	fri_pez
Hapmap51050-BTA-79767	0,6638049	1	fri_pez
ARS-BFGL-NGS-23700	0,6450224	1	fri_pez
BTB-01632102	0,5925627	1	fri_pez
BTB-00404802	0,6303729	1	fri_pez
ARS-BFGL-NGS-60179	0,6392158	1	fri_pez
ARS-BFGL-NGS-33594	0,6187352	1	fri_pez
ARS-BFGL-NGS-28924	0,6180156	1	fri_pez
ARS-BFGL-NGS-114512	0,6030026	1	fri_pez
BTB-01530788	0,7214745	1	fri_pez
BTB-01530836	0,7177496	1	fri_pez
BTB-00557585	0,7442591	1	fri_pez

BTB-00557532	0,7655035	1	fri_pez
Hapmap41234-BTA-34285	0,7146203	1	fri_pez
Hapmap36131-SCAFFOLD280927_4409	0,6797111	1	fri_pez
ARS-BFGL-NGS-24805	0,6253266	1	fri_pez
Hapmap41529-BTA-40591	0,6234888	1	fri_pez
Hapmap40057-BTA-25640	0,758145	1	fri_pez
ARS-BFGL-NGS-118491	0,6114943	1	fri_pez
Hapmap34713-BES10_Contig634_914	0,5980261	1	fri_pez
ARS-BFGL-NGS-24012	0,6523467	1	fri_pez
ARS-BFGL-NGS-86599	0,7457531	1	fri_pez
Hapmap44238-BTA-42338	0,8433857	1	fri_pez
ARS-BFGL-NGS-24837	0,8341293	1	fri_pez
Hapmap49615-BTA-44891	0,6958406	1	fri_pez
BTA-50702-no-rs	0,8122473	1	fri_pez
BTB-01176930	0,654425	1	fri_pez
ARS-BFGL-NGS-64419	0,6551707	1	fri_pez
BTA-53922-no-rs	0,6769278	1	fri_pez
BTA-57201-no-rs	0,6697776	1	fri_pez
ARS-BFGL-NGS-18676	0,7260827	1	fri_pez
ARS-BFGL-NGS-73903	0,6930115	1	fri_pez
Hapmap57192-rs29027634	0,7708651	1	fri_pez
ARS-BFGL-NGS-116481	0,7152883	1	fri_pez
ARS-BFGL-NGS-6259	0,7064819	1	fri_pez
Hapmap53405-rs29014382	0,8713812	1	fri_pez
ARS-BFGL-NGS-101647	0,8877708	1	fri_pez
Hapmap24165-BTA-60897	0,7142751	1	fri_pez
ARS-BFGL-NGS-11271	0,8556078	1	fri_pez
ARS-BFGL-NGS-60861	0,5973874	1	fri_pez
ARS-BFGL-NGS-39846	0,6242062	1	fri_pez
Hapmap35718-SCAFFOLD271203_2920	0,5636751	1	fri_pez
ARS-BFGL-NGS-18057	0,636203	1	fri_pez
Hapmap43004-BTA-64153	0,6162095	1	fri_pez
ARS-BFGL-NGS-38896	0,7266489	1	fri_pez
ARS-BFGL-NGS-59421	0,7443347	1	fri_pez
ARS-BFGL-NGS-37441	0,7089464	1	fri_pez
ARS-BFGL-NGS-113797	0,6640138	1	fri_pez
ARS-BFGL-NGS-29040	0,6651058	1	fri_pez
BTB-01033227	0,7682644	1	fri_pez
ARS-BFGL-NGS-14337	0,6939872	1	fri_pez
BTA-95639-no-rs	0,5620555	2	fri_pez
BTB-00047642	0,4864703	2	fri_pez
BTB-00047563	0,4857291	2	fri_pez
ARS-BFGL-NGS-101054	0,4674435	2	fri_pez
Hapmap26308-BTC-057761	0,7880093	2	fri_pez
Hapmap27083-BTC-041166	0,6402448	2	fri_pez
Hapmap23507-BTC-041133	0,5962648	2	fri_pez
Hapmap31285-BTC-041097	0,6185824	2	fri_pez
BTB-01581546	0,4742124	2	fri_pez
ARS-BFGL-NGS-4670	0,4189025	2	fri_pez
Hapmap24165-BTA-60897	0,4540095	2	fri_pez
Hapmap43863-BTA-47554	0,5009512	3	fri_pez
ARS-BFGL-NGS-35482	0,4500869	3	fri_pez
ARS-BFGL-NGS-30729	0,4084377	3	fri_pez
ARS-BFGL-NGS-102464	0,4577744	3	fri_pez
Hapmap38580-BTA-122605	0,5054258	3	fri_pez
Hapmap51338-BTA-87881	0,4179326	3	fri_pez

BTA-87878-no-rs	0,4174928	3	fri_pez
ARS-BFGL-NGS-42841	0,3897657	3	fri_pez
ARS-BFGL-NGS-52654	0,4265274	3	fri_pez
ARS-BFGL-NGS-19848	0,4064174	3	fri_pez
Hapmap35357-SCAFFOLD220094_14112	0,3934404	3	fri_pez
Hapmap39146-BTA-89809	0,4715696	3	fri_pez
ARS-BFGL-NGS-107597	0,4495078	3	fri_pez
ARS-BFGL-NGS-103244	0,4564996	3	fri_pez
ARS-BFGL-NGS-31386	0,55916	3	fri_pez
ARS-BFGL-NGS-13803	0,7389009	3	fri_pez
ARS-BFGL-NGS-41686	0,4691665	3	fri_pez
BTB-00034522	0,3537269	4	fri_pez
ARS-BFGL-NGS-112180	0,3744152	4	fri_pez
BTB-00285741	0,3061581	4	fri_pez
BTB-00048035	0,5774561	1	pez_mar
ARS-BFGL-NGS-119065	0,661461	1	pez_mar
Hapmap58409-rs29020726	0,7255619	1	pez_mar
ARS-BFGL-NGS-43280	0,6849637	1	pez_mar
Hapmap57625-rs29027071	0,5993908	1	pez_mar
ARS-BFGL-NGS-119539	0,6107756	1	pez_mar
Hapmap49309-BTA-78604	0,6857792	1	pez_mar
BTB-00300220	0,7010296	1	pez_mar
ARS-BFGL-NGS-74330	0,6877488	1	pez_mar
ARS-BFGL-NGS-33594	0,6187352	1	pez_mar
BTA-16517-no-rs	0,6269909	1	pez_mar
BTB-01332940	0,7059931	1	pez_mar
ARS-BFGL-NGS-24012	0,6523467	1	pez_mar
ARS-BFGL-NGS-86599	0,7457531	1	pez_mar
Hapmap44238-BTA-42338	0,8433857	1	pez_mar
ARS-BFGL-NGS-24837	0,8341293	1	pez_mar
Hapmap42401-BTA-102906	0,7244402	1	pez_mar
Hapmap52964-rs29016827	0,6813172	1	pez_mar
ARS-BFGL-NGS-72462	0,723658	1	pez_mar
ARS-BFGL-NGS-18057	0,636203	1	pez_mar
Hapmap59145-ss46526952	0,503495	2	pez_mar
Hapmap48672-BTA-17044	0,4906196	2	pez_mar
BTB-00047642	0,4864703	2	pez_mar
BTB-00047563	0,4857291	2	pez_mar
Hapmap42558-BTA-47788	0,5663018	2	pez_mar
Hapmap33253-BTA-160512	0,5160511	2	pez_mar
ARS-BFGL-NGS-2985	0,4738744	2	pez_mar
BTB-01934112	0,4972097	2	pez_mar
BTB-01144391	0,4994017	2	pez_mar
BTA-97585-no-rs	0,5044966	2	pez_mar
ARS-BFGL-NGS-112184	0,5655324	2	pez_mar
Hapmap27072-BTC-033816	0,5926193	2	pez_mar
Hapmap26308-BTC-057761	0,7880093	2	pez_mar
Hapmap27083-BTC-041166	0,6402448	2	pez_mar
Hapmap23507-BTC-041133	0,5962648	2	pez_mar
Hapmap31285-BTC-041097	0,6185824	2	pez_mar
ARS-BFGL-NGS-109884	0,6518992	2	pez_mar
BTA-81064-no-rs	0,4757058	2	pez_mar
ARS-BFGL-NGS-4606	0,5282284	2	pez_mar
ARS-BFGL-NGS-117820	0,509884	2	pez_mar
Hapmap30504-BTA-126653	0,5223383	2	pez_mar
ARS-BFGL-NGS-64740	0,5144729	2	pez_mar

ARS-BFGL-NGS-103217	0,5130644	2	pez_mar
Hapmap54972-rs29014977	0,5366218	2	pez_mar
BTA-33373-no-rs	0,5422195	2	pez_mar
ARS-BFGL-NGS-3823	0,5213345	2	pez_mar
ARS-BFGL-NGS-27920	0,4578273	2	pez_mar
BTB-01681968	0,5309286	2	pez_mar
ARS-BFGL-BAC-36986	0,4988945	2	pez_mar
ARS-BFGL-BAC-27962	0,5184964	2	pez_mar
BTB-00742339	0,4879395	2	pez_mar
BTB-01610069	0,54698	2	pez_mar
ARS-BFGL-BAC-29149	0,5661515	2	pez_mar
Hapmap31875-BTA-58099	0,5806762	2	pez_mar
ARS-BFGL-NGS-4670	0,4189025	2	pez_mar
ARS-BFGL-NGS-5027	0,4374747	2	pez_mar
BTB-00182760	0,4685279	3	pez_mar
ARS-BFGL-NGS-21133	0,4545024	3	pez_mar
ARS-BFGL-NGS-77601	0,4400112	3	pez_mar
ARS-BFGL-NGS-73201	0,6470819	3	pez_mar
ARS-BFGL-NGS-11281	0,4935092	3	pez_mar
ARS-BFGL-NGS-80555	0,4705736	3	pez_mar
Hapmap33128-BTC-041916	0,6216857	3	pez_mar
ARS-BFGL-NGS-38827	0,6210556	3	pez_mar
Hapmap27692-BTC-042876	0,6231059	3	pez_mar
Hapmap32220-BTC-042831	0,6117028	3	pez_mar
ARS-BFGL-NGS-12557	0,7294468	3	pez_mar
ARS-BFGL-NGS-20141	0,4259094	3	pez_mar
BTB-00310752	0,443132	3	pez_mar
BTB-00362255	0,4340817	3	pez_mar
Hapmap47422-BTA-82035	0,4518154	3	pez_mar
ARS-BFGL-NGS-107597	0,4495078	3	pez_mar
BTA-31880-no-rs	0,5052381	3	pez_mar
BTB-00591691	0,4991841	3	pez_mar
ARS-BFGL-NGS-117289	0,4002148	3	pez_mar
ARS-BFGL-NGS-31386	0,55916	3	pez_mar
ARS-BFGL-NGS-13803	0,7389009	3	pez_mar
ARS-BFGL-NGS-1097	0,3850229	3	pez_mar
ARS-BFGL-NGS-1498	0,4926483	3	pez_mar
Hapmap38686-BTA-61607	0,4851001	3	pez_mar
Hapmap50491-BTA-85929	0,4206083	4	pez_mar
Hapmap42446-BTA-118372	0,3462518	4	pez_mar
BTB-01462011	0,6843797	1	pie_fri
BTB-00030699	0,5754362	1	pie_fri
Hapmap40716-BTA-34647	0,664938	1	pie_fri
BTA-95639-no-rs	0,568662	1	pie_fri
BTA-36624-no-rs	0,586048	1	pie_fri
Hapmap26500-BTA-37512	0,6107086	1	pie_fri
BTB-00034743	0,5926419	1	pie_fri
ARS-BFGL-NGS-25522	0,5771729	1	pie_fri
ARS-BFGL-BAC-2576	0,697632	1	pie_fri
ARS-BFGL-NGS-4516	0,6226637	1	pie_fri
ARS-BFGL-NGS-100636	0,6579928	1	pie_fri
ARS-BFGL-NGS-54356	0,6578662	1	pie_fri
ARS-BFGL-NGS-14778	0,6657053	1	pie_fri
ARS-BFGL-NGS-119065	0,661461	1	pie_fri
ARS-BFGL-NGS-14073	0,6781325	1	pie_fri
ARS-BFGL-NGS-116391	0,7622641	1	pie_fri

ARS-BFGL-NGS-102149	0,6107297	1	pie_fri
ARS-BFGL-NGS-105427	0,7537367	1	pie_fri
ARS-BFGL-NGS-12670	0,7030417	1	pie_fri
ARS-BFGL-NGS-114035	0,6562947	1	pie_fri
BTA-16657-no-rs	0,5942014	1	pie_fri
Hapmap58409-rs29020726	0,7255619	1	pie_fri
ARS-BFGL-NGS-110085	0,7268444	1	pie_fri
ARS-BFGL-NGS-37807	0,5951922	1	pie_fri
ARS-BFGL-NGS-16805	0,7946414	1	pie_fri
Hapmap53144-ss46525999	0,960147	1	pie_fri
ARS-BFGL-NGS-18303	0,8438817	1	pie_fri
ARS-BFGL-NGS-33546	0,6213246	1	pie_fri
ARS-BFGL-NGS-116590	0,9619391	1	pie_fri
ARS-BFGL-NGS-105631	0,6074869	1	pie_fri
BTB-01267042	0,615709	1	pie_fri
BTA-74881-no-rs	0,6675708	1	pie_fri
ARS-BFGL-NGS-39379	0,8135405	1	pie_fri
ARS-BFGL-NGS-89264	0,6252258	1	pie_fri
Hapmap50117-BTA-81807	0,5778375	1	pie_fri
ARS-BFGL-NGS-119539	0,6107756	1	pie_fri
Hapmap51409-BTA-122717	0,606841	1	pie_fri
Hapmap49309-BTA-78604	0,6857792	1	pie_fri
BTB-00300220	0,7010296	1	pie_fri
Hapmap42327-BTA-78943	0,6650354	1	pie_fri
BTB-00310975	0,6804365	1	pie_fri
ARS-BFGL-NGS-116933	0,6581692	1	pie_fri
Hapmap35664-SCAFFOLD116678_816	0,5868671	1	pie_fri
ARS-BFGL-NGS-23700	0,6450224	1	pie_fri
BTA-82649-no-rs	0,6072671	1	pie_fri
BTB-01632102	0,5925627	1	pie_fri
Hapmap34494-BES3_Contig286_1171	0,6079334	1	pie_fri
ARS-BFGL-NGS-60179	0,6392158	1	pie_fri
Hapmap45147-BTA-106533	0,6697748	1	pie_fri
ARS-BFGL-NGS-19567	0,6285041	1	pie_fri
ARS-BFGL-NGS-55763	0,720933	1	pie_fri
ARS-BFGL-NGS-33209	0,6628599	1	pie_fri
BTB-01530788	0,7214745	1	pie_fri
BTB-01530836	0,7177496	1	pie_fri
BTB-00557585	0,7442591	1	pie_fri
BTB-00557532	0,7655035	1	pie_fri
Hapmap41234-BTA-34285	0,7146203	1	pie_fri
Hapmap36131-SCAFFOLD280927_4409	0,6797111	1	pie_fri
Hapmap46938-BTA-114095	0,6835801	1	pie_fri
Hapmap52387-rs29021226	0,6711966	1	pie_fri
ARS-BFGL-NGS-24805	0,6253266	1	pie_fri
Hapmap41529-BTA-40591	0,6234888	1	pie_fri
Hapmap40057-BTA-25640	0,758145	1	pie_fri
ARS-BFGL-NGS-118491	0,6114943	1	pie_fri
Hapmap34713-BES10_Contig634_914	0,5980261	1	pie_fri
ARS-BFGL-NGS-24012	0,6523467	1	pie_fri
ARS-BFGL-NGS-114129	0,5898711	1	pie_fri
Hapmap40737-BTA-44406	0,5903902	1	pie_fri
ARS-BFGL-NGS-86599	0,7457531	1	pie_fri
Hapmap44238-BTA-42338	0,8433857	1	pie_fri
ARS-BFGL-NGS-24837	0,8341293	1	pie_fri
ARS-BFGL-NGS-80289	0,7315056	1	pie_fri

Hapmap49615-BTA-44891	0,6958406	1	pie_fri
ARS-BFGL-NGS-40523	0,761229	1	pie_fri
BTA-50702-no-rs	0,8122473	1	pie_fri
ARS-BFGL-NGS-111442	0,702208	1	pie_fri
ARS-BFGL-NGS-18676	0,7260827	1	pie_fri
ARS-BFGL-NGS-73903	0,6930115	1	pie_fri
Hapmap57192-rs29027634	0,7708651	1	pie_fri
ARS-BFGL-NGS-116481	0,7152883	1	pie_fri
ARS-BFGL-NGS-6259	0,7064819	1	pie_fri
Hapmap53405-rs29014382	0,8713812	1	pie_fri
ARS-BFGL-NGS-101647	0,8877708	1	pie_fri
Hapmap24165-BTA-60897	0,7142751	1	pie_fri
ARS-BFGL-NGS-11271	0,8556078	1	pie_fri
ARS-BFGL-NGS-39846	0,6242062	1	pie_fri
Hapmap35718-SCAFFOLD271203_2920	0,5636751	1	pie_fri
ARS-BFGL-NGS-75049	0,6853566	1	pie_fri
Hapmap43004-BTA-64153	0,6162095	1	pie_fri
ARS-BFGL-NGS-110358	0,6722806	1	pie_fri
ARS-BFGL-NGS-38896	0,7266489	1	pie_fri
ARS-BFGL-NGS-59421	0,7443347	1	pie_fri
ARS-BFGL-NGS-37441	0,7089464	1	pie_fri
ARS-BFGL-NGS-113797	0,6640138	1	pie_fri
ARS-BFGL-NGS-29040	0,6651058	1	pie_fri
BTB-01033227	0,7682644	1	pie_fri
ARS-BFGL-NGS-14337	0,6939872	1	pie_fri
BTA-95639-no-rs	0,5620555	2	pie_fri
ARS-BFGL-NGS-20354	0,5883513	2	pie_fri
ARS-BFGL-NGS-109884	0,6518992	2	pie_fri
BTB-01581546	0,4742124	2	pie_fri
ARS-BFGL-NGS-64740	0,5144729	2	pie_fri
ARS-BFGL-NGS-103217	0,5130644	2	pie_fri
Hapmap24165-BTA-60897	0,4540095	2	pie_fri
ARS-BFGL-NGS-5027	0,4374747	2	pie_fri
ARS-BFGL-NGS-30729	0,4084377	3	pie_fri
Hapmap38085-BTA-87616	0,4939803	3	pie_fri
Hapmap33128-BTC-041916	0,6216857	3	pie_fri
ARS-BFGL-NGS-38827	0,6210556	3	pie_fri
Hapmap27692-BTC-042876	0,6231059	3	pie_fri
Hapmap32220-BTC-042831	0,6117028	3	pie_fri
ARS-BFGL-NGS-12557	0,7294468	3	pie_fri
ARS-BFGL-NGS-20141	0,4259094	3	pie_fri
BTB-00310752	0,443132	3	pie_fri
Hapmap35357-SCAFFOLD220094_14112	0,3934404	3	pie_fri
ARS-BFGL-NGS-10500	0,4680059	3	pie_fri
Hapmap39146-BTA-89809	0,4715696	3	pie_fri
ARS-BFGL-NGS-83068	0,4444689	3	pie_fri
ARS-BFGL-NGS-103244	0,4564996	3	pie_fri
BTA-16884-no-rs	0,4944065	3	pie_fri
BTA-36883-no-rs	0,4346694	3	pie_fri
ARS-BFGL-NGS-31386	0,55916	3	pie_fri
ARS-BFGL-NGS-44155	0,4500728	3	pie_fri
ARS-BFGL-NGS-41686	0,4691665	3	pie_fri
ARS-BFGL-NGS-112454	0,3551958	4	pie_fri
ARS-BFGL-NGS-11319	0,3968593	4	pie_fri
Hapmap47560-BTA-30470	0,4008099	4	pie_fri
BTB-01162037	0,2829482	4	pie_fri

BTA-76070-no-rs	0,3967672	4	pie_fri
BTB-00313140	0,3332407	4	pie_fri
BTB-00285741	0,3061581	4	pie_fri
BTA-31807-no-rs	0,4019052	4	pie_fri
ARS-BFGL-NGS-30853	0,306268	4	pie_fri
ARS-BFGL-NGS-14014	0,4025394	4	pie_fri
Hapmap35702-SCAFFOLD240765_1255	0,358391	4	pie_fri
ARS-BFGL-BAC-2576	0,697632	1	pie_mar
ARS-BFGL-NGS-4516	0,6226637	1	pie_mar
ARS-BFGL-NGS-43280	0,6849637	1	pie_mar
Hapmap57625-rs29027071	0,5993908	1	pie_mar
BTA-75919-no-rs	0,5740664	1	pie_mar
BTB-01332940	0,7059931	1	pie_mar
ARS-BFGL-NGS-112949	0,623895	1	pie_mar
ARS-BFGL-NGS-24012	0,6523467	1	pie_mar
Hapmap52964-rs29016827	0,6813172	1	pie_mar
ARS-BFGL-NGS-72462	0,723658	1	pie_mar
BTA-57141-no-rs	0,7177956	1	pie_mar
BTA-57201-no-rs	0,6697776	1	pie_mar
ARS-BFGL-NGS-18676	0,7260827	1	pie_mar
Hapmap51005-BTA-60474	0,6190438	1	pie_mar
Hapmap42558-BTA-47788	0,5663018	2	pie_mar
Hapmap59807-rs29011612	0,4340111	2	pie_mar
Hapmap60325-rs29019351	0,5639215	2	pie_mar
BTB-01934112	0,4972097	2	pie_mar
ARS-BFGL-NGS-62562	0,5862706	2	pie_mar
ARS-BFGL-NGS-112184	0,5655324	2	pie_mar
Hapmap26308-BTC-057761	0,7880093	2	pie_mar
Hapmap27083-BTC-041166	0,6402448	2	pie_mar
Hapmap23507-BTC-041133	0,5962648	2	pie_mar
Hapmap31285-BTC-041097	0,6185824	2	pie_mar
ARS-BFGL-NGS-107038	0,5016099	2	pie_mar
Hapmap26151-BTA-146648	0,5207598	2	pie_mar
BTB-01678420	0,4831143	2	pie_mar
Hapmap30504-BTA-126653	0,5223383	2	pie_mar
ARS-BFGL-NGS-64740	0,5144729	2	pie_mar
ARS-BFGL-NGS-103217	0,5130644	2	pie_mar
Hapmap54972-rs29014977	0,5366218	2	pie_mar
BTA-33373-no-rs	0,5422195	2	pie_mar
ARS-BFGL-NGS-3823	0,5213345	2	pie_mar
BTB-01681968	0,5309286	2	pie_mar
ARS-BFGL-BAC-36986	0,4988945	2	pie_mar
ARS-BFGL-BAC-27962	0,5184964	2	pie_mar
BTB-00742339	0,4879395	2	pie_mar
ARS-BFGL-BAC-29149	0,5661515	2	pie_mar
Hapmap53021-rs29009645	0,459272	2	pie_mar
Hapmap30236-BTA-137040	0,4721366	2	pie_mar
ARS-BFGL-NGS-4670	0,4189025	2	pie_mar
ARS-BFGL-NGS-21133	0,4545024	3	pie_mar
ARS-BFGL-NGS-77601	0,4400112	3	pie_mar
ARS-BFGL-NGS-73201	0,6470819	3	pie_mar
ARS-BFGL-NGS-11281	0,4935092	3	pie_mar
ARS-BFGL-NGS-80555	0,4705736	3	pie_mar
Hapmap56688-rs29025335	0,6225131	3	pie_mar
ARS-BFGL-NGS-107597	0,4495078	3	pie_mar
ARS-BFGL-NGS-117289	0,4002148	3	pie_mar

Hapmap25302-BTA-150556	0,5469015	3	pie_mar
Hapmap30236-BTA-137040	0,3833773	3	pie_mar
BTA-85701-no-rs	0,5307967	4	pie_mar
ARS-BFGL-NGS-112454	0,3551958	4	pie_mar
ARS-BFGL-NGS-11319	0,3968593	4	pie_mar
Hapmap47560-BTA-30470	0,4008099	4	pie_mar
Hapmap55212-rs29013415	0,4615526	4	pie_mar
ARS-BFGL-NGS-119151	0,3376369	4	pie_mar
ARS-BFGL-NGS-103527	0,3384928	4	pie_mar
Hapmap52789-rs29018750	0,4072175	4	pie_mar
Hapmap50491-BTA-85929	0,4206083	4	pie_mar
BTA-36624-no-rs	0,586048	1	pie_pez
BTB-00048035	0,5774561	1	pie_pez
ARS-BFGL-BAC-2576	0,697632	1	pie_pez
ARS-BFGL-NGS-4516	0,6226637	1	pie_pez
BTA-16657-no-rs	0,5942014	1	pie_pez
Hapmap58409-rs29020726	0,7255619	1	pie_pez
BTA-75919-no-rs	0,5740664	1	pie_pez
Hapmap60836-rs29027147	0,5788707	1	pie_pez
ARS-BFGL-NGS-119539	0,6107756	1	pie_pez
Hapmap42327-BTA-78943	0,6650354	1	pie_pez
ARS-BFGL-NGS-33594	0,6187352	1	pie_pez
BTA-16517-no-rs	0,6269909	1	pie_pez
Hapmap36131-SCAFFOLD280927_4409	0,6797111	1	pie_pez
ARS-BFGL-NGS-86599	0,7457531	1	pie_pez
Hapmap44238-BTA-42338	0,8433857	1	pie_pez
ARS-BFGL-NGS-24837	0,8341293	1	pie_pez
Hapmap30844-BTA-148492	0,6038205	1	pie_pez
ARS-BFGL-NGS-18057	0,636203	1	pie_pez
BTB-00047642	0,4864703	2	pie_pez
BTB-00047563	0,4857291	2	pie_pez
Hapmap26308-BTC-057761	0,7880093	2	pie_pez
Hapmap27083-BTC-041166	0,6402448	2	pie_pez
Hapmap23507-BTC-041133	0,5962648	2	pie_pez
Hapmap31285-BTC-041097	0,6185824	2	pie_pez
ARS-BFGL-NGS-109884	0,6518992	2	pie_pez
BTA-81064-no-rs	0,4757058	2	pie_pez
Hapmap44323-BTA-96987	0,4661353	2	pie_pez
ARS-BFGL-NGS-4606	0,5282284	2	pie_pez
BTB-01289984	0,4363585	2	pie_pez
Hapmap29859-BTA-161589	0,5571473	2	pie_pez
BTB-00182760	0,4685279	3	pie_pez
Hapmap33484-BTA-142221	0,4423976	3	pie_pez
Hapmap33128-BTC-041916	0,6216857	3	pie_pez
ARS-BFGL-NGS-38827	0,6210556	3	pie_pez
Hapmap27692-BTC-042876	0,6231059	3	pie_pez
Hapmap32220-BTC-042831	0,6117028	3	pie_pez
Hapmap31616-BTC-042811	0,6720923	3	pie_pez
BTB-01560842	0,4411595	3	pie_pez
ARS-BFGL-NGS-12557	0,7294468	3	pie_pez
ARS-BFGL-NGS-20141	0,4259094	3	pie_pez
BTB-00310752	0,443132	3	pie_pez
BTA-111263-no-rs	0,4330121	3	pie_pez
Hapmap38580-BTA-122605	0,5054258	3	pie_pez
ARS-BFGL-NGS-52654	0,4265274	3	pie_pez
ARS-BFGL-NGS-19848	0,4064174	3	pie_pez

ARS-BFGL-NGS-27072	0,4308096	3	pie_pez
BTB-00591691	0,4991841	3	pie_pez
ARS-BFGL-NGS-31386	0,55916	3	pie_pez
ARS-BFGL-NGS-13803	0,7389009	3	pie_pez
BTA-35627-no-rs	0,3096588	4	pie_pez
ARS-BFGL-NGS-112454	0,3551958	4	pie_pez
ARS-BFGL-NGS-11319	0,3968593	4	pie_pez
Hapmap47560-BTA-30470	0,4008099	4	pie_pez
Hapmap55212-rs29013415	0,4615526	4	pie_pez
ARS-BFGL-NGS-119151	0,3376369	4	pie_pez
ARS-BFGL-NGS-103527	0,3384928	4	pie_pez
Hapmap22875-BTA-155031	0,2845661	4	pie_pez
Hapmap52789-rs29018750	0,4072175	4	pie_pez
Hapmap50491-BTA-85929	0,4206083	4	pie_pez
BTB-01899352	0,3720726	4	pie_pez
BTB-00262807	0,3721159	4	pie_pez
Hapmap50484-BTA-83557	0,3307087	4	pie_pez
ARS-BFGL-NGS-95080	0,3043731	4	pie_pez
Hapmap42710-BTA-86602	0,36196	4	pie_pez
ARS-BFGL-NGS-109752	0,3016756	4	pie_pez
ARS-BFGL-NGS-30853	0,306268	4	pie_pez
ARS-BFGL-NGS-113705	0,3164352	4	pie_pez
BTA-113850-no-rs	0,3022863	4	pie_pez
Hapmap42446-BTA-118372	0,3462518	4	pie_pez
ARS-BFGL-NGS-42028	0,4054862	4	pie_pez
